# Managed and Unmanaged *Pinus sylvestris* Forest Stands Harbour Similar Diversity and Composition of the Phyllosphere and Soil Fungi

**DOI:** 10.3390/microorganisms8020259

**Published:** 2020-02-15

**Authors:** Jūratė Lynikienė, Diana Marčiulynienė, Adas Marčiulynas, Artūras Gedminas, Miglė Vaičiukynė, Audrius Menkis

**Affiliations:** 1 Institute of Forestry, Lithuanian Research Centre for Agriculture and Forestry, Liepų str. 1, Girionys, LT-53101 Kaunas, Lithuania; jurate.lynikiene@mi.lt (J.L.); diana.marciulyniene@mi.lt (D.M.); adas.marciulynas@mi.lt (A.M.); m.apsauga@mi.lt (A.G.); migle.vaiciukyne@mi.lt (M.V.); 2 Department of Forest Mycology and Plant Pathology, Uppsala BioCenter, Swedish University of Agricultural Sciences, P.O. Box 7026, SE-75007 Uppsala, Sweden

**Keywords:** fungal biodiversity, community composition, anthropogenic effects, natural ecosystems, Scots pine

## Abstract

The aim was to assess fungal communities associated with living needles and soil of *Pinus sylvestris* in managed and unmanaged forest stands to get a better understanding of whether and how different intensities of forest management affects fungal diversity and community composition under the north temperate forest zone conditions. The study was carried out in three national parks in Lithuania. Each included five study sites in managed stands and five in unmanaged stands. At each site, three random soil cores and five random last-year needle samples were collected. Following DNA isolation, a DNA fragment of the ITS2 rRNA gene region of each sample was individually amplified and subjected to high-throughput sequencing. Analysis of 195,808 high-quality reads showed the presence of 1909 fungal taxa. Richness and composition of fungal taxa were similar in each substrate (needles and soil) in managed *vs*. unmanaged sites. The most common fungi in needles were *Coleosporium campanulae* (12.4% of all fungal sequences), Unidentified sp. 3980_1 (12.4%), Unidentified sp. 3980_4 (4.1%) and *Sydowia polyspora* (3.1%). In soil: Unidentified sp. 3980_21 (8.6%), *Umbelopsis nana* (8.2%), *Archaeorhizomyces* sp. 3980_5 (8.1%) and *Penicillium spinulosum* (6.3%). The results demonstrated that managed and unmanaged *P. sylvestris* stands support similar diversity and composition of fungal communities associated with living needles and soil.

## 1. Introduction

European forests are used for multiple purposes, which often include the potentially conflicting goals of timber production and biodiversity conservation [1]. To protect natural habitats and biodiversity, in many countries, the establishment of protected areas was initiated, where management activities are prohibited [2,3]. As a result, ca. 10% of the forest area in Europe is left unmanaged [4]. In unmanaged forests, natural disturbances such as wind throws or forest fires lead to heterogeneous structure and different successional stages of forest development [5,6,7,8]. Such forests usually contain old-growth trees and accumulate larger volumes of dead wood that provide valuable habitats and support biodiversity [5]. Unmanaged forests have been shown to have, in general, higher biological diversity than managed forests [5].

Intensive forest management may often result in drastic changes leading to loss of naturalness and biodiversity in forest ecosystems [9], causing remarkable changes to forest structure and function [2]. This may eventually lead to lower buffering capacity and adaptability of such forests to climate change. Clear-cuttings especially can lead to changes in tree age structure and species composition, and may also modify the microclimate and/or soil conditions, and ultimately affect the functioning of forest ecosystems [3]. These changes can reduce or even threaten different organisms that depend on natural forest habitats [5]. However, many of these forests may still have relatively high biodiversity [6]. For example, Horák et al. [4] have shown that some beetles thrive in managed forests. Indeed, the richness of several ground beetle species was found to be either higher or did not differ significantly when compared between managed and unmanaged stands [10,11,12,13]. Many of these beetles are considered as indicator species, reflecting habitat conditions [14].

In Europe, coniferous forests, including Scots pine (*Pinus sylvestris*), are widely distributed and are of enormous ecological and economic importance [15,16,17,18]. Although these forests generally have lower biodiversity than deciduous forests [4], *P. sylvestris* provides habitats to a large number of different organisms [19,20]. The phyllosphere, which in conifers is dominated by needles and is the key component responsible for photosynthesis and transpiration, represents one of the largest terrestrial habitats for microorganisms [21]. Fungi in this habitat represent the largest microbial component that may influence different physiological processes, such as tree growth and adaptation to different abiotic and biotic stress factors [22]. Soil fungi are equally important as many of these are responsible for decomposition of organic matter and nutrient recycling, while mutualistic mycorrhizal fungi may provide nutritional benefits to host trees [23,24,25]. However, fungal communities can be sensitive to different forest transformations, including changes in plant diversity, composition and structure [23,26]. Indeed, forest management may negatively affect fungal communities, resulting in lower diversity [3]. For example, regular timber harvesting results in soil that is more compact, which may cause changes in soil fungal community structure [23]. On the other hand, under certain conditions (depending on tree species, age and type of forest management), recurrent disturbances that occur in managed stands could lead to higher biodiversity [23,27]. The above observations suggest that the impact of forest management on fungal diversity and community composition may depend on different factors.

High-throughput sequencing methods provide powerful tools to explore fungal diversity directly from environmental samples [28]. While providing detailed and semi-quantitative information, they enable us to study the effects of different factors on fungal diversity and community composition [29,30]. However, we need to be aware of potential risks, including methodological biases, limitations of markers and bioinformatics challenges, and to learn possible solutions [31]. While different sequencing platforms have advantages and limitations, the Pacific Biosciences (PacBio) platform offers a low error rate by re-sequencing of circular molecules multiple times, especially for shorter reads [32], and thus, was used in the present study.

The aim of the present study was to assess fungal communities associated with living needles and soil of *P. sylvestris* in managed and unmanaged forest stands in Lithuania in order to get a better understanding on whether and how different intensities of forest management affect fungal diversity and community composition under the north temperate forest zone conditions. In Lithuania, forests occupy ca. 2.2 million ha, constituting 33.2% of the total land area. *P. sylvestris* is among the most important tree species as its stands occupy ca. 35% of the total forest area. Protected areas cover 16.6% of the country’s territory, where *P. sylvestris* stands occupy ca. 45% of this area [33].

## 2. Materials and Methods 

### 2.1. Study Sites and Sampling

**Figure 1:**
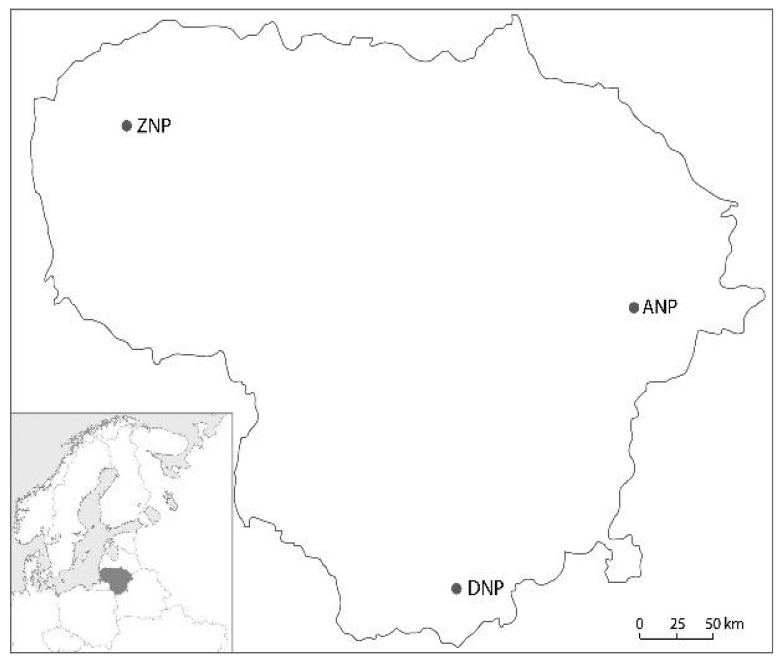
Map of Lithuania (position shown by shading on the north European map in the lower left corner) showing principal locations of the Aukštaitija national park (ANP), Dzūkija national park (DNP) and Žemaitija national park (ZNP), where sampling of living needles and soil was carried out in managed and unmanaged *Pinus sylvestris* forest stands.

The study sites were in *P. sylvestris* stands at Aukštaitija national park (ANP), Dzūkija national park (DNP) and Žemaitija national park (ZNP) in Lithuania (Figure 1). In each national park, five study sites were established in managed stands and five in unmanaged stands. Information on stand characteristics is in Table 1. Managed stands were stands used for commercial timber production. Unmanaged stands were stands left for natural development. Unmanaged stands were in the territory of three respective strict forest reserves, namely Girutiškis at ANP (established. in 1992 with a total area of 1394 ha), Skroblus at DNP (established in 1983 with a total area of 810 ha) and Plokštinė at ZNP (established in 1991 with a total area of 845 ha). After the establishment, the territory of each forest reserve was restricted to public access. In each national park, managed and unmanaged territories are separated by a buffer zone. Before the establishment of forest reserves, these areas were used for commercial timber production.

In each national park, both managed and unmanaged study sites were selected based on the forest inventory data. The criteria used for selection were: (a) close proximity of managed and unmanaged sites, (b) *P. sylvestris* being prevailing tree species, (c) similar soil type [34], and (d) similar vegetation type [35]. At ANP, study sites were on nutrient-poor, sandy soils with normal humidity of *vaccinium*- *myrtillosum* vegetation type; at DNP, these were on very poor sandy soil with normal humidity of *cladoniosum* forest type; and at ZNP, these were on moderately rich soil of light texture and normal humidity of *oxalidosum* forest type (Table 1). In each national park, managed and unmanaged sites were within a radius of 500 m, i.e., within the same geographical area and with similar climatic conditions.

At each site, sampling was carried out in October 2017 by taking three random soil samples and five random last-year living needle samples (Table 2). At the time of sampling, the mean monthly temperature and the total monthly precipitation were: 6.9 °C and 87 mm at ANP, 7.3 °C and 111 mm at DNP, and 6.9 °C and 161 mm at ZNP. For a sampling of soil, the upper litter layer was removed, and samples were taken down to 20 cm depth by using a 2 cm diameter soil core, which was carefully cleaned between individual samples. Each individual sample constituted ca. 100 g of soil. Each needle sample was collected from branches of an individual tree by randomly taking up to 25 healthy-looking needles. A telescopic secateurs was used to cut branches with needles from the middle part of crowns. Needles were sampled using forceps, which were cleaned between samples. Individual soil and needle samples were placed separately into plastic bags and labelled. The same day of sampling, samples were transported to the laboratory and placed in −20 °C for storage. A total of 150 needle samples and 90 soil samples were collected (Table 2). 

### 2.2. DNA Isolation and Sequencing

Principles of DNA work followed a study by Menkis et al. [36]. Prior to isolation of DNA, each sample (soil or needles) was freeze-dried at −60 °C for 2 days. For needles, no surface sterilization was carried out. Lyophilised needles were cut into smaller fractions and ca. 60 mg dry weight of each sample was placed into a 2-mL screw-cap centrifugation tube together with glass beads and homogenized using a Precellys 24 tissue homogenizer (Montigny-le-Bretonneux, France). DNA was isolated using CTAB extraction buffer (0.5 M EDTA pH 8.0, 1 M Tris-HCL pH 8.0, 5 M NaCl and 3% CTAB) followed by incubation at 65 °C for 1 h. After centrifugation, the supernatant was transferred to a new 1.5 mL Eppendorf tube and mixed with an equal volume of chloroform. Aqueous solution was transferred to a new tube, and an equal volume of 2-propanol was added to precipitate the DNA, which was pelleted by centrifugation. The pellet was washed in 500 μL 70% ethanol, dried, and dissolved in 50 μL of sterile milli-Q water. Within the same study site, samples were pooled together resulting in 30 DNA samples from the needles altogether (Table 2). Differing from needle samples, three lyophilised soil samples representing the same study site were mixed together prior to DNA extraction, resulting in 30 soil samples altogether (Table 2). For each sample, DNA extraction was done from 1 g dry-weight of soil using the NucleoSpin^®^Soil kit (Düren, Germany) according to the manufacturer’s instructions.

The DNA concentration in individual samples (needles and soil) was determined using a NanoDrop™ One spectrophotometer (Thermo Scientific, Rodchester, NY, USA) and adjusted to 1–10 ng/µL. Amplification by PCR of the ITS2 rRNA region was done using barcoded fungal specific primer gITS7 [37] and barcoded universal primer ITS4 [38]. PCR was performed in 50 μL reactions containing 2.5 µL of DNA template. Each reaction included 1% of DreamTaq Green Polymerase (5 μ/μL) (Thermo Scientific, Waltham, MA, USA); 11% of 10× Buffer; 11% of dNTPs (10 mM); 1% of MgCl2 (25 mM); 2% of each primer (200 nM) and 72% of milli-Q water. Amplifications were performed using the Applied Biosystems 2720 thermal cycler (Foster City, CA, USA). The PCR started with an initial denaturation at 95 °C for 5 min, followed by 30 cycles of 95 °C for 30 s, annealing at 56 °C for 30 s and 72 °C for 30 s, followed by a final extension step at 72 °C for 7 min. The PCR products were analyzed using gel electrophoresis on 1% agarose gels stained with Nancy-520 (Sigma-Aldrich, Stockholm, Sweden). PCR products were purified by centrifugation in 1:20 volume of 3 M sodium acetate (pH 5.2) (Applichem Gmbh, Darmstadt, Germany) and 96% ethanol mixture. Purified PCR products were quantified using a Qubit fluorometer 4.0 (Thermo Fisher Scientific, Waltham, MA, USA), and an equimolar mix of all PCR products was used for high-throughput sequencing using Pacific Biosciences platform (Menlo Park, CA, USA). Construction of the sequencing library and sequencing using Sequel II one SMRT cell was done at the SciLifeLab (Uppsala, Sweden).

### 2.3. Bioinformatics

The sequences generated were subjected to quality control and clustering in the SCATA NGS sequencing pipeline (http://scata.mykopat.slu.se). Quality filtering of the sequences included the removal of short sequences (<200 bp), sequences with low read quality, primer dimers and homopolymers, which were collapsed to 3 base pairs (bp) before clustering. Sequences that were missing a tag or primer were excluded. The primer and sample tags were then removed from the sequence, but information on the sequence association with the sample was stored as meta-data. The sequences were then clustered into different taxa using single-linkage clustering based on 98% similarity. The most common genotype (real read) for each cluster was used to represent each taxon. For clusters containing only two sequences, a consensus sequence was produced. Fungal taxa were taxonomically identified using both the RDP classifier available at https://pyro.cme.msu.edu/index.jsp (Centre for Microbial Ecology, Michigan State University, Michigan, USA) and GenBank (NCBI) database using the Blastn algorithm. The criteria used for identification were: sequence coverage >80%; similarity to taxon level 98–100%, similarity to genus level 94–97%. Sequences not matching these criteria were considered unidentified and were given unique names, as shown in Table 3 and Table 4 and Table S1. Representative sequences of all fungal non-singletons are available from GenBank under accession numbers MN902354 - MN904183.

### 2.4. Statistical Analyses

Rarefaction analysis was performed using Analytical Rarefaction v.1.3 available at http://www.uga.edu/strata/software/index.html. Differences in the richness of fungal taxa in managed and unmanaged forest sites (data pooled from all sites) were compared by nonparametric chi-square testing [39]. The Shannon diversity index, qualitative Sorensen similarity index and principal component analysis (PCA) in Canoco 5 [39,40,41] were used to characterize the diversity and composition of fungal communities. MANOVA in Minitab v. 18.1 (PA, USA) was used to evaluate the degree of separation between the fungal communities in needles and soil in managed and unmanaged sites, respectively, and between different types of substrates (needles and soil), when managed and unmanaged sites were combined together. The nonparametric Mann-Whitney test in Minitab was used to test if the Shannon diversity indexes between managed and unmanaged sites were statistically similar or not.

## 3. Results

**Figure 2:**
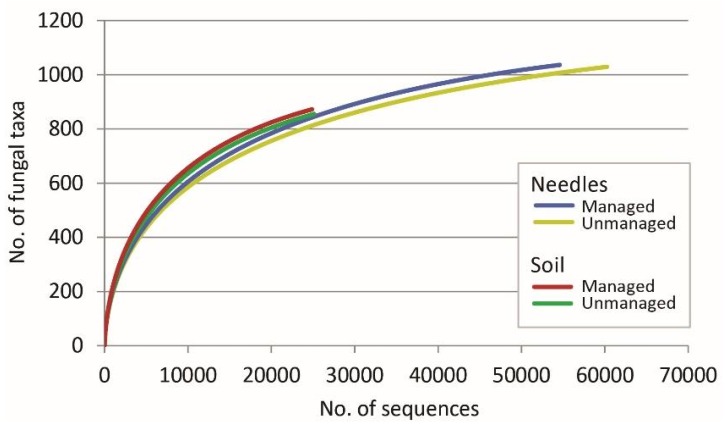
Rarefaction curves showing the relationship between the cumulative number of fungal taxa and the number of ITS2 rRNA sequences from living needles and soil from managed and unmanaged *Pinus sylvestris* forest stands.

High-throughput sequencing of fungal ITS2 rRNA from pooled 30 needle and 30 soil amplicon samples resulted in 330,964 reads. Quality filtering showed that 195,808 (59.2%) were of high quality, and 135,156 (40.8%) were of low quality, which were excluded from further analyses. Clustering of high-quality reads showed the presence of 2429 non-singleton contigs at 98% similarity representing different taxa. In addition, there were 3298 singletons, which were excluded. Taxonomic identification showed that among all non-singleton taxa, 1909 (78.6%) were fungal (all non-singleton fungal taxa are in Table S1) and 520 (21.4%) were non-fungal, which were excluded. Rarefaction analysis showed that fungal taxa detected in both needles and soil from respective managed and unmanaged stands vs. the number of sequences did not reach the asymptote (Figure 2). The detected fungi were 59.3% Ascomycota, 37.7% Basidiomycota, 2.6% Mucoromycota, 0.3% Chytridiomycota and 0.1% Glomeromycota.

**Figure 3:**
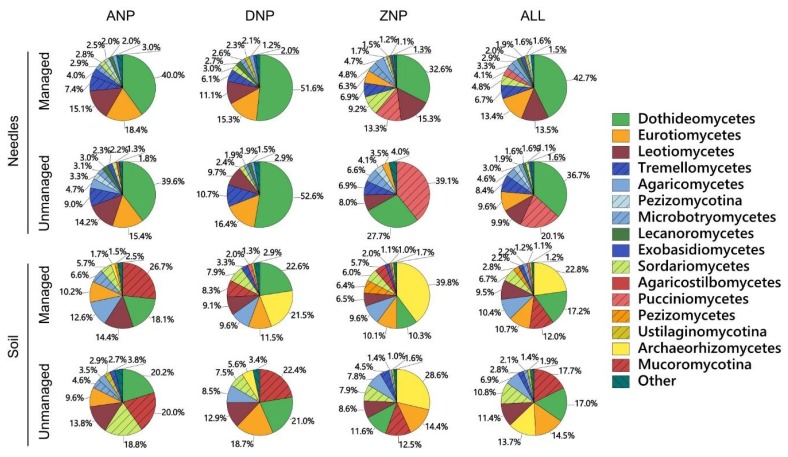
The relative abundance of different fungal classes in living needles and soil from managed and unmanaged *Pinus sylvestris* forest stands at the Aukštaitija National Park (ANP), Dzūkija National Park (DNP) and Žemaitija National Park (ZNP). In ALL, data from different sites is combined.

Analysis of pooled needle data showed that the absolute richness of fungal taxa was marginally higher in managed (1036 taxa out of 54,584 sequences) than in unmanaged stands (1029 out of 60,270) and that 804 taxa were shared between both types of stands. Similarly, soil data showed that the absolute richness of fungal taxa was marginally higher in managed (872 taxa out of 24,873 sequences) than in unmanaged stands (855 out of 25,143) and that 541 taxa were shared between both types of stands. If the same number of sequences had been taken from each type of samples, the difference in chi-squared test was still not significant (*p* > 0.05). Information on the 20 most common fungal taxa in needles and soil representing 60.0% and 58.1% of all fungal sequences in each dataset is in Table 3 and Table 4, all respectively. The most common fungi in needles were *Coleosporium campanulae* (12.4% of all fungal sequences), Unidentified sp. 3980_1 (12.4%), Unidentified sp. 3980_4 (4.1%) and *Sydowia polyspora* (3.1%) (Table 3). The most common fungi in soil were Unidentified sp. 3980_21 (8.6%), *Umbelopsis nana* (8.2%), *Archaeorhizomyces* sp. 3980_5 (8.1%) and *Penicillium spinulosum* (6.3%) (Table 4). Consequently, fungi from the class Dothideomycetes dominated the fungal community in needles and Archaeorhizomycetes in soil (Figure 3).

**Figure 4:**
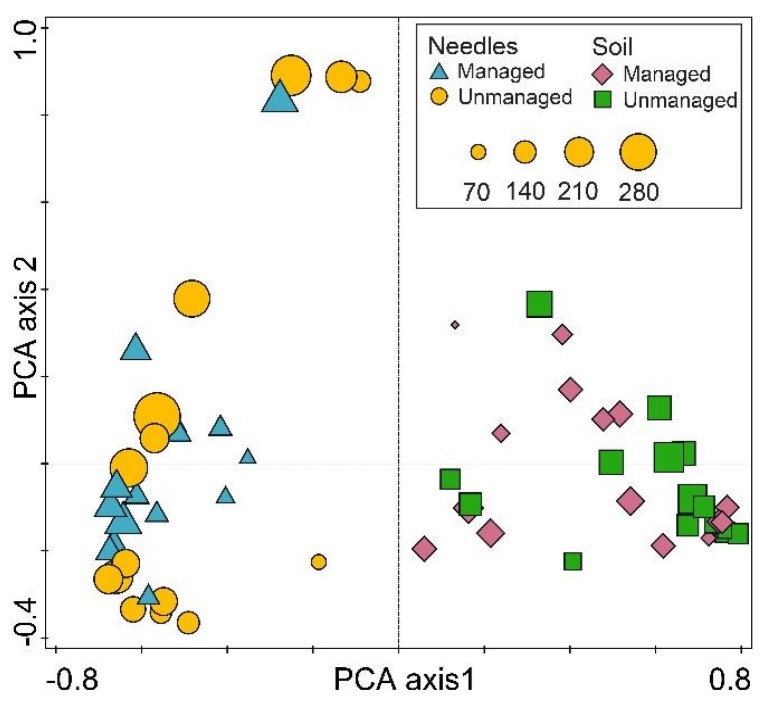
Ordination diagram based on principal component analysis (PCA) of fungal communities in living needles and soil from managed and unmanaged *Pinus sylvestris* forest stands from three national parks in Lithuania. Each point in the diagram represents a single site, and the size of the point reflects the richness of fungal taxa.

Conoco analysis has shown that the response data were compositional and had a gradient 3.9 SD units long, indicating that a linear method, i.e., principal component analysis (PCA), is appropriate. PCA of fungal communities explained 30.0% variation on Axis 1, 8.3% on Axis 2 and 0.05% on Axis 3. PCA showed that within each respective substrate (needles or soil), fungal communities from managed and unmanaged stands were largely intermingled (Figure 4). MANOVA showed that fungal communities from managed *vs*. unmanaged stands did not differ significantly both in needles (*p* > 0.22) and in the soil (*p* > 0.84). By contrast, fungal communities in needles *vs*. soil (managed and unmanaged stands combined) were separated on Axis 1 (Figure 4), and this separation was statistically significant (*p* < 0.0001). In needles, the Shannon diversity index was 3.2–4.3 in managed stands and 1.5–4.4 in unmanaged stands (Table 2). In soil, it was 0.7–4.3 in managed stands and 2.5–4.2 in unmanaged stands (Table 2). Comparison by Mann-Whitney test showed that the Shannon diversity index was significantly higher in needles of managed stands than in unmanaged stands (*p* < 0.036), but there was no significant difference when the corresponding comparison was done for the soil (*p* > 0.51). The Sorensen similarity index of fungal communities between managed and unmanaged stands was 0.77 in needles and 0.63 in the soil.

## 4. Discussion

The results demonstrated that managed and unmanaged *P. sylvestris* stands support similar diversity and composition of fungal communities associated with living needles and soil under the north temperate forest zone conditions (Figure 2, Figure 3 and Figure 4). In support, Parlade et al. [42] have shown that, in *P. sylvestris* stands, a different intensity of forest management has little effect on the overall diversity of soil fungi. The diversity and composition of fungal communities may also depend on the forest structure, i.e., the age and composition of the tree species [23,26], which together with other factors may determine the habitat heterogeneity [4]. As both managed and unmanaged stands within each national park were in the same geographical area and with similar environmental conditions, it appears that the management abandonment had little effect on the stand structure, which remained largely unchanged and resembled managed forests (Table 1). The possibility should not be excluded that this has also contributed to the observed similarities in fungal communities. In comparison, near-natural forests possess higher structural complexity compared to managed stands [43]. The results indicate that changes in fungal communities and the biodiversity recovery can be slow and generally undetectable after 25–34 years since forest management has ceased. Meta-analysis of species richness in Europe have shown that in the first 20 years, species richness can be higher in managed than in unmanaged forests, while the older management abandonment may lead to higher species richness in unmanaged forests [5]. Nevertheless, the overall richness of fungal taxa was high in both managed and unmanaged forest stands of each national park (Table 2), showing that managed forests can also be an important habitat for fungi associated with living needles and soil. As managed forests constitute the majority of forest habitats in Europe, their role in supporting certain groups of fungi should not be underestimated. 

In the present study, the detected diversity of fungal taxa was high and comparable to similar studies on the phyllosphere and soil fungi [17,30,42]. Despite that, the rarefaction curves did not reach the asymptote, indicating that a higher richness of fungal taxa could be detected by deeper sequencing. The fungal community structure was similar in needles from managed and unmanaged stands as compared between corresponding sites of each national park, and when these sites were combined together (Figure 3). An exception was the class of Pucciniomycetes, which was less common in managed than in unmanaged stands (4.1% *vs*. 20.1%, *p* < 0.0001, all sites combined). The Pucciniomycetes class is species-rich and includes many important plant pathogens known as rust fungi [44]. Their higher relative abundance in unmanaged stands is unclear, but may have a negative impact on the health of these forest stands. Although the fungal community structure in soil was also similar between managed and unmanaged stands of each national park (Figure 3), forest management had a positive effect on the relative abundance of the class Archaeorhizomycetes (22.8% *vs*. 13.7%, *p* < 0.0001, all sites combined), which includes a recently described ubiquitous soil fungi with a largely unknown function [45,46]. In support, a high similarity of fungal communities within needle and soil samples was also demonstrated by high values of the Sorensen similarity index. Furthermore, PCA and MANOVA provided evidence that fungal communities were largely specific to each particular substrate (needles or soil) and generally unaffected by the type of forest management (Figure 4). 

*Coleosporium campanulae* dominated fungal communities in needles (Table 3) with a significantly lower relative abundance in managed than in unmanaged stands (4.1% *vs*. 20.0%, *p* < 0.0001). Fungi from the genus *Coleosporium* include pathogens on two-needle pines (*Pinus* spp.) that are mainly distributed in the northern hemisphere [47]. Although this group of fungi has been shown to cause moderate damage in *P. sylvestris* plantations, their response to changes in forest management is generally unknown [48]. *Sydowia polyspora* was also among the most common fungi in needles, but with a similar relative abundance in managed and unmanaged stands (Table 3). *Sydowia polyspora* can be latent in plant tissues [49], but it was also reported in association with current-season needle necrosis [30,50]. Different bark beetles were shown to be vectors for *S. polyspora*, which can rapidly spread and colonise trees following beetle attacks [51,52]. In addition, *S. polyspora* might also benefit from the forest damage caused by other pathogens [53]. As in the present study, the collected needles were healthy-looking, the establishment of *S. polyspora* was probably latent. Interestingly, a number of dominant fungal taxa remained unidentified (Table 3), thereby limiting our understanding about their importance and functional roles. 

Similarly, the most common fungus detected in soil samples (Unidentified sp. 3980_21) remained unidentified (Table 4). This fungus appears to be distantly related to Archaeorhizomycetes, i.e., a class of fungi that taxonomy, ecology and function yet to be resolved. Unidentified sp. 3980_21 and another dominant fungus *Archaeorhizomyces* sp. 3980_5 have shown a higher relative abundance in managed than in unmanaged stands (12.1% *vs*. 5.2%, *p* < 0.0001 and 10.1% *vs*. 6.1%, *p* < 0.0001, respectively), suggesting that forest management can favour some taxa within Archaeorhizomycetes. It appears that these fungi are relatively common and diverse in this geographical area, including *Archaeorhizomyces borealis* [45]. Fungi in this class are strongly associated with soil environments containing plant roots. However, experimental analyses suggest that interactions with roots are neither mycorrhizal nor pathogenic. Instead, species in the Archaeorhizomycetes may exist along a continuum from root endophytic to free-living saprophytic life strategies. It is possible that Archaeorhizomycetes are mycoparasitic, but these life strategies have not yet been studied [54]. Among the other dominant soil fungi, the ectomycorrhizal Ascomycete *Wilcoxina rehmii* showed a higher relative abundance in managed than in unmanaged stands (2.5% *vs*. 0.03%, *p* < 0.0001), while the entomopathogenic fungus *Beauveria pseudobasiana* showed a higher relative abundance in unmanaged stands (0.2% *vs*. 1.4%, *p <* 0.0001), showing certain specificity of these fungi and the potential response to forest management, including site disturbance. Indeed, *Wilcoxina* spp. was shown to prevail on sites following soil disturbance [55,56,57]. *Beauveria* fungi can be important in controlling insect pests [58]. However, their natural occurrence and abundance in soils may depend on site disturbance as much higher relative abundances were reported in natural forest soils than in recent reforestations or agricultural soils [59].

## 5. Conclusions

Managed and unmanaged *P. sylvestris* stands support similar diversity and composition of fungal communities associated with living needles and soil. Some fungal taxa have shown a strong association with either managed or unmanaged stands, thereby providing valuable insights into their ecology and adaptation mechanisms. 

## Figures and Tables

**Table 1:** Characteristics of investigated *Pinus sylvestris* stands. Information is based on the forest inventory data.

Stand	Position	Age (y)	Mean Height (m)	Mean Diameter (cm)	Stocking Level	Forest Site Type *	Forest Vegetation Type **	Tree Species Composition ***
Aukštaitija National Park (ANP)								
Managed	55°13′26″ N, 25°50′49″ E	37	14.8	18	0.7	Nbl	vm	60% P, 30% S, 10% B
	55°13′17″ N, 25°50′51″ E	37	14.8	18	0.7	Nbl	vm	60% P, 30% S, 10% B
	55°13′12″ N, 25°51′26″ E	37	16	18	0.8	Nbl	vm	70% P, 40% S, 20% B
	55°12′51″ N, 25°52′14″ E	34	17.6	18	0.8	Nbl	vm	60% P, 20% S, 20% B
	55°12′44″ N, 25°52′26″ E	107	27.6	37	0.6	Nbl	vm	100% P
Unmanaged	55°13′12″ N, 25°51′08″ E	27	18	19	0.6	Nbl	vm	70% P, 40% B, 20%S
	55°13′11″ N, 25°51′41″ E	67	25.6	28	0.8	Nbl	vm	70% P, 30% S
	55°12′55″ N, 25°51′59″ E	77	25.2	28	0.7	Nbl	vm	80% P, 20% B
	55°12′51″ N, 25°52′07″ E	67	25.2	28	0.7	Nbl	vm	90% P, 10% B
	55°12′50″ N, 25°52′22″ E	34	15.2	16	0.8	Nbl	vm	50% P, 30% B, 20% S
Dzūkija National Park (DNP)								
Managed	54°04′36″ N, 24°19′16″ E	106	24.5	37	0.7	Nal	cl	100% P
	54°04′11″ N, 24°19′34″ E	56	19.5	20	0.9	Nal	cl	80% P, 20% B
	54°04′10″ N, 24°20′30″ E	96	25.6	35	0.7	Nbl	vm	100% P
	54°04′08″ N, 24°20′56″ E	111	25.4	35	0.7	Nal	cl	100% P
	54°03′53″ N, 24°21′45″ E	116	24.4	37	0.6	Nal	cl	100% P
Unmanaged	54°05′29″ N, 24°18′51″ E	61	23.5	26	0.7	Nal	cl	100% P
	54°05′23″ N, 24°18′53″ E	101	26.6	33	0.6	Nal	cl	100% P
	54°05′11″ N, 24°18′58″ E	36	11.8	14	0.9	Nal	cl	80% P, 20% B
	54°04′58″ N, 24°18′58″ E	51	19.7	20	0.8	Nal	cl	80% P, 20% B
	54°04′47″ N, 24°19′04″ E	81	24.9	32	0.8	Nal	cl	100% P
Žemaitija National Park (ZNP)								
Managed	56°02′09″ N, 21°54′45″ E	23	8.8	9	0.7	Ncl	ox	50% P, 30% S, 20% B
	56°02′01″ N, 21°55′04″ E	29	14	13	0.6	Nbl	vm	80% P, 20% S
	56°02′11″ N, 21°55′23″ E	57	23.5	25	0.9	Ncl	ox	100% P
	56°02′35″ N, 21°56′29″ E	67	27.3	29	0.7	Ncl	ox	40% P, 40% S, 20% B
	56°02′42″ N, 21°57′00″ E	32	18.9	19	0.8	Ncl	ox	50% P, 30% S, 20% B
Unmanaged	56°01′22″ N, 21°54′44″ E	67	27.3	29	0.6	Ncl	ox	60% P, 30% S, 10% B
	56°01′35″ N, 21°54′20″ E	52	20.6	25	0.6	Ncl	ox	40% P, 40% S, 20% B
	56°01′24″ N, 21°54′07″ E	52	19.6	21	0.7	Ncl	ox	60% P, 40% S
	56°00′58″ N, 21°52′54″ E	72	26.3	31	0.8	Ncl	ox	90% P, 10% B
	56°00′48″ N, 21°52′18″ E	132	27.1	36	1.0	Pbn	csps	40% P, 40%, 20% B

* N: Normal humidity, a: very poor fertility, b: poor fertility, c: moderate fertility, l: light soil texture; Pbn: oligotrophic soils of drained peatland [34]. ** vm: *vaccinio-myrtilliosum*, cl: *cladoniosum*, ox: *oxalidosum*, csps: *carico-sphagnosum* [35]. *** P: *Pinus sylvestris*, S: *Picea abies*, B: *Betula pendula*. In each stand, tree species composition is based on the volume.

**Table 2:** Sampled needles and soil of *Pinus sylvestris* in managed and unmanaged forest stands, generated high-quality ITS2 rRNA fungal sequences, and detected diversity of fungal taxa.

Stand	Needles				Soil			
	No. of Needle Samples/Amplicon Pools	No. of Sequences	No. of Fungal Taxa	Shannon Diversity Index	No. of Soil Samples/Pools	No. of Sequences	No. of Fungal Taxa	Shannon Diversity Index
Aukštaitija National Park (ANP)								
Managed	5/1	1708	202	4.2	3/1	486	77	3.2
	5/1	3289	284	3.9	3/1	1875	155	3.5
	5/1	1668	183	3.8	3/1	1269	160	4.0
	5/1	4385	318	4.3	3/1	2	2	0.7
	5/1	3496	286	3.8	3/1	2637	144	3.5
All Managed	25/5	14,546	598		15/5	6269	312	
Unmanaged	5/1	5819	378	4.4	3/1	2866	200	3.7
	5/1	3434	196	3.0	3/1	430	114	3.9
	5/1	1697	170	3.5	3/1	718	140	3.9
	5/1	209	71	3.4	3/1	921	60	2.6
	5/1	4106	292	4.1	3/1	1919	133	3.4
All Unmanaged	25/5	15,265	595		15/5	6854	399	
All ANP	50/10	29,811	799		30/10	13,123	525	
Dzūkija National Park (DNP)								
Managed	5/1	5811	240	3.3	3/1	1138	199	4.1
	5/1	4798	276	3.6	3/1	2636	158	3.7
	5/1	8802	341	3.6	3/1	4104	199	2.9
	5/1	2460	174	3.3	3/1	1464	216	4.3
	5/1	1536	134	3.2	3/1	681	165	4.3
All Managed	25/5	23,407	553		15/5	10,023	519	
Unmanaged	5/1	1388	135	3.6	3/1	306	90	3.7
	5/1	2963	194	2.9	3/1	2176	119	2.9
	5/1	3043	207	3.4	3/1	1672	118	3.1
	5/1	1638	141	3.3	3/1	1841	131	3.4
	5/1	5301	247	3.5	3/1	2048	99	2.9
All Unmanaged	25/5	14,333	434		15/5	8043	308	
All DNP	50/10	37,740	662		30/10	18,066	616	
Žemaitija National Park (ZNP)								
Managed	5/1	4977	259	3.6	3/1	1783	174	3.8
	5/1	988	183	4.2	3/1	4366	151	2.3
	5/1	666	106	3.4	3/1	1256	120	2.3
	5/1	6269	339	3.7	3/1	210	98	4.2
	5/1	3731	267	3.7	3/1	966	161	4.2
All Managed	25/5	16,631	638		15/5	8581	450	
Unmanaged	5/1	4058	278	2.8	3/1	2425	151	2.5
	5/1	5214	313	4.1	3/1	1050	173	4.2
	5/1	5751	135	1.5	3/1	887	159	4.2
	5/1	7409	208	2.9	3/1	2,952	212	3.9
	5/1	8240	229	2.9	3/1	2,934	151	2.9
All Unmanaged	25/5	30,672	648		15/5	10,248	517	
All ZNP	50/10	47,303	870		30/10	18,829	741	
All	150/30	114,854	1261		90/30	50,018	1186	

**Table 3:** Relative abundance of the 20 most common fungal taxa sequenced from needles of *Pinus sylvestris* from managed and unmanaged forest stands. Data from different sites is combined.

Taxon	Phylum	Class	GenBank Reference	Similarity, % *	Managed, %	Unmanaged, %	All, %
*Coleosporium campanulae*	Basidiomycota	Pucciniomycetes	KY810468	322/322 (100%)	4.1	20.0	12.4
Unidentified sp. 3980_1	Ascomycota	Dothideomycetes	KP897304	244/244 (100%)	14.2	10.8	12.4
Unidentified sp. 3980_4	Ascomycota	Dothideomycetes	KP891553	259/259 (100%)	3.8	4.5	4.1
*Sydowia polyspora*	Ascomycota	Dothideomycetes	MG888613	256/256 (100%)	3.1	3.0	3.1
Unidentified sp. 3980_13	Ascomycota	Eurotiomycetes	MG827663	262/262 (100%)	3.8	2.1	2.9
Unidentified sp. 3980_3	Basidiomycota	Tremellomycetes	KU687386	302/307 (98%)	3.1	2.5	2.8
*Phaeococcomyces eucalypti*	Ascomycota	Eurotiomycetes	NR_120226	246/248 (99%)	2.6	2.0	2.3
Unidentified sp. 3980_10	Ascomycota	Dothideomycetes	MG827778	258/258 (100%)	2.8	1.8	2.3
*Microsphaeropsis olivacea*	Ascomycota	Dothideomycetes	MH871969	249/249 (100%)	2.1	1.9	2.0
*Epithamnolia xanthoriae*	Ascomycota	Leotiomycetes	KY814539	234/238 (98%)	2.4	1.6	2.0
*Cladosporium cladosporioides*	Ascomycota	Dothideomycetes	MH042811	243/243 (100%)	2.1	1.5	1.8
Unidentified sp. 3980_33	Ascomycota	Leotiomycetes	KP897394	223/258 (86%)	2.0	1.1	1.6
Unidentified sp. 3980_37	Ascomycota	Dothideomycetes	KP897394	236/259 (91%)	2.5	0.6	1.5
*Curvibasidium cygneicollum*	Basidiomycota	Microbotryomycetes	KY102972	310/310 (100%)	1.1	1.7	1.4
Unidentified sp. 3980_30	Ascomycota	Leotiomycetes	KY742593	242/242 (100%)	1.9	0.9	1.4
*Vishniacozyma victoriae*	Basidiomycota	Tremellomycetes	LC085209	234/234 (100%)	0.6	2.0	1.3
Unidentified sp. 3980_25	Ascomycota	Eurotiomycetes	KP891398	255/255 (100%)	1.6	1.0	1.3
*Seimatosporium lichenicola*	Ascomycota	Sordariomycetes	JF320818	247/248 (99%)	2.4	0.2	1.2
*Heterotruncatella spartii*	Ascomycota	Sordariomycetes	MK012418	245/245 (100%)	1.4	0.9	1.1
*Lophodermium pinastri*	Ascomycota	Leotiomycetes	MH856647	239/239 (100%)	1.6	0.6	1.1
Total of 20 taxa					59.3	60.6	60.0

* Similarity column shows base pairs compared between the query sequence and the reference sequence at NCBI databases, and the percentage of sequence similarity in the parenthesis.

**Table 4:** Relative abundance of the 20 most common fungal taxa sequenced from soil from managed and unmanaged stands of *Pinus sylvestris*. Data from different sites is combined.

Taxon	Phylum	Class	GenBank Reference	Similarity, % *	Managed, %	Unmanaged, %	All, %
Unidentified sp. 3980_21	Ascomycota	Archaeorhizomycetes	KC965182	219/219 (100%)	12.1	5.2	8.6
*Umbelopsis nana*	Mucoromycota	Mucoromycotina	MH857049	293/293 (100%)	7.4	8.9	8.2
*Archaeorhizomyces* sp. 3980_5	Ascomycota	Archaeorhizomycetes	MH248043	207/207 (100%)	10.1	6.1	8.1
*Penicillium spinulosum*	Ascomycota	Eurotiomycetes	MK131675	251/251 (100%)	3.8	8.7	6.3
*Oidiodendron chlamydosporicum*	Ascomycota	Dothideomycetes	MG597466	235/235 (100%)	4.4	6.0	5.2
*Oidiodendron echinulatum*	Ascomycota	Dothideomycetes	MG597467	236/236 (100%)	2.0	2.8	2.4
*Sagenomella verticillata*	Ascomycota	Eurotiomycetes	MH860215	263/263 (100%)	2.8	1.7	2.3
Unidentified sp. 3980_28	Mucoromycota	Mucoromycotina	HQ022209	299/299 (100%)	0.9	3.6	2.2
*Tolypocladium geodes*	Ascomycota	Sordariomycetes	MH864065	248/248 (100%)	0.5	3.6	2.0
*Pseudogymnoascus roseus*	Ascomycota	Dothideomycetes	MH865208	241/241 (100%)	1.8	1.5	1.7
*Meliniomyces bicolor*	Ascomycota	Leotiomycetes	MG597461	237/238 (99%)	1.7	1.5	1.6
*Cladosporium cladosporioides*	Ascomycota	Dothideomycetes	MH042811	243/243 (100%)	1.5	1.5	1.5
*Wilcoxina rehmii*	Ascomycota	Pezizomycetes	MF926519	253/254 (99%)	2.5	0.03	1.2
Unidentified sp. 3980_60	Basidiomycota	Microbotryomycetes	HQ021811	320/320 (100%)	1.4	0.7	1.1
Unidentified sp. 3980_64	Ascomycota	Sordariomycetes	KJ826970	317/322 (98%)	1.2	0.9	1.0
*Malassezia restricta*	Basidiomycota	Ustilaginomycotina	CP030254	368/369 (99%)	0.9	1.1	1.0
*Pseudeurotium* sp. 3980_68	Ascomycota	Dothideomycetes	MF692976	229/241 (95%)	1.8	0.2	1.0
*Hyphodiscus* sp. 3980_71	Ascomycota	Leotiomycetes	NR_155151	235/243 (97%)	0.2	1.7	0.9
*Aspergillus cervinus*	Ascomycota	Eurotiomycetes	MH865525	262/262 (100%)	0.8	1.0	0.9
*Beauveria pseudobassiana*	Ascomycota	Sordariomycetes	MF872419	255/255 (100%)	0.2	1.4	0.8
Total of 20 taxa					58.2	58.0	58.1

* Similarity column shows base pairs compared between the query sequence and the reference sequence at NCBI databases, and the percentage of sequence similarity in the parenthesis.

## Supplementary Materials

The following are available online at https://www.mdpi.com/2076-2607/8/2/259/s1, Table S1: Relative abundance of fungal taxa sequenced from needles and soil of *Pinus sylvestris* from managed and unmanaged forest stands in Lithuania.
