# Supplementary material for: Managed and Unmanaged Pinus sylvestris Forest Stands Harbour Similar Diversity and Composition of the Phyllosphere and Soil Fungi
Source: Microorganisms. 2020 Feb 15;8(2):259. doi: 10.3390/microorganisms8020259 (PMC7074758; doi:10.3390/microorganisms8020259)
Supplement: Supplementary file 1 [file microorganisms-08-00259-s001.pdf]

Table S1. Relative abundance of fungal taxa sequenced from needles and soil of *Pinus sylvestris* from managed and unmanaged forest stands in Lithuania

Fungal taxa	Phylum	GenBank reference	Sequence length	Compared with ref. (bp)	Similarity %	Needles			Soil			All
						Managed	Unmanaged	All Needles	Managed	Unmanaged	All Soil	
Unidentified sp. 3980_1	Ascomycota	KP897304	244	244/244	100	14.184	10.798	12.407	1.057	0.390	0.722	8.862
Coleosporium campanulae	Basidiomycota	KY810468	322	322/322	100	4.085	19.970	12.421	0.868	0.235	0.550	8.820
Unidentified sp. 3980_4	Ascomycota	KP891553	259	259/259	100	3.776	4.483	4.147	0.189	0.083	0.136	2.930
Unidentified sp. 3980_21	Ascomycota	KC965182	219	219/219	100	0.018	0.005	0.011	12.138	5.197	8.649	2.632
Umbelopsis nana	Mucoromycota	MH857049	293	293/293	100	0.009	0.010	0.010	7.438	8.878	8.163	2.483
Archaeorhizomyces sp. 3980_5	Ascomycota	MH248043	207	207/207	100	0.016	0.023	0.020	10.111	6.087	8.089	2.468
Sydowia polyspora	Ascomycota	MG888613	256	256/256	100	3.118	3.010	3.061	0.386	0.139	0.262	2.212
Lophodermium seditiosum	Ascomycota	KY742560	247	247/247	100	0.445	0.591	0.522	0.547	0.668	0.608	0.548
Unidentified sp. 3980_13	Ascomycota	MG827663	262	262/262	100	3.845	2.091	2.925	0.225	0.087	0.156	2.085
Unidentified sp. 3980_3	Basidiomycota	KU687386	308	302/307	98	3.052	2.527	2.777	0.129	0.072	0.100	1.965
Penicillium spinulosum	Ascomycota	MK131675	251	251/251	100	0.009	0.007	0.008	3.791	8.735	6.278	1.910
Cladosporium cladosporioides	Ascomycota	MH042811	243	243/243	100	2.123	1.452	1.771	1.508	1.503	1.505	1.690
Phaeococcomyces eucalypti	Ascomycota	KC005769	251	246/248	99	2.567	2.028	2.284	0.205	0.060	0.132	1.631
Unidentified sp. 3980_10	Ascomycota	MG827778	258	258/258	100	2.847	1.774	2.284	0.169	0.087	0.128	1.630
Microsphaeropsis olivacea	Ascomycota	MH871969	249	249/249	100	2.132	1.915	2.018	0.438	0.811	0.626	1.596
Oidiodendron chlamydosporicum	Ascomycota	MG597466	235	235/235	100	0.015	0.008	0.011	4.426	6.012	5.224	1.593
Epithamnolia xanthoriae	Ascomycota	KY814539	238	234/238	98	2.394	1.575	1.964	0.084	0.060	0.072	1.390
Ramularia hydrangeae-macrophyllae	Ascomycota	NR_145125	236	236/236	100	0.317	0.737	0.537	0.334	0.366	0.350	0.480
Vishniacozyma victoriae	Basidiomycota	LC085209	234	234/234	100	0.586	2.011	1.334	0.458	0.175	0.316	1.025
Unidentified sp. 3980_33	Ascomycota	KP897394	257	225/259	87	2.046	1.137	1.569	0.133	0.032	0.082	1.118
Unidentified sp. 3980_37	Ascomycota	KP897394	257	225/260	88	2.486	0.566	1.478	0.052	0.016	0.034	1.040
Curvibasidium cygneicollum	Basidiomycota	KY102972	310	310/310	100	1.130	1.666	1.411	0.080	0.012	0.046	0.997
Helotiales sp. 3980_30	Ascomycota	KY742593	242	242/242	100	1.922	0.859	1.364	0.080	0.028	0.054	0.967
Unidentified sp. 3980_25	Ascomycota	KP891398	255	255/255	100	1.632	1.039	1.321	0.105	0.087	0.096	0.949
Seimatosporium lichenicola	Ascomycota	JF320818	248	248/248	100	2.383	0.214	1.245	0.125	0.048	0.086	0.893
Malassezia restricta	Basidiomycota	CP030254	369	368/369	99	1.460	0.176	0.786	0.893	1.129	1.012	0.855
Heterotruncatella spartii	Ascomycota	MK012418	245	245/245	100	1.414	0.893	1.141	0.092	0.024	0.058	0.812
Lophodermium pinastri	Ascomycota	MH856647	239	239/239	100	1.588	0.576	1.057	0.088	0.032	0.060	0.755
Oidiodendron echinulatum	Ascomycota	MG597467	236	236/236	100	0.007	0.002	0.004	2.046	2.759	2.405	0.733
Unidentified sp. 3980_42	Ascomycota	FR682200	241	240/241	99	1.174	0.831	0.994	0.048	0.032	0.040	0.705
Unidentified sp. 3980_35	Ascomycota	KP891113	268	268/268	100	0.200	1.664	0.968	0.036	0.016	0.026	0.682
Sagenomella verticillata	Ascomycota	MH860215	263	263/263	100	-	0.002	0.001	2.842	1.670	2.253	0.684
Unidentified sp. 3980_28	Chytridiomycota	HQ022209	299	298/299	99	0.007	-	0.003	0.880	3.582	2.239	0.682
Unidentified sp. 3980_43	Ascomycota	FR682155	249	248/249	99	1.055	0.715	0.877	0.080	0.020	0.050	0.626
Tolypocladium geodes	Ascomycota	MH864065	248	248/248	100	0.002	0.003	0.003	0.454	3.614	2.043	0.622
Phaeomoniella pinifoliorum	Ascomycota	MH862974	262	262/262	100	0.746	0.813	0.781	0.096	0.004	0.050	0.559
Unidentified sp. 3980_40	Ascomycota	MG827526	251	251/251	100	0.062	1.419	0.774	0.056	0.008	0.032	0.549
Tremellales sp. 3980_51	Basidiomycota	KX403322	239	196/201	98	0.462	0.939	0.712	0.064	0.044	0.054	0.513
Pseudogymnoascus roseus	Ascomycota	MH865208	241	241/241	100	-	-	-	1.797	1.519	1.657	0.503
Unidentified sp. 3980_52	Ascomycota	AM901716	239	239/239	100	0.746	0.645	0.693	0.060	0.004	0.032	0.493
Meliniomyces bicolor	Ascomycota	MG597461	238	237/238	99	0.002	0.003	0.003	1.705	1.515	1.609	0.490
Unidentified sp. 3980_54	Ascomycota	MF569380	243	242/243	99	0.284	0.974	0.646	0.040	0.020	0.030	0.459

Unidentified sp. 3980_55	Ascomycota	KP897486	247	247/248	99	0.557	0.713	0.639	0.048	0.012	0.030	0.454
Exobasidium maculosum	Basidiomycota	KR262418	288	288/288	100	0.678	0.566	0.619	0.052	0.016	0.034	0.442
Unidentified sp. 3980_67	Ascomycota	KP897366	243	243/243	100	1.024	0.158	0.569	0.032	0.016	0.024	0.404
Unidentified sp. 3980_56	Ascomycota	KM494260	257	257/257	100	0.874	0.305	0.576	0.004	0.020	0.012	0.405
Unidentified sp. 3980_74	Ascomycota	MG827487	264	264/264	100	0.465	0.616	0.544	0.032	0.024	0.028	0.388
Wilcoxina rehmii	Ascomycota	MF926519	254	253/254	99	-	-	-	2.481	0.028	1.248	0.378
Setophoma sp. 3980_63	Ascomycota	KJ869141	251	243/252	96	0.004	0.942	0.496	0.032	0.028	0.030	0.355
Unidentified sp. 3980_60	Basidiomycota	HQ021811	320	319/320	99	0.002	-	0.001	1.415	0.712	1.062	0.323
Unidentified sp. 3980_75	Ascomycota	MG828311	243	243/243	100	0.427	0.465	0.447	0.044	0.012	0.028	0.320
Unidentified sp. 3980_61	Basidiomycota	KP892204	317	317/319	99	0.947	0.005	0.453	0.008	0.004	0.006	0.317
Unidentified sp. 3980_57	Basidiomycota	KU062235	298	297/298	99	0.447	0.450	0.448	0.020	0.004	0.012	0.316
Unidentified sp. 3980_64	Ascomycota	KJ826970	322	317/322	98	-	-	-	1.198	0.887	1.042	0.316
Pseudeurotium sp. 3980_68	Ascomycota	MF692976	241	229/241	95	0.005	0.002	0.003	1.845	0.151	0.994	0.304
Unidentified sp. 3980_73	Ascomycota	MG827708	250	250/250	100	0.843	0.037	0.420	0.016	0.004	0.010	0.295
Unidentified sp. 3980_69	Ascomycota	KX195194	240	236/241	98	0.044	0.121	0.084	0.977	0.553	0.764	0.291
Hyphodiscus sp.3980_71	Ascomycota	NR_155151	243	235/243	97	0.002	0.002	0.002	0.181	1.678	0.934	0.284
Rhodosporidiobolus colostri	Basidiomycota	KY104695	301	301/301	100	0.229	0.531	0.387	0.008	0.004	0.006	0.272
Aspergillus cervinus	Ascomycota	MH865525	262	262/262	100	0.002	-	0.001	0.780	1.010	0.896	0.272
Unidentified sp. 3980_79	Ascomycota	FR682332	262	262/262	100	0.445	0.317	0.378	0.020	0.008	0.014	0.267
Unidentified sp. 3980_72	Basidiomycota	KU061473	281	281/282	99	0.398	0.353	0.374	0.016	0.012	0.014	0.265
Unidentified sp. 3980_78	Ascomycota	MG827622	250	250/250	100	0.207	0.516	0.369	0.024	0.012	0.018	0.263
Unidentified sp. 3980_59	Basidiomycota	AM901976	331	328/334	98	0.093	0.606	0.362	0.008	-	0.004	0.254
Unidentified sp. 3980_70	Ascomycota	MG827482	256	256/256	100	0.465	0.237	0.346	0.008	0.040	0.024	0.248
Unidentified sp. 3980_58	Basidiomycota	KP891940	329	324/329	98	0.399	0.295	0.345	0.012	0.004	0.008	0.243
Unidentified sp. 3980_77	Ascomycota	KP897478	249	220/261	84	0.189	0.484	0.344	0.036	0.008	0.022	0.246
Unidentified sp. 3980_80	Ascomycota	KU062005	262	262/262	100	0.335	0.357	0.347	0.024	0.008	0.016	0.246
Beauveria pseudobassiana	Ascomycota	MF872419	255	255/255	100	0.002	0.003	0.003	0.225	1.356	0.794	0.243
Unidentified sp. 3980_90	Ascomycota	KP897445	276	274/276	99	0.379	0.289	0.332	0.008	0.016	0.012	0.235
Saitozyma podzolica	Basidiomycota	KY107260	255	255/255	100	-	-	-	0.635	0.914	0.776	0.235
Unidentified ap. 3980_87	Ascomycota	MG761091	249	249/249	100	0.445	0.224	0.329	0.012	0.016	0.014	0.234
Scoliciosporum umbrinum	Ascomycota	KX133008	244	244/244	100	0.414	0.229	0.317	0.032	0.012	0.022	0.227
Unidentified sp. 3980_82	Ascomycota	KP897378	246	245/246	99	0.255	0.314	0.286	0.012	0.008	0.010	0.202
Exophiala sideris	Ascomycota	HQ452316	270	269/270	99	0.213	0.342	0.280	0.028	0.012	0.020	0.201
Lecanicillium fungicola	Ascomycota	MH864117	239	239/239	100	0.432	0.129	0.273	0.040	0.004	0.022	0.197
Unidentified sp. 3980_101	Ascomycota	FR682205	251	251/251	100	0.255	0.280	0.268	0.028	-	0.014	0.191
Suillus luteus	Basidiomycota	MH855739	331	331/331	100	0.007	0.003	0.005	1.202	0.008	0.602	0.186
Sporidiobolales sp. 3980_83	Basidiomycota	FJ553238	303	303/303	100	-	-	-	0.298	0.922	0.612	0.186
Trichoderma viride	Ascomycota	MK012400	270	270/270	100	-	-	-	0.804	0.421	0.612	0.186
Unidentified sp. 3980_107	Ascomycota	KM494288	242	242/242	100	-	0.003	0.002	0.334	0.867	0.602	0.184
Unidentified sp. 3980_129	Ascomycota	KX223180	242	242/242	100	-	-	-	0.064	1.129	0.600	0.182
Unidentified sp. 3980_99	Ascomycota	KP897336	257	250/257	97	0.308	0.199	0.251	0.024	0.012	0.018	0.180
Unidentified sp. 3980_131	Ascomycota	KJ188605	216	216/216	100	0.004	-	0.002	0.173	0.990	0.584	0.178
Unidentified sp. 3980_96	Basidiomycota	GU366690	293	292/293	99	-	-	-	0.289	0.811	0.552	0.167
Dioszegia crocea	Basidiomycota	KY558368	220	220/220	100	0.057	0.149	0.105	0.056	0.040	0.048	0.088
Unidentified sp. 3980_104	Basidiomycota	KP897203	258	258/258	100	0.267	0.209	0.237	0.008	-	0.004	0.166
Dothideomycetes sp. 3980_113	Ascomycota	KM519368	245	238/245	97	0.366	0.113	0.233	0.020	0.004	0.012	0.166

Unidentified sp. 3980_92	Basidiomycota	MG827537	309	309/309	100	0.209	0.244	0.227	0.004	0.008	0.006	0.160
Eurotiales sp. 3980_139	Ascomycota	AB986437	251	249/251	99	-	0.003	0.002	0.470	0.588	0.530	0.162
Exobasidium arescens	Basidiomycota	FJ896135	294	291/295	99	0.418	0.058	0.229	0.012	-	0.006	0.161
Unidentified sp. 3980_111	Basidiomycota	MG827732	290	290/290	100	0.278	0.178	0.226	0.012	0.004	0.008	0.160
Hypogymnia physodes	Ascomycota	AF141368	247	247/247	100	0.388	0.065	0.219	0.008	0.028	0.018	0.158
Unidentified sp. 3980_85	Basidiomycota	MH636754	329	329/329	100	-	-	-	0.334	0.712	0.524	0.159
Mortierella macrocystis	Mucoromycota	MH859487	330	330/330	100	-	-	-	0.495	0.553	0.524	0.159
Unidentified sp. 3980_122	Ascomycota	KX222655	242	242/242	100	0.004	-	0.002	0.470	0.553	0.512	0.156
Unidentified sp. 3980_132	Ascomycota	KP898168	247	214/255	84	0.293	0.148	0.217	0.008	-	0.004	0.152
Unidentified sp. 3980_105	Ascomycota	MG828267	252	252/252	100	0.332	0.096	0.208	0.012	0.016	0.014	0.149
Unidentified sp. 3980_124	Ascomycota	JQ312972	215	215/215	100	-	-	-	0.197	0.791	0.496	0.150
Umbelopsis isabellina	Mucoromycota	MH864913	289	289/289	100	0.002	-	0.001	0.434	0.533	0.484	0.147
Unidentified sp. 3980_106	Ascomycota	KP891624	290	248/311	80	0.245	0.166	0.204	0.008	0.020	0.014	0.146
Russula sp. 3980_93	Basidiomycota	MF352768	321	321/321	100	-	-	-	0.941	0.024	0.480	0.146
Unidentified sp. 3980_133	Ascomycota	MG827795	235	235/235	100	0.227	0.184	0.205	-	0.012	0.006	0.144
Cyphellophora sessilis	Ascomycota	MH860834	280	280/280	100	0.218	0.183	0.199	0.020	-	0.010	0.142
Unidentified sp. 3980_144	Ascomycota	KM494483	251	251/251	100	0.071	0.299	0.191	0.008	0.004	0.006	0.135
Unidentified sp. 3980_145	Ascomycota	MG827548	268	254/269	94	0.097	0.275	0.191	0.012	-	0.006	0.135
Unidentified sp. 3980_128	Ascomycota	EU057941	249	210/254	83	0.174	0.199	0.187	0.008	0.012	0.010	0.133
Phialocephala sp. 3980_109	Ascomycota	KP276569	238	238/238	100	0.002	-	0.001	0.458	0.414	0.436	0.133
Unidentified sp. 3980_146	Ascomycota	KP891594	256	256/256	100	0.278	0.096	0.183	0.016	0.008	0.012	0.131
Unidentified sp. 3980_102	Chytridiomycota	KR265911	300	291/300	97	-	0.002	0.001	0.173	0.680	0.428	0.130
Unidentified sp. 3980_118	Ascomycota	MG827455	251	251/251	100	0.044	0.307	0.182	0.008	-	0.004	0.128
Unidentified sp. 3980_151	Ascomycota	MG925034	253	253/253	100	0.180	0.151	0.165	0.056	0.012	0.034	0.125
Inocybe sambucina	Basidiomycota	AM882757	307	306/309	99	-	-	-	0.712	0.107	0.408	0.124
Unidentified sp. 3980_159	Ascomycota	KP897381	252	252/252	100	0.163	0.179	0.172	0.004	0.008	0.006	0.121
Unidentified sp. 3980_147	Ascomycota	GU054276	244	240/244	98	0.024	0.297	0.167	0.012	0.012	0.012	0.120
Unidentified sp. 3980_150	Ascomycota	JF705946	256	256/256	100	0.093	0.237	0.169	-	0.008	0.004	0.119
Unidentified sp. 3980_119	Basidiomycota	KP897510	317	317/317	100	0.200	0.138	0.167	0.004	0.004	0.004	0.118
Mortierellales sp. 3980_116	Mucoromycota	MF482807	331	331/331	100	-	-	-	0.382	0.390	0.386	0.117
Mucor moelleri	Mucoromycota	MH857827	271	271/271	100	-	-	-	0.495	0.278	0.386	0.117
Lophiostoma corticola	Ascomycota	KU712227	244	244/244	100	-	-	-	0.056	0.688	0.374	0.113
Unidentified sp. 3980_149	Basidiomycota	KP897715	253	252/254	99	0.187	0.121	0.152	0.012	0.028	0.020	0.112
Unidentified sp. 3980_115	Ascomycota	DQ979616	249	248/250	99	0.185	0.129	0.156	0.020	0.004	0.012	0.112
Unidentified sp. 3980_120	Basidiomycota	DQ054568	294	293/295	99	-	-	-	0.712	0.032	0.370	0.112
Umbelopsis sp. 3980_125	Mucoromycota	KC007257	294	294/294	100	-	-	-	0.269	0.469	0.370	0.112
Unidentified sp. 3980_153	Ascomycota	KT219333	240	240/240	100	0.148	0.161	0.155	0.020	0.008	0.014	0.112
Unidentified sp. 3980_134	Basidiomycota	MG827448	300	300/300	100	0.130	0.128	0.129	0.068	0.056	0.062	0.109
Unidentified sp. 3980_138	Ascomycota	GQ999538	284	283/284	99	0.240	0.080	0.156	0.004	-	0.002	0.109
Bannozyma sp. 3980_142	Basidiomycota	KY558343	301	301/302	99	0.156	0.148	0.151	0.008	0.008	0.008	0.108
Unidentified sp. 3980_165	Ascomycota	KM493631	249	227/251	90	0.132	0.176	0.155	0.008	-	0.004	0.109
Tremellales sp. 3980_140	Basidiomycota	KR857011	275	275/275	100	0.126	0.174	0.151	0.008	0.004	0.006	0.107
Unidentified sp. 3980_112	Basidiomycota	AF477002	304	304/304	100	-	-	-	0.080	0.624	0.354	0.107
Strasseria geniculata	Ascomycota	MH860198	239	239/239	100	0.249	0.063	0.151	0.008	0.004	0.006	0.107
Unidentified sp. 3980_155	Ascomycota	LT934540	242	222/249	89	0.170	0.124	0.146	0.020	0.012	0.016	0.107
Unidentified sp. 3980_127	Ascomycota	MG827939	237	237/237	100	0.123	0.169	0.147	0.004	-	0.002	0.103

Lophodermium conigenum	Ascomycota	KY742578	245	245/245	100	0.095	0.183	0.141	0.008	-	0.004	0.099
Unidentified sp. 3980_204	Ascomycota	KM374351	239	238/239	99	-	-	-	0.414	0.191	0.302	0.092
Unidentified sp. 3980_196	Ascomycota	MG827532	256	256/256	100	0.203	0.056	0.126	-	0.016	0.008	0.090
Sesquicillium microsporum	Ascomycota	MH859708	250	250/250	100	-	-	-	0.201	0.394	0.298	0.090
Unidentified sp. 3980_180	Ascomycota	MG827697	244	229/246	93	0.134	0.121	0.127	0.008	0.004	0.006	0.090
Unidentified sp. 3980_141	Basidiomycota	KU188743	310	310/310	100	-	-	-	-	0.580	0.292	0.089
Unidentified sp. 3980_162	Ascomycota	KR266244	238	238/238	100	0.002	0.002	0.002	0.482	0.080	0.280	0.086
Aspergillus inflatus	Ascomycota	MH859900	258	258/258	100	-	-	-	0.370	0.199	0.284	0.086
Unidentified sp. 3980_163	Basidiomycota	MG828182	294	294/294	100	0.011	0.212	0.117	0.008	0.004	0.006	0.083
Cortinarius sp. 3980_181	Basidiomycota	JQ711860	279	278/279	99	0.015	0.030	0.023	0.269	0.175	0.222	0.083
Unidentified sp. 3980_192	Ascomycota	KU188564	238	238/238	100	-	-	-	0.036	0.509	0.274	0.083
Unidentified sp. 3980_136	Basidiomycota	KP897246	288	285/288	99	0.198	0.045	0.118	-	-	-	0.082
Dothideomycetes sp. 3980_169	Ascomycota	KX909208	251	251/251	100	0.218	0.018	0.113	0.016	0.008	0.012	0.082
Unidentified sp. 3980_160	Basidiomycota	JN032555	299	299/299	100	0.108	0.073	0.090	0.113	0.016	0.064	0.082
Oidiodendron flavum	Ascomycota	AF062792	235	235/235	100	-	-	-	0.161	0.378	0.270	0.082
Unidentified sp. 3980_217	Ascomycota	KP898071	240	238/241	99	0.156	0.076	0.114	0.004	0.004	0.004	0.081
Russula sp. 3980_173	Basidiomycota	HM044597	321	321/321	100	-	-	-	0.394	0.135	0.264	0.080
Tremellomycetes sp. 3980_173	Basidiomycota	KR265960	251	251/251	100	0.114	0.110	0.111	0.008	0.004	0.006	0.079
Unidentified sp. 3980_225	Ascomycota	KX193511	243	243/243	100	-	-	-	0.370	0.151	0.260	0.079
Unidentified sp. 3980_197	Ascomycota	EU516957	247	247/247	100	0.057	0.159	0.111	-	0.004	0.002	0.078
Unidentified sp. 3980_184	Ascomycota	MG828352	249	229/254	90	0.194	0.030	0.108	0.008	0.004	0.006	0.077
Unidentified sp. 3980_228	Ascomycota	MF571509	246	231/247	94	-	0.211	0.111	-	-	-	0.077
Unidentified sp. 3980_172	Basidiomycota	KX886193	288	288/288	100	0.002	0.002	0.002	0.225	0.270	0.248	0.076
Alternaria conjuncta	Ascomycota	MH861940	253	253/253	100	0.059	0.118	0.090	0.024	0.064	0.044	0.076
Tremellales sp. 3980_164	Basidiomycota	KX403023	274	197/200	99	0.046	0.161	0.106	-	-	-	0.074
Unidentified sp. 3980_189	Ascomycota	MG827441	250	250/250	100	0.143	0.068	0.104	0.008	-	0.004	0.073
Unidentified sp. 3980_177	Ascomycota	MG827606	257	257/257	100	0.079	0.124	0.103	-	0.008	0.004	0.073
Exobasidium vaccinii	Basidiomycota	MH857572	300	300/300	100	0.169	0.041	0.102	0.004	0.004	0.004	0.072
Hypholoma sp. 3980_157	Basidiomycota	MF615084	304	304/304	100	0.121	0.080	0.099	-	0.012	0.006	0.071
Sordariomycetes sp. 3980_247	Ascomycota	MH451667	246	246/246	100	-	-	-	0.402	0.072	0.236	0.072
Unidentified sp. 3980_168	Mucoromycota	KX221634	291	291/291	100	-	0.002	0.001	0.008	0.449	0.230	0.070
Unidentified sp. 3980_194	Ascomycota	MG827697	245	245/245	100	0.134	0.070	0.100	0.004	-	0.002	0.070
Hormonema carpetanum	Ascomycota	KU516485	256	256/256	100	0.178	0.023	0.097	0.004	0.012	0.008	0.070
Cadophora sp. 3980_221	Ascomycota	JX243949	241	240/241	99	0.022	0.015	0.018	0.149	0.223	0.186	0.069
Tricholoma saponaceum	Basidiomycota	HM196023	295	295/295	100	-	-	-	0.438	0.008	0.222	0.067
Unidentified sp. 3980_235	Ascomycota	KU061959	242	235/243	97	0.088	0.095	0.091	0.012	0.004	0.008	0.066
Unidentified sp. 3980_246	Ascomycota	KY633588	239	216/240	90	0.159	0.030	0.091	0.008	0.004	0.006	0.066
Unidentified sp. 3980_187	Basidiomycota	KY104940	317	272/327	83	0.137	0.051	0.092	-	-	-	0.064
Unidentified sp. 3980_223	Ascomycota	KP897577	242	242/242	100	0.099	0.076	0.087	0.020	0.004	0.012	0.064
Unidentified sp. 3980_171	Ascomycota	HM240809	252	252/252	100	0.046	0.126	0.088	-	0.016	0.008	0.064
Unidentified sp. 3980_227	Ascomycota	KF617857	257	257/257	100	-	-	-	0.141	0.278	0.210	0.064
Unidentified sp. 3980_231	Ascomycota	JQ312813	235	234/235	99	-	-	-	0.012	0.406	0.210	0.064
Mortierella verticillata	Mucoromycota	MH860122	333	333/333	100	-	-	-	0.117	0.294	0.206	0.062
Cordyceps militaris	Ascomycota	MH858409	254	254/254	100	0.007	0.002	0.004	0.101	0.290	0.196	0.062
Unidentified sp. 3980_195	Basidiomycota	KP897294	252	252/252	100	0.057	0.118	0.089	0.004	-	0.002	0.062
Unidentified sp. 3980_190	Basidiomycota	KU062818	291	291/291	100	0.093	0.083	0.088	-	0.004	0.002	0.062

Nectria dematiosa	Ascomycota	MH864605	263	263/263	100	0.183	-	0.087	0.008	-	0.004	0.062
Sebacina sp. 3980_201	Basidiomycota	GU189680	299	295/299	99	-	-	-	0.394	-	0.196	0.059
Fusicladium ramoconidii	Ascomycota	MH861516	266	266/268	99	0.130	0.041	0.084	0.004	0.004	0.004	0.059
Unidentified sp. 3980_207	Ascomycota	JN032498	240	240/240	100	0.136	0.030	0.080	0.020	-	0.010	0.059
Unidentified sp. 3980_215	Mucoromycota	KP897509	291	237/301	79	0.095	0.066	0.080	0.008	0.008	0.008	0.058
Unidentified sp. 3980_200	Basidiomycota	KP897812	317	315/318	99	0.077	0.086	0.082	0.004	-	0.002	0.058
Unidentified sp. 3980_186	Ascomycota	KP897260	238	235/242	97	0.066	0.093	0.080	0.004	0.004	0.004	0.057
Hypocreales sp. 3980_208	Ascomycota	MF485431	273	273/273	100	0.055	0.098	0.077	0.016	-	0.008	0.056
Unidentified sp. 3980_264	Ascomycota	LT934540	243	225/250	90	0.156	0.013	0.081	0.004	-	0.002	0.057
Unidentified sp. 3980_306	Ascomycota	MG827508	248	248/248	100	0.093	0.032	0.061	0.068	0.020	0.044	0.056
Unidentified sp. 3980_198	Basidiomycota	KP891312	332	332/332	100	0.139	0.025	0.079	-	0.004	0.002	0.056
Unidentified sp. 3980_344	Ascomycota	KT219769	212	212/212	100	-	-	-	0.113	0.250	0.182	0.055
Mortierella alpina	Mucoromycota	MH859872	344	344/344	100	-	-	-	0.269	0.091	0.180	0.055
Unidentified sp. 3980_220	Ascomycota	MF784612	239	239/239	100	0.004	-	0.002	0.101	0.250	0.176	0.055
Unidentified sp. 3980_241	Mucoromycota	KR265911	299	299/299	100	-	-	-	0.012	0.346	0.180	0.055
Unidentified sp. 3980_185	Mucoromycota	KC818345	323	323/323	100	-	-	-	0.117	0.235	0.176	0.053
Unidentified sp. 3980_236	Ascomycota	KP897314	252	251/252	99	0.154	0.005	0.076	0.004	-	0.002	0.053
Mortierellomycetes sp. 3980_193	Mucoromycota	MH451642	337	337/337	100	-	-	-	0.008	0.334	0.172	0.052
Diversisporales sp. 3980_245	Mucoromycota	MF484167	309	295/309	95	0.002	0.139	0.074	0.004	-	0.002	0.052
Unidentified sp. 3980_254	Ascomycota	MG828183	236	236/236	100	0.064	0.073	0.069	0.016	-	0.008	0.050
Pseudotomentella sp. 3980_214	Basidiomycota	KP403033	316	316/316	100	-	-	-	0.326	-	0.162	0.049
Unidentified sp. 3980_230	Ascomycota	MG828003	244	243/244	99	0.106	0.035	0.069	0.012	-	0.006	0.050
Unidentified sp. 3980_238	Ascomycota	MG827773	273	273/274	99	0.055	0.081	0.069	0.004	0.004	0.004	0.049
Unidentified sp. 3980_244	Ascomycota	KP897656	263	263/263	100	0.060	0.080	0.071	0.004	-	0.002	0.050
Unidentified sp. 3980_218	Basidiomycota	KP897398	301	301/301	100	0.060	0.075	0.068	0.012	-	0.006	0.049
Unidentified sp. 3980_249	Ascomycota	KT219230	294	250/257	97	-	-	-	0.149	0.175	0.162	0.049
Unidentified sp. 3980_276	Ascomycota	KP887887	238	238/238	100	0.027	0.108	0.070	-	-	-	0.049
Unidentified sp. 3980_212	Basidiomycota	KP897444	294	293/296	99	0.084	0.056	0.070	-	-	-	0.049
Unidentified sp. 3980_232	Ascomycota	MG827476	244	243/244	99	0.044	0.088	0.067	0.008	-	0.004	0.048
Mrakia aquatica	Basidiomycota	MK372216	326	326/326	100	0.016	0.113	0.067	-	-	-	0.047
Unidentified sp. 3980_251	Ascomycota	GU054274	246	245/246	99	0.093	0.027	0.058	0.032	0.004	0.018	0.046
Unidentified sp. 3980_261	Ascomycota	KP897277	268	268/268	100	0.048	0.080	0.064	0.004	0.008	0.006	0.047
Dothistroma septosporum	Ascomycota	MH865094	236	236/236	100	0.035	0.091	0.064	-	0.008	0.004	0.046
Unidentified sp. 3980_259	Ascomycota	MG827410	249	249/249	100	0.064	0.040	0.051	0.044	0.024	0.034	0.046
Unidentified sp. 3980_286	Ascomycota	KP889412	242	239/242	99	-	0.123	0.064	0.004	-	0.002	0.045
Unidentified sp. 3980_283	Basidiomycota	KP891040	271	271/271	100	0.029	0.098	0.065	-	-	-	0.045
Oidiodendron sp. 3980_291	Ascomycota	KJ008860	234	234/234	100	-	0.002	0.001	0.117	0.179	0.148	0.045
Unidentified sp. 3980_222	Basidiomycota	KX194561	321	321/321	100	0.035	0.086	0.062	-	0.008	0.004	0.044
Lophodermium piceae	Ascomycota	FR837918	238	238/238	100	0.037	0.080	0.059	0.004	0.008	0.006	0.043
Unidentified sp. 3980_280	Ascomycota	KP891522	256	256/256	100	0.081	0.046	0.063	-	-	-	0.044
Unidentified sp. 3980_309	Ascomycota	LT608050	259	259/259	100	-	-	-	0.028	0.258	0.144	0.044
Tremellales sp. 3980_253	Basidiomycota	MF486673	245	245/245	100	0.018	0.101	0.062	-	-	-	0.043
Unidentified sp. 3980_262	Basidiomycota	KU061975	267	265/269	99	0.108	0.020	0.062	-	-	-	0.043
Pleotrichocladium opacum	Ascomycota	MH859791	244	244/244	100	-	-	-	0.113	0.171	0.142	0.043
Unidentified sp. 3980_284	Ascomycota	AB476466	237	237/237	100	-	-	-	0.105	0.179	0.142	0.043
Mortierella sp. 3980_203	Mucoromycota	KX115819	344	344/344	100	-	-	-	0.261	0.020	0.140	0.042

Unidentified sp. 3980_237	Ascomycota	KY419186	281	243/286	85	0.075	0.045	0.059	0.008	-	0.004	0.042
Unidentified sp. 3980_242	Basidiomycota	KY104942	324	281/331	85	0.011	0.106	0.061	-	-	-	0.042
Unidentified sp. 3980_266	Ascomycota	KM493714	237	237/237	100	0.084	0.033	0.057	0.008	0.004	0.006	0.042
Phaeosphaeria sp. 3980_294	Ascomycota	MH935028	244	244/244	100	0.029	0.086	0.059	0.004	0.004	0.004	0.042
Unidentified sp. 3980_299	Ascomycota	MG827665	259	259/259	100	-	-	-	0.177	0.099	0.138	0.042
Unidentified sp. 3980_332	Ascomycota	MG827660	254	254/254	100	0.064	0.022	0.042	-	0.076	0.038	0.041
Russula aeruginea	Basidiomycota	MG680182	301	301/301	100	-	-	-	0.133	0.135	0.134	0.041
Malassezia sympodialis	Basidiomycota	LT671825	327	327/327	100	0.055	0.013	0.033	0.024	0.091	0.058	0.041
Unidentified sp. 3980_258	Ascomycota	FJ554342	242	242/242	100	0.024	0.050	0.037	0.020	0.072	0.046	0.040
Unidentified sp. 3980_315	Ascomycota	MG827964	244	244/244	100	0.086	0.030	0.057	0.004	-	0.002	0.040
Unidentified sp. 3980_233	Basidiomycota	MG827587	326	326/326	100	0.066	0.041	0.053	0.004	0.004	0.004	0.038
Unidentified sp. 3980_255	Ascomycota	MG827849	273	273/273	100	0.011	0.095	0.055	0.004	-	0.002	0.039
Unidentified sp. 3980_260	Ascomycota	KP891410	255	255/255	100	0.070	0.037	0.052	0.008	0.004	0.006	0.038
Thelephoraceae sp. 3980_267	Basidiomycota	KU727191	314	314/314	100	-	-	-	0.257	-	0.128	0.039
Unidentified sp. 3980_292	Ascomycota	JQ312710	240	240/242	99	-	-	-	-	0.254	0.128	0.039
Monilinia fructigena	Ascomycota	MH862738	239	239/239	100	0.002	-	0.001	0.249	-	0.124	0.038
Unidentified sp. 3980_282	Basidiomycota	KP892376	320	320/320	100	0.086	0.025	0.054	0.004	-	0.002	0.038
Unidentified sp. 3980_279	Ascomycota	MH411692	256	256/256	100	-	-	-	0.121	0.127	0.124	0.038
Heterobasidion annosum	Basidiomycota	MH859050	298	298/298	100	0.029	0.013	0.021	0.125	0.028	0.076	0.038
Unidentified sp. 3980_355	Ascomycota	AY969364	238	226/227	99	-	-	-	-	0.247	0.124	0.038
Unidentified sp. 3980_239	Basidiomycota	KX223212	374	374/374	100	0.024	0.002	0.012	0.121	0.068	0.094	0.037
Udeniomyces pyricola	Basidiomycota	KY105778	333	333/333	100	0.073	0.033	0.052	0.004	-	0.002	0.037
Unidentified sp. 3980_304	Ascomycota	KX223062	241	241/241	100	-	-	-	0.088	0.155	0.122	0.037
Unidentified sp. 3980_263	Basidiomycota	KX220248	279	279/279	100	0.005	0.008	0.007	0.201	0.008	0.104	0.036
Neocucurbitaria populi	Ascomycota	MF795774	249	249/249	100	0.071	0.033	0.051	-	0.004	0.002	0.036
Cortinarius sp. 3980_257	Basidiomycota	HQ021915	284	284/284	100	0.059	0.043	0.050	-	-	-	0.035
Inocybe sp. 3980_278	Basidiomycota	FR852257	300	299/300	99	-	-	-	-	0.231	0.116	0.035
Unidentified sp. 3980_300	Basidiomycota	KU061776	253	253/253	100	0.088	0.013	0.049	0.008	-	0.004	0.035
Unidentified sp. 3980_305	Ascomycota	KX221939	240	240/240	100	0.002	-	0.001	0.129	0.099	0.114	0.035
Unidentified sp. 3980_302	Ascomycota	KJ826762	229	222/230	97	0.046	0.048	0.047	0.004	0.004	0.004	0.034
Unidentified sp. 3980_372	Ascomycota	KM493701	248	248/248	100	0.068	0.030	0.048	-	0.004	0.002	0.034
Unidentified sp. 3980_335	Ascomycota	KP892318	236	236/236	100	0.002	0.090	0.048	0.004	-	0.002	0.034
Unidentified sp. 3980_226	Basidiomycota	EF040837	325	324/325	99	-	-	-	0.076	0.139	0.108	0.033
Unidentified sp. 3980_287	Basidiomycota	KU064199	273	272/274	99	0.057	0.037	0.046	0.004	-	0.002	0.033
Lycoperdon pyriforme	Basidiomycota	MH856649	301	301/301	100	0.024	0.028	0.026	0.004	0.091	0.048	0.033
Unidentified sp. 3980_404	Ascomycota	MG828336	241	241/241	100	0.037	0.051	0.044	0.004	0.004	0.004	0.032
Unidentified sp. 3980_338	Basidiomycota	JQ312881	274	274/274	100	-	-	-	0.072	0.139	0.106	0.032
Unidentified sp. 3980_408	Ascomycota	KX220164	241	239/241	99	-	-	-	0.209	0.004	0.106	0.032
Unidentified sp. 3980_473	Ascomycota	KX221290	236	236/236	100	-	-	-	0.020	0.191	0.106	0.032
fungal endophyte	Ascomycota	KU179276	247	247/247	100	0.002	0.045	0.024	0.016	0.080	0.048	0.032
Unidentified sp. 3980_269	Ascomycota	AF504876	228	223/228	98	-	-	-	0.096	0.107	0.102	0.031
Unidentified sp. 3980_270	Ascomycota	LT608104	241	240/242	99	-	-	-	0.048	0.155	0.102	0.031
Tuberculina maxima	Basidiomycota	AY292437	307	304/307	99	-	0.083	0.044	0.004	-	0.002	0.031
Unidentified sp. 3980_295	Ascomycota	KT198039	297	250/252	99	-	-	-	0.177	0.028	0.102	0.031
Unidentified sp. 3980_303	Ascomycota	MG827548	265	265/265	100	0.026	0.061	0.044	-	-	-	0.031
Unidentified sp. 3980_310	Ascomycota	MG761091	257	217/259	84	0.015	0.071	0.044	-	-	-	0.031

Unidentified sp. 3980_345	Basidiomycota	KP891880	273	273/273	100	0.009	0.075	0.044	-	0.004	0.002	0.031
Unidentified sp. 3980_346	Ascomycota	KP897402	245	244/245	99	0.042	0.043	0.043	-	0.004	0.002	0.030
Kwoniella pini	Basidiomycota	KY558354	285	285/285	100	0.005	0.076	0.043	-	0.008	0.004	0.031
Unidentified sp. 3980_386	Ascomycota	KX222128	243	225/245	92	0.049	0.038	0.044	-	-	-	0.030
Unidentified sp. 3980_274	Basidiomycota	KP898007	332	298/343	87	0.011	0.073	0.044	-	-	-	0.030
Unidentified sp. 3980_290	Ascomycota	KX222736	310	299/315	95	-	-	-	0.201	-	0.100	0.030
Unidentified sp. 3980_316	Ascomycota	AM901749	255	254/255	99	0.057	0.032	0.044	-	-	-	0.030
Unidentified sp. 3980_340	Ascomycota	EF419944	246	242/246	98	0.013	0.065	0.040	0.012	0.004	0.008	0.030
Unidentified sp. 3980_431	Ascomycota	MF569392	241	236/241	98	0.022	0.060	0.042	0.004	-	0.002	0.030
Pleosporales sp. 3980_285	Ascomycota	MH452387	248	247/248	99	0.031	0.043	0.037	0.012	0.008	0.010	0.029
Unidentified sp. 3980_330	Ascomycota	KX220599	255	251/255	98	0.037	0.048	0.043	-	-	-	0.030
Unidentified sp. 3980_333	Basidiomycota	KP897275	322	322/322	100	0.033	0.050	0.042	-	0.004	0.002	0.030
Unidentified sp. 3980_358	Ascomycota	KX222742	241	234/242	97	0.051	0.033	0.042	0.004	-	0.002	0.030
Cortinarius biformis	Basidiomycota	MH784746	279	279/279	100	-	-	-	0.197	-	0.098	0.030
Inocybe lacera	Basidiomycota	MG597389	303	303/303	100	0.029	0.035	0.032	0.048	-	0.024	0.030
Unidentified sp. 3980_433	Ascomycota	KX220128	247	247/247	100	0.002	-	0.001	0.096	0.095	0.096	0.030
Unidentified sp. 3980_460	Ascomycota	KP897810	248	247/249	99	-	-	-	0.141	0.056	0.098	0.030
Unidentified sp. 3980_349	Ascomycota	KP897719	277	277/277	100	0.088	-	0.042	-	-	-	0.029
Unidentified sp. 3980_391	Basidiomycota	KT219406	236	236/236	100	0.044	0.035	0.039	0.004	-	0.002	0.028
Unidentified sp. 3980_275	Ascomycota	KP897257	301	300/301	99	0.081	-	0.038	-	0.008	0.004	0.028
Unidentified sp. 3980_307	Ascomycota	KT244612	253	252/256	98	-	-	-	0.129	0.056	0.092	0.028
Unidentified sp. 3980_342	Ascomycota	KP897598	252	249/253	98	0.053	0.027	0.039	-	0.004	0.002	0.028
Unidentified sp. 3980_361	Ascomycota	MG827954	249	248/249	99	0.079	0.003	0.039	0.004	-	0.002	0.028
Unidentified sp. 3980_374	Ascomycota	KM493417	266	266/266	100	-	-	-	0.016	0.167	0.092	0.028
Meliniomyces sp. 3980_411	Ascomycota	HQ157835	242	242/242	100	-	-	-	0.004	0.179	0.092	0.028
Unidentified sp. 3980_318	Ascomycota	KP891956	250	250/250	100	0.009	0.061	0.037	0.012	-	0.006	0.027
Pithomyces chartarum	Ascomycota	MH860227	248	248/248	100	0.015	0.048	0.032	0.024	0.008	0.016	0.027
Unidentified sp. 3980_379	Basidiomycota	LT608038	250	250/250	100	-	-	-	0.036	0.143	0.090	0.027
Unidentified sp. 3980_436	Ascomycota	KP897538	238	236/240	98	0.049	0.030	0.039	-	-	-	0.027
Unidentified sp. 3980_321	Zoopagomycota	KP892133	296	295/296	99	0.033	0.043	0.038	-	-	-	0.027
Unidentified sp. 3980_323	Ascomycota	KP897243	230	230/230	100	0.062	0.017	0.038	-	-	-	0.027
Unidentified sp. 3980_325	Basidiomycota	KP891645	338	122/154	79	0.038	0.037	0.037	-	-	-	0.026
Unidentified sp. 3980_329	Basidiomycota	KU189097	319	317/319	99	0.027	0.046	0.037	-	-	-	0.026
Unidentified sp. 3980_434	Ascomycota	KJ828062	241	232/242	96	0.048	0.030	0.038	-	-	-	0.027
Unidentified sp. 3980_256	Ascomycota	KC818336	242	242/242	100	0.009	0.015	0.012	0.040	0.076	0.058	0.026
Mortierella sp. 3980_301	Mucoromycota	HQ021782	372	372/372	100	-	-	-	0.012	0.159	0.086	0.026
Unidentified sp. 3980_312	Ascomycota	KM494122	241	238/241	99	0.038	0.033	0.036	0.004	0.004	0.004	0.026
Monoblepharidales sp. 3980_378	Chytridiomycota	MF484372	300	300/300	100	0.033	0.040	0.037	0.004	-	0.002	0.026
Fusarium tricinctum	Ascomycota	MK212907	258	258/258	100	0.020	0.043	0.032	0.008	0.012	0.010	0.025
Oidiodendron periconioides	Ascomycota	MH864342	236	236/236	100	-	0.002	0.001	0.060	0.103	0.082	0.025
Russula decolorans	Basidiomycota	MH979312	359	359/359	100	-	-	-	0.149	0.016	0.082	0.025
Unidentified sp. 3980_343	Basidiomycota	KX222911	258	258/258	100	-	-	-	0.040	0.127	0.084	0.025
Unidentified sp. 3980_362	Ascomycota	DQ309133	258	258/258	100	-	-	-	0.020	0.147	0.084	0.025
Stemphylium globuliferum	Ascomycota	MH399295	254	254/254	100	0.005	0.008	0.007	0.133	0.004	0.068	0.025
Unidentified sp. 3980_450	Basidiomycota	KP889854	251	251/251	100	-	-	-	0.028	0.139	0.084	0.025
Unidentified sp. 3980_281	Basidiomycota	KX515562	300	300/300	100	0.002	0.065	0.035	-	-	-	0.024

Unidentified sp. 3980_288	Basidiomycota	KT242698	334	67/71	94	0.040	0.030	0.035	0.004	-	0.002	0.025
Unidentified sp. 3980_322	Basidiomycota	KP891447	300	193/244	79	0.033	0.035	0.034	-	0.004	0.002	0.024
Unidentified sp. 3980_359	Ascomycota	EU754966	243	243/243	100	-	-	-	0.165	-	0.082	0.025
Unidentified sp. 3980_414	Ascomycota	KP897197	240	240/240	100	0.060	0.013	0.036	-	-	-	0.025
Helotiales sp. 3980_487	Ascomycota	MH451745	241	241/241	100	-	-	-	0.165	-	0.082	0.025
Unidentified sp. 3980_562	Ascomycota	MG207583	243	243/243	100	0.005	0.063	0.036	-	-	-	0.025
Hypholoma sp. 3980_308	Basidiomycota	HM240821	304	304/304	100	0.044	0.025	0.034	0.004	-	0.002	0.024
Unidentified sp. 3980_381	Ascomycota	MH862974	257	245/263	93	0.024	0.045	0.035	-	-	-	0.024
Unidentified sp. 3980_420	Basidiomycota	MG827822	267	267/267	100	0.024	0.043	0.034	-	0.004	0.002	0.024
Sistotrema sp. 3980_324	Basidiomycota	KP814242	300	300/300	100	-	-	-	0.068	0.087	0.078	0.024
Mucor hiemalis	Mucoromycota	KX426958	272	272/272	100	0.057	0.010	0.032	-	0.008	0.004	0.024
Unidentified sp. 3980_400	Cryptomycota	KU062592	264	247/256	96	0.004	0.002	0.003	0.141	0.004	0.072	0.024
Unidentified sp. 3980_511	Ascomycota	MG827702	249	249/249	100	-	-	-	0.048	0.107	0.078	0.024
Lycoperdon perlatum	Basidiomycota	DQ112630	309	309/309	100	0.016	0.033	0.025	0.004	0.032	0.018	0.023
Unidentified sp. 3980_353	Ascomycota	LT608107	240	239/240	99	-	-	-	-	0.151	0.076	0.023
Unidentified sp. 3980_421	Ascomycota	KC588597	231	213/232	92	0.037	0.023	0.030	0.008	0.008	0.008	0.023
Microcyclospora malicola	Ascomycota	MH864047	243	242/243	99	0.057	0.007	0.030	0.008	0.004	0.006	0.023
Crumenulopsis sororia	Ascomycota	KY941133	240	240/240	100	0.009	0.053	0.032	-	0.004	0.002	0.023
Krasilnikovozyma huempii	Basidiomycota	KY103892	334	334/334	100	0.048	0.018	0.032	-	-	-	0.022
Unidentified sp. 3980_393	Ascomycota	KX222977	270	270/270	100	-	-	-	0.149	-	0.074	0.022
Unidentified sp. 3980_424	Ascomycota	MG827445	238	238/238	100	0.037	0.023	0.030	0.008	-	0.004	0.022
Unidentified sp. 3980_337	Basidiomycota	KM493939	337	335/337	99	0.018	0.043	0.031	-	-	-	0.022
Unidentified sp. 3980_341	Basidiomycota	MH452480	340	322/343	94	-	-	-	-	0.143	0.072	0.022
Coprinellus micaceus	Basidiomycota	KU712252	298	298/298	100	0.038	0.025	0.031	-	-	-	0.022
Unidentified sp. 3980_363	Basidiomycota	MG827699	247	247/247	100	0.026	0.028	0.027	0.008	0.008	0.008	0.021
Unidentified sp. 3980_428	Ascomycota	KC588554	242	242/242	100	-	-	-	0.064	0.080	0.072	0.022
Unidentified sp. 3980_438	Ascomycota	KP889419	241	238/241	99	-	-	-	-	0.143	0.072	0.022
Truncatella angustata	Ascomycota	MH860182	246	246/246	100	-	0.027	0.014	0.032	0.048	0.040	0.022
Unidentified sp. 3980_605	Ascomycota	MG828025	240	240/240	100	0.038	0.025	0.031	-	-	-	0.022
Mucor zonatus	Mucoromycota	MH860167	272	272/272	100	-	-	-	0.052	0.087	0.070	0.021
Malassezia globosa	Basidiomycota	KM269150	386	386/386	100	0.018	0.013	0.016	0.008	0.060	0.034	0.021
Unidentified sp. 3980_360	Ascomycota	KF618007	234	231/235	98	-	-	-	0.056	0.083	0.070	0.021
Unidentified sp. 3980_375	Ascomycota	MF570772	242	231/242	95	-	-	-	0.133	0.008	0.070	0.021
Unidentified sp. 3980_377	Basidiomycota	FR682146	259	235/262	90	0.007	0.046	0.028	0.004	0.004	0.004	0.021
Unidentified sp. 3980_383	Basidiomycota	LC276982	279	279/279	100	0.011	0.045	0.029	-	0.008	0.004	0.021
Unidentified sp. 3980_392	Ascomycota	KP891492	239	239/239	100	0.035	0.025	0.030	-	0.004	0.002	0.021
Unidentified sp. 3980_457	Ascomycota	KT219383	264	250/256	98	0.002	-	0.001	0.137	-	0.068	0.021
Unidentified sp. 3980_347	Mucoromycota	KP891547	338	338/338	100	-	-	-	0.068	0.068	0.068	0.021
Penicillium sp. 3980_348	Ascomycota	JN246036	261	261/261	100	-	-	-	0.133	0.004	0.068	0.021
Unidentified sp. 3980_368	Basidiomycota	KM493245	276	276/276	100	-	-	-	0.036	0.095	0.066	0.020
Unidentified sp. 3980_369	Basidiomycota	KP898045	328	325/330	98	0.042	0.015	0.028	-	0.004	0.002	0.020
Tolypocladium album	Ascomycota	MH860832	261	261/261	100	-	-	-	0.109	0.028	0.068	0.021
Unidentified sp. 3980_448	Basidiomycota	KP891234	333	333/333	100	0.005	0.015	0.010	0.004	0.083	0.044	0.021
Unidentified sp. 3980_512	Ascomycota	KU063062	242	241/247	98	0.031	0.028	0.030	-	-	-	0.021
Unidentified sp. 3980_313	Ascomycota	MG827926	288	288/288	100	0.024	0.023	0.024	0.020	0.004	0.012	0.020
Laccaria bicolor	Basidiomycota	MF958448	301	301/301	100	-	-	-	0.044	0.087	0.066	0.020

Piloderma sphaerosporum	Basidiomycota	MK131527	292	292/292	100	0.002	-	0.001	0.125	0.004	0.064	0.020
Unidentified sp. 3980_430	Basidiomycota	MG827626	325	325/325	100	0.024	0.005	0.014	0.016	0.052	0.034	0.020
Botrytis cinerea	Ascomycota	MH997911	240	240/240	100	0.026	0.013	0.019	0.016	0.028	0.022	0.020
Unidentified sp. 3980_523	Ascomycota	KT244857	242	217/249	87	0.033	0.023	0.028	-	0.004	0.002	0.020
Unidentified sp. 3980_530	Ascomycota	FR682176	239	239/239	100	0.038	0.018	0.028	0.004	-	0.002	0.020
Unidentified sp. 3980_314	Basidiomycota	KU062416	289	274/284	96	0.002	-	0.001	0.072	0.052	0.062	0.019
Thelephoraceae sp. 3980_327	Basidiomycota	KJ188609	328	327/328	99	-	-	-	0.121	0.008	0.064	0.019
Unidentified sp. 3980_387	Basidiomycota	LT608046	303	302/303	99	-	-	-	0.092	0.036	0.064	0.019
Unidentified sp. 3980_409	Basidiomycota	FR682403	300	300/300	100	0.038	0.017	0.027	-	0.004	0.002	0.019
Unidentified sp. 3980_508	Ascomycota	KX220475	286	286/286	100	-	-	-	0.113	0.016	0.064	0.019
Unidentified sp. 3980_581	Ascomycota	KP897170	250	250/250	100	0.051	0.007	0.028	-	-	-	0.019
Aspergillus acidohumus	Ascomycota	KX423646	260	260/260	100	-	-	-	-	0.127	0.064	0.019
Unidentified sp. 3980_339	Basidiomycota	KP897461	281	280/282	99	0.027	0.027	0.027	-	-	-	0.019
Filobasidium wieringae	Basidiomycota	KY037853	329	329/329	100	0.026	0.027	0.026	-	0.004	0.002	0.019
Unidentified sp. 3980_415	Basidiomycota	KP897892	307	305/308	99	0.013	0.038	0.026	0.004	-	0.002	0.019
Paxillus sp. 3980_422	Basidiomycota	HF675251	329	329/329	100	0.035	0.020	0.027	-	-	-	0.019
Unidentified sp. 3980_445	Basidiomycota	KY558349	313	262/323	81	0.016	0.033	0.025	0.008	-	0.004	0.019
Unidentified sp. 3980_490	Ascomycota	KP897250	289	289/289	100	0.026	0.027	0.026	-	0.004	0.002	0.019
Unidentified sp. 3980_493	Basidiomycota	KP891318	248	248/248	100	0.027	0.025	0.026	0.004	-	0.002	0.019
Helotiales sp. 3980_504	Ascomycota	KJ826717	241	241/241	100	-	-	-	0.028	0.095	0.062	0.019
Unidentified sp. 3980_528	Basidiomycota	KX222572	201	201/201	100	-	-	-	0.088	0.036	0.062	0.019
Unidentified sp. 3980_544	Ascomycota	KP897349	250	250/250	100	0.046	0.010	0.027	-	-	-	0.019
Unidentified sp. 3980_565	Ascomycota	KT219358	247	243/249	98	-	-	-	0.016	0.107	0.062	0.019
Unidentified sp. 3980_370	Basidiomycota	EU516677	332	326/332	98	-	-	-	-	0.119	0.060	0.018
Unidentified sp. 3980_395	Ascomycota	KP897761	239	239/239	100	0.018	0.033	0.026	-	-	-	0.018
Unidentified sp. 3980_410	Ascomycota	KY006627	250	250/250	100	0.020	0.030	0.025	0.004	-	0.002	0.018
Unidentified sp. 3980_413	Basidiomycota	AY884240	291	290/291	99	-	-	-	-	0.119	0.060	0.018
Unidentified sp. 3980_417	Ascomycota	KR266711	257	257/257	100	-	-	-	-	0.119	0.060	0.018
Unidentified sp. 3980_451	Ascomycota	KX220991	249	247/249	99	0.029	0.022	0.025	0.004	-	0.002	0.018
Unidentified sp. 3980_452	Ascomycota	KP897522	245	245/245	100	0.022	0.028	0.025	-	0.004	0.002	0.018
Unidentified sp. 3980_500	Ascomycota	MG827436	247	247/247	100	0.037	0.013	0.024	-	0.008	0.004	0.018
Unidentified sp. 3980_539	Ascomycota	KM068401	241	238/239	99	-	-	-	0.028	0.091	0.060	0.018
Pleurophoma ossicola	Ascomycota	KR476737	250	250/250	100	0.024	0.025	0.024	0.008	-	0.004	0.018
Unidentified sp. 3980_398	Ascomycota	EU516990	226	193/244	79	0.009	0.033	0.022	0.004	0.012	0.008	0.018
Unidentified sp. 3980_402	Basidiomycota	MF570709	246	76/87	87	0.037	0.015	0.025	-	-	-	0.018
Unidentified sp. 3980_418	Basidiomycota	KX195491	329	328/329	99	0.016	0.033	0.025	-	-	-	0.018
Cortinarius pholideus	Basidiomycota	AY669694	285	285/285	100	0.013	0.035	0.024	-	0.004	0.002	0.018
Unidentified sp. 3980_446	Basidiomycota	KP891298	273	273/273	100	0.029	0.017	0.023	0.008	0.004	0.006	0.018
Helotiales sp. 3980_447	Ascomycota	KJ827304	240	240/240	100	0.002	-	0.001	-	0.111	0.056	0.018
Amanita fulva	Basidiomycota	MK522026	260	260/260	100	-	-	-	-	0.115	0.058	0.018
Unidentified sp. 3980_556	Basidiomycota	KX194090	305	305/305	100	0.026	0.013	0.019	0.028	-	0.014	0.018
Unidentified sp. 3980_558	Basidiomycota	MF971748	214	197/214	92	-	-	-	0.040	0.076	0.058	0.018
Sordariomycetes sp. 3980_602	Ascomycota	KR266182	248	235/249	94	-	-	-	0.105	0.012	0.058	0.018
Lactarius sp. 3980_607	Basidiomycota	LN829220	348	345/348	99	0.004	-	0.002	0.064	0.044	0.054	0.018
Unidentified sp. 3980_623	Ascomycota	MG827566	244	244/244	100	0.038	0.013	0.025	-	-	-	0.018
Unidentified sp. 3980_367	Zoopagomycota	KX223177	368	365/368	99	-	-	-	0.044	0.068	0.056	0.017

Inocybe sp. 3980_388	Basidiomycota	KT958922	279	279/279	100	-	-	-	0.105	0.008	0.056	0.017
Ustilago filiformis	Basidiomycota	MH855085	387	387/387	100	0.004	0.040	0.023	0.004	-	0.002	0.016
Hyaloscypha variabilis	Ascomycota	MK131649	238	238/238	100	-	-	-	0.064	0.048	0.056	0.017
Unidentified sp. 3980_416	Ascomycota	KM493097	253	51/52	98	-	-	-	0.076	0.036	0.056	0.017
Unidentified sp. 3980_443	Ascomycota	KT219675	236	234/236	99	-	-	-	0.076	0.036	0.056	0.017
Unidentified sp. 3980_453	Ascomycota	KC884316	241	239/241	99	0.013	0.032	0.023	0.004	-	0.002	0.016
Fusicladium cordae	Ascomycota	FN549911	264	264/264	100	0.016	0.032	0.024	-	-	-	0.017
Orbilia sp. 3980_459	Ascomycota	MG372374	279	279/279	100	0.038	0.010	0.024	0.004	-	0.002	0.017
Chaetothyriales sp. 3980_475	Ascomycota	KJ827326	253	248/256	97	-	-	-	0.004	0.107	0.056	0.017
Unidentified sp. 3980_517	Basidiomycota	JN032495	303	303/303	100	0.029	0.018	0.024	-	0.004	0.002	0.017
Unidentified sp. 3980_541	Basidiomycota	MG828150	245	245/245	100	0.018	0.025	0.022	0.008	-	0.004	0.016
Unidentified sp. 3980_614	Ascomycota	KJ827132	244	244/244	100	-	0.012	0.006	0.044	0.040	0.042	0.017
Unidentified sp. 3980_646	Ascomycota	MG760876	249	246/249	99	0.013	0.030	0.022	0.012	-	0.006	0.017
Amanita sp. 3980_384	Basidiomycota	LN829177	295	295/295	100	-	-	-	0.101	0.008	0.054	0.016
Unidentified sp. 3980_385	Ascomycota	KP891798	255	255/255	100	0.026	0.017	0.021	0.008	0.004	0.006	0.016
Unidentified sp. 3980_399	Basidiomycota	HQ433212	322	321/322	99	0.016	0.027	0.022	0.008	-	0.004	0.016
Unidentified sp. 3980_480	Basidiomycota	AM902000	282	281/282	99	0.020	0.023	0.022	-	0.008	0.004	0.016
Unidentified sp. 3980_485	Mucoromycota	KP897541	269	266/269	99	0.037	0.012	0.024	-	-	-	0.016
Pseudotomentella sp. 3980_522	Basidiomycota	KP125720	309	309/309	100	-	-	-	-	0.107	0.054	0.016
Ganoderma applanatum	Basidiomycota	MH320562	288	288/288	100	0.015	0.010	0.012	0.020	0.032	0.026	0.016
Unidentified sp. 3980_356	Basidiomycota	AY969500	312	293/302	97	0.004	0.040	0.023	-	-	-	0.016
Unidentified sp. 3980_376	Basidiomycota	KP897794	239	238/239	99	0.007	0.037	0.023	-	-	-	0.016
Tomentella stuposa	Basidiomycota	KM409444	314	314/314	100	-	-	-	0.076	0.028	0.052	0.016
Unidentified sp. 3980_412	Cryptomycota	LT608058	216	215/216	99	0.026	0.017	0.021	0.004	0.004	0.004	0.016
Solicoccozyma aeria	Basidiomycota	MK397489	324	324/324	100	-	-	-	-	0.103	0.052	0.016
Unidentified sp. 3980_440	Mucoromycota	FN610984	338	337/338	99	0.022	0.023	0.023	-	-	-	0.016
Unidentified sp. 3980_455	Basidiomycota	KX195148	295	295/295	100	-	-	-	0.092	0.012	0.052	0.016
Anthracobia melaloma	Ascomycota	MH857338	253	253/253	100	-	-	-	0.008	0.095	0.052	0.016
Unidentified sp. 3980_465	Basidiomycota	KU063474	296	294/296	99	0.004	0.038	0.022	-	-	-	0.015
Entoloma albogriseum	Basidiomycota	KJ705171	296	294/296	99	-	-	-	0.020	0.083	0.052	0.016
Unidentified sp. 3980_503	Ascomycota	KP897308	263	263/263	100	0.020	0.023	0.022	0.004	-	0.002	0.016
Unidentified sp. 3980_525	Ascomycota	LC309750	238	230/239	96	0.002	-	0.001	0.008	0.091	0.050	0.016
Unidentified sp. 3980_550	Basidiomycota	KP897194	281	281/281	100	0.015	0.028	0.022	-	-	-	0.015
Unidentified sp. 3980_573	Basidiomycota	KP888020	326	326/326	100	0.013	0.032	0.023	-	-	-	0.016
Unidentified sp. 3980_641	Ascomycota	MG827505	250	250/250	100	0.029	0.017	0.023	-	-	-	0.016
Unidentified sp. 3980_647	Ascomycota	KT220015	242	241/242	99	0.038	0.008	0.023	-	-	-	0.016
Unidentified sp. 3980_662	Basidiomycota	MG827564	235	235/235	100	0.040	0.007	0.023	-	-	-	0.016
Unidentified sp. 3980_371	Ascomycota	LT608052	252	250/252	99	-	-	-	0.076	0.024	0.050	0.015
Unidentified sp. 3980_394	Ascomycota	KT328674	257	220/261	84	0.005	0.035	0.021	-	0.004	0.002	0.015
Unidentified sp. 3980_456	Mucoromycota	AM260926	349	347/349	99	-	-	-	0.012	0.087	0.050	0.015
Unidentified sp. 3980_477	Ascomycota	KP892073	246	235/251	94	0.029	0.015	0.022	-	-	-	0.015
Unidentified sp. 3980_486	Basidiomycota	AB476473	321	321/321	100	0.040	0.003	0.021	0.004	-	0.002	0.015
Unidentified sp. 3980_509	Ascomycota	MG827565	249	244/249	98	0.024	0.018	0.021	-	0.004	0.002	0.015
Unidentified sp. 3980_518	Ascomycota	MG827733	257	257/257	100	0.013	0.023	0.018	0.008	0.008	0.008	0.015
Unidentified sp. 3980_532	Ascomycota	KP897278	269	269/269	100	0.020	0.023	0.022	-	-	-	0.015
Unidentified sp. 3980_568	Ascomycota	KT219535	211	206/211	98	-	-	-	0.004	0.095	0.050	0.015

Unidentified sp. 3980_840	Ascomycota	MG827431	249	249/249	100	0.035	0.010	0.022	-	-	-	0.015
Rhodotorula sp. 3980_397	Basidiomycota	DQ250660	301	283/285	99	-	-	-	0.084	0.012	0.048	0.015
Coleosporium sp. 3980_407	Basidiomycota	KY810470	327	317/328	97	0.009	0.032	0.021	-	-	-	0.015
Unidentified sp. 3980_435	Ascomycota	KT220003	250	250/250	100	-	0.002	0.001	0.052	0.040	0.046	0.015
Unidentified sp. 3980_441	Ascomycota	JN032493	257	257/257	100	-	-	-	0.052	0.044	0.048	0.015
Unidentified sp. 3980_464	Ascomycota	KP891453	210	210/210	100	0.007	0.032	0.020	0.004	-	0.002	0.015
Unidentified sp. 3980_494	Ascomycota	KP897259	262	262/262	100	0.031	0.012	0.021	-	-	-	0.015
Unidentified sp. 3980_499	Ascomycota	GU308354	248	239/240	99	-	-	-	0.012	0.083	0.048	0.015
Unidentified sp. 3980_535	Ascomycota	MG761085	244	244/244	100	0.035	0.008	0.021	-	-	-	0.015
Acrodontium sp. 3980_554	Ascomycota	MG543975	239	239/239	100	0.027	0.013	0.020	0.004	-	0.002	0.015
Unidentified sp. 3980_609	Ascomycota	KP897236	256	256/256	100	-	-	-	0.048	0.048	0.048	0.015
Unidentified sp. 3980_672	Basidiomycota	JN032502	305	299/305	98	0.042	0.002	0.021	-	-	-	0.015
Unidentified sp. 3980_389	Basidiomycota	KM494421	299	299/299	100	0.013	0.027	0.020	-	-	-	0.014
Unidentified sp. 3980_462	Basidiomycota	AB560523	303	303/303	100	-	-	-	0.060	0.032	0.046	0.014
Unidentified sp. 3980_463	Ascomycota	MG827785	249	249/249	100	0.027	0.013	0.020	-	-	-	0.014
Unidentified sp. 3980_469	Basidiomycota	MG757510	318	51/52	98	0.018	0.022	0.020	-	-	-	0.014
Unidentified sp. 3980_519	Basidiomycota	MF483718	313	313/313	100	0.004	0.035	0.020	-	-	-	0.014
Unidentified sp. 3980_524	Ascomycota	KP892306	247	247/247	100	0.029	0.012	0.020	-	-	-	0.014
Unidentified sp. 3980_534	Ascomycota	KT197173	238	226/239	95	-	-	-	0.076	0.016	0.046	0.014
Helotiales sp. 3980_538	Ascomycota	KY228805	240	234/241	97	-	-	-	0.068	0.024	0.046	0.014
Unidentified sp. 3980_552	Ascomycota	KP898168	247	215/255	84	0.024	0.017	0.020	-	-	-	0.014
Unidentified sp. 3980_584	Ascomycota	KX194763	235	232/235	99	0.027	0.012	0.019	0.004	-	0.002	0.014
Unidentified sp. 3980_592	Ascomycota	KP897266	246	246/246	100	0.024	0.017	0.020	-	-	-	0.014
Ucoromycotina sp. 3980_611	Mucoromycota	AB846975	300	300/300	100	-	-	-	0.092	-	0.046	0.014
Neurospora dictyophora	Ascomycota	MH862539	245	245/245	100	0.005	0.005	0.005	0.020	0.048	0.034	0.014
Oidiodendron flavum	Ascomycota	MH864869	236	236/236	100	-	-	-	0.072	0.020	0.046	0.014
Unidentified sp. 3980_836	Basidiomycota	KX222816	301	300/301	99	-	-	-	0.080	0.012	0.046	0.014
Unidentified sp. 3980_419	Mucoromycota	KP889402	354	327/364	90	-	-	-	0.084	0.004	0.044	0.013
Lycoperdon nigrescens	Basidiomycota	DQ112577	305	305/305	100	0.002	-	0.001	0.076	0.008	0.042	0.013
Unidentified sp. 3980_444	Ascomycota	MG828072	263	261/264	99	0.018	0.005	0.011	0.024	0.012	0.018	0.013
Pleosporales sp. 3980_468	Ascomycota	AB986471	256	256/256	100	-	0.035	0.018	0.004	-	0.002	0.013
Powellomyces sp. 3980_471	Chytridiomycota	JN943807	321	321/321	100	-	-	-	0.060	0.028	0.044	0.013
Buckleyzyma aurantiaca	Basidiomycota	KX096691	293	293/293	100	0.035	0.005	0.019	-	-	-	0.013
Unidentified sp. 3980_482	Basidiomycota	KP887970	294	294/294	100	0.011	0.025	0.018	-	-	-	0.013
Inocybe sp. 3980_501	Basidiomycota	MF352730	293	292/293	99	0.016	0.020	0.018	0.004	-	0.002	0.013
Didymocyrtis cladoniicola	Ascomycota	LC171648	251	251/251	100	0.022	0.013	0.017	-	0.008	0.004	0.013
Mortierellaceae sp. 3980_557	Mucoromycota	KR265938	340	340/340	100	-	-	-	-	0.087	0.044	0.013
Unidentified sp. 3980_560	Ascomycota	KT219621	248	248/248	100	-	0.037	0.019	-	-	-	0.013
Erysiphe cruciferarum	Ascomycota	KY660929	273	273/273	100	-	-	-	0.048	0.040	0.044	0.013
Unidentified sp. 3980_569	Basidiomycota	MG827592	329	329/329	100	-	-	-	0.048	0.040	0.044	0.013
Leptodontidium obscurum	Ascomycota	MH861893	242	242/242	100	-	-	-	0.024	0.064	0.044	0.013
Preussia funiculata	Ascomycota	AY943059	248	248/248	100	0.037	0.003	0.019	-	-	-	0.013
Unidentified sp. 3980_677	Ascomycota	KP897198	249	248/249	99	-	-	-	0.032	0.056	0.044	0.013
Pleosporales sp. 3980_679	Ascomycota	MF487331	290	290/290	100	0.002	-	0.001	0.036	0.048	0.042	0.013
Tetracladium sp. 3980_699	Ascomycota	MF615024	241	241/241	100	-	0.003	0.002	-	0.080	0.040	0.013
Unidentified sp. 3980_763	Ascomycota	KJ827805	258	246/260	95	-	0.003	0.002	0.024	0.056	0.040	0.013

Unidentified sp. 3980_768	Ascomycota	MG828100	249	249/249	100	-	0.002	0.001	0.048	0.036	0.042	0.013
Unidentified sp. 3980_776	Ascomycota	MG827787	246	246/246	100	0.029	0.010	0.019	-	-	-	0.013
Unidentified sp. 3980_483	Ascomycota	KU061831	238	231/238	97	-	-	-	0.084	-	0.042	0.013
Agaricales sp. 3980_488	Basidiomycota	FJ554408	309	307/312	98	0.020	0.017	0.018	-	-	-	0.013
Unidentified sp. 3980_495	Basidiomycota	KP898007	329	328/333	98	-	-	-	0.004	0.080	0.042	0.013
Unidentified sp. 3980_502	Ascomycota	KP897733	241	240/243	99	0.024	0.013	0.018	-	-	-	0.013
Unidentified sp. 3980_507	Ascomycota	KP897347	247	246/251	98	0.018	0.018	0.018	-	-	-	0.013
Unidentified sp. 3980_514	Ascomycota	MG827481	249	249/249	100	0.035	0.002	0.017	-	0.004	0.002	0.013
Hypocreales sp. 3980_520	Ascomycota	MF485410	273	271/274	99	0.005	0.023	0.015	-	0.012	0.006	0.012
Unidentified sp. 3980_526	Basidiomycota	GU328623	320	320/320	100	-	-	-	0.004	0.080	0.042	0.013
Unidentified sp. 3980_536	Ascomycota	KP897853	238	238/238	100	0.027	0.008	0.017	-	0.004	0.002	0.013
Metarhizium carneum	Ascomycota	MH864785	285	285/285	100	0.026	0.012	0.018	-	-	-	0.013
Unidentified sp. 3980_549	Ascomycota	AJ608950	269	269/269	100	-	-	-	0.060	0.024	0.042	0.013
Aspergillus vitricola	Ascomycota	MK367420	272	272/272	100	-	-	-	0.016	0.068	0.042	0.013
Leotiomycetes sp. 3980_589	Ascomycota	KR267121	239	239/239	100	0.009	0.012	0.010	0.012	0.024	0.018	0.013
Neoascochyta exitialis	Ascomycota	MH861347	249	249/249	100	-	-	-	0.012	0.072	0.042	0.013
Unidentified sp. 3980_599	Ascomycota	KP897891	244	244/244	100	0.020	0.015	0.017	-	0.004	0.002	0.013
Unidentified sp. 3980_621	Ascomycota	JF424297	249	247/250	99	0.022	0.015	0.018	-	-	-	0.013
Unidentified sp. 3980_659	Ascomycota	MF486297	250	250/250	100	0.016	0.018	0.017	0.004	-	0.002	0.013
Unidentified sp. 3980_700	Ascomycota	MG828397	247	246/249	99	0.007	0.007	0.007	0.052	-	0.026	0.013
Unidentified sp. 3980_897	Ascomycota	JQ312711	242	242/242	100	-	0.035	0.018	-	-	-	0.013
Unidentified sp. 3980_437	Basidiomycota	AM902019	307	307/307	100	0.020	0.013	0.017	-	0.004	0.002	0.012
Suillus sp. 3980_478	Basidiomycota	FN565360	329	329/329	100	0.018	0.013	0.016	0.008	-	0.004	0.012
Unidentified sp. 3980_484	Ascomycota	KX148023	247	247/247	100	0.027	0.007	0.017	-	0.004	0.002	0.012
Baeospora myosura	Basidiomycota	MH856301	299	299/299	100	0.029	0.007	0.017	-	-	-	0.012
Unidentified sp. 3980_537	Basidiomycota	KP898095	254	253/258	98	0.004	0.022	0.013	0.012	0.008	0.010	0.012
Helicorhoidion sp. 3980_545	Ascomycota	MH861094	239	239/239	100	-	-	-	0.060	0.020	0.040	0.012
Unidentified sp. 3980_561	Ascomycota	KX194413	238	238/238	100	0.015	0.018	0.017	0.004	-	0.002	0.012
Unidentified sp. 3980_587	Ascomycota	KU062995	211	211/211	100	-	0.002	0.001	0.004	0.072	0.038	0.012
Unidentified sp. 3980_595	Basidiomycota	KP897295	297	297/297	100	0.011	0.023	0.017	-	-	-	0.012
Cantharellales sp. 3980_627	Basidiomycota	EF611131	303	303/303	100	-	0.002	0.001	0.076	-	0.038	0.012
Taphrina purpurascens	Ascomycota	MH857503	306	305/306	99	0.029	0.007	0.017	-	-	-	0.012
Unidentified sp. 3980_644	Basidiomycota	MG827540	246	246/246	100	0.026	0.003	0.014	0.004	0.012	0.008	0.012
Unidentified sp. 3980_651	Basidiomycota	KM493046	243	201/244	82	0.020	0.013	0.017	0.004	-	0.002	0.012
Unidentified sp. 3980_653	Basidiomycota	KP891091	322	286/329	87	0.009	0.025	0.017	-	-	-	0.012
Talaromyces funiculosus	Ascomycota	MH865292	249	249/249	100	-	-	-	0.056	0.024	0.040	0.012
Unidentified sp. 3980_729	Ascomycota	KP897339	243	241/243	99	0.022	0.012	0.017	-	0.004	0.002	0.012
Unidentified sp. 3980_752	Ascomycota	MG827494	258	257/258	99	0.020	0.015	0.017	-	-	-	0.012
Unidentified sp. 3980_756	Ascomycota	KT219384	240	231/241	96	-	-	-	0.052	0.028	0.040	0.012
Unidentified sp. 3980_769	Ascomycota	KJ826822	248	243/249	98	0.020	0.015	0.017	-	-	-	0.012
Unidentified sp. 3980_492	Ascomycota	KP897403	263	262/263	99	0.024	0.010	0.017	-	-	-	0.012
Unidentified sp. 3980_546	Ascomycota	AM901875	254	253/254	99	0.031	0.002	0.016	0.004	-	0.002	0.012
Lactarius rufus	Basidiomycota	MG597391	355	355/355	100	-	-	-	0.076	-	0.038	0.012
Cryptococcus sp. 3980_576	Basidiomycota	LT904718	246	246/246	100	0.029	-	0.014	-	0.012	0.006	0.012
Slooffia sp. 3980_579	Basidiomycota	KY105427	308	303/308	97	-	0.007	0.003	0.012	0.044	0.028	0.011
Cercophora sp. 3980_603	Ascomycota	HQ631039	247	247/247	100	0.009	0.023	0.017	-	-	-	0.012

Penicillium sp. 3980_608	Ascomycota	FJ379815	263	252/264	95	-	-	-	-	0.076	0.038	0.012
Unidentified sp. 3980_617	Ascomycota	MG827811	304	303/304	99	-	-	-	0.052	0.024	0.038	0.012
Hohenbuehelia petaloides	Basidiomycota	KY679142	294	294/294	100	-	-	-	0.024	0.052	0.038	0.012
Unidentified sp. 3980_633	Ascomycota	FR682161	256	256/256	100	0.015	0.010	0.012	-	0.020	0.010	0.012
Unidentified sp. 3980_669	Ascomycota	KP892073	243	225/251	90	0.026	-	0.012	-	0.020	0.010	0.012
Unidentified sp. 3980_671	Ascomycota	KP897486	248	238/249	96	0.020	0.012	0.016	0.004	-	0.002	0.012
Unidentified sp. 3980_691	Ascomycota	KP898051	254	228/254	90	0.013	0.020	0.017	-	-	-	0.012
Oligoporus sp. 3980_692	Basidiomycota	KX845208	321	308/323	95	0.020	0.013	0.017	-	-	-	0.012
Unidentified sp. 3980_702	Ascomycota	KP897420	306	304/307	99	0.007	0.023	0.016	-	0.004	0.002	0.012
Coniochaeta angustispora	Ascomycota	MH860817	249	249/249	100	-	-	-	0.004	0.072	0.038	0.012
Unidentified sp. 3980_738	Ascomycota	KP891386	256	232/263	88	0.027	0.007	0.017	-	-	-	0.012
Unidentified sp. 3980_761	Basidiomycota	LT608118	303	303/303	100	-	-	-	0.036	0.040	0.038	0.012
Unidentified sp. 3980_784	Ascomycota	KP897431	272	246/289	85	-	0.030	0.016	0.004	-	0.002	0.012
Unidentified sp. 3980_461	Basidiomycota	KP891849	310	265/321	83	0.005	0.022	0.014	-	0.008	0.004	0.011
Unidentified sp. 3980_491	Basidiomycota	MF946272	300	300/300	100	-	-	-	0.004	0.068	0.036	0.011
Unidentified sp. 3980_527	Basidiomycota	KP897597	299	298/299	99	0.016	0.013	0.015	0.004	-	0.002	0.011
Symmetrospora gracilis	Basidiomycota	MG827628	301	301/301	100	0.024	0.007	0.015	0.004	-	0.002	0.011
Unidentified sp. 3980_571	Ascomycota	EU520640	249	249/249	100	-	-	-	0.020	0.052	0.036	0.011
Oidiodendron maius	Ascomycota	MH860824	237	237/237	100	-	-	-	0.032	0.040	0.036	0.011
Cladophialophora sp. 3980_578	Ascomycota	MG012688	253	251/253	99	-	-	-	0.024	0.048	0.036	0.011
Unidentified sp. 3980_580	Basidiomycota	MH697740	324	290/332	80	0.013	0.018	0.016	-	-	-	0.011
Unidentified sp. 3980_585	Ascomycota	KU837976	242	242/242	100	-	-	-	0.064	0.008	0.036	0.011
Unidentified sp. 3980_618	Ascomycota	KP897252	259	259/259	100	0.033	-	0.016	-	-	-	0.011
Unidentified sp. 3980_619	Basidiomycota	KX195741	256	249/256	97	0.011	0.020	0.016	-	-	-	0.011
Unidentified sp. 3980_640	Basidiomycota	MF570955	346	296/351	84	0.005	0.025	0.016	-	-	-	0.011
Unidentified sp. 3980_643	Basidiomycota	KP897674	305	305/310	98	0.005	0.025	0.016	-	-	-	0.011
Cortinarius caperatus	Basidiomycota	KU950452	294	294/294	100	0.016	0.005	0.010	0.008	0.016	0.012	0.011
Lyomyces sp. 3980_661	Basidiomycota	JX857785	283	283/283	100	-	-	-	0.072	-	0.036	0.011
Chalara sp. 3980_663	Ascomycota	DQ068981	240	240/240	100	0.004	-	0.002	0.020	0.044	0.032	0.011
Unidentified sp. 3980_675	Ascomycota	KP891386	253	219/261	84	0.015	0.015	0.015	-	-	-	0.010
Unidentified sp. 3980_697	Ascomycota	KT244138	265	231/260	89	0.004	0.025	0.015	0.004	-	0.002	0.011
Pertusariales sp. 3980_772	Ascomycota	KJ826968	251	248/252	98	-	-	-	0.024	0.048	0.036	0.011
Peniophora limitata	Basidiomycota	MH071727	287	287/287	100	0.002	0.015	0.009	0.032	-	0.016	0.011
Chalara sp. 3980_822	Ascomycota	FR667221	243	231/243	95	-	0.030	0.016	-	-	-	0.011
Unidentified sp. 3980_949	Ascomycota	FR682355	264	263/264	99	0.004	-	0.002	-	0.064	0.032	0.011
Unidentified sp. 3980_962	Basidiomycota	KU061651	293	293/293	100	-	-	-	0.048	0.024	0.036	0.011
Unidentified sp. 3980_467	Basidiomycota	FR682312	372	225/267	84	0.020	0.010	0.015	-	-	-	0.010
Suillus granulatus	Basidiomycota	KX230633	331	331/331	100	0.002	0.003	0.003	0.052	0.004	0.028	0.010
Unidentified sp. 3980_515	Ascomycota	KP897345	254	253/255	99	0.013	0.010	0.011	0.016	-	0.008	0.010
Mortierella sp. 3980_531	Mucoromycota	KR265936	350	350/350	100	0.026	0.005	0.015	-	-	-	0.010
Unidentified sp. 3980_566	Basidiomycota	KP897944	327	283/348	81	-	-	-	0.048	0.020	0.034	0.010
Unidentified sp. 3980_567	Ascomycota	KT219634	238	236/239	99	0.016	0.013	0.015	-	-	-	0.010
Rhizopogon sp. 3980_582	Basidiomycota	HG426016	341	341/341	100	0.002	0.025	0.014	-	-	-	0.010
Unidentified sp. 3980_588	Mucoromycota	KU559795	332	318/326	98	-	-	-	0.056	0.012	0.034	0.010
Unidentified sp. 3980_606	Ascomycota	MG828422	267	267/267	100	-	-	-	-	0.068	0.034	0.010
Rhodocollybia sp. 3980_624	Basidiomycota	LN714597	369	369/369	100	-	-	-	0.016	0.052	0.034	0.010

Myxotrichaceae sp. 3980_630	Ascomycota	KR266783	236	236/236	100	-	-	-	-	0.068	0.034	0.010
Unidentified sp. 3980_634	Ascomycota	KM494111	266	265/266	99	0.011	0.017	0.014	-	0.004	0.002	0.010
Unidentified sp. 3980_638	Ascomycota	KP898151	251	250/251	99	0.002	-	0.001	0.008	0.056	0.032	0.010
Sordariomycetes sp. 3980_645	Ascomycota	JX243908	269	269/269	100	0.026	0.005	0.015	-	-	-	0.010
Unidentified sp. 3980_673	Ascomycota	KP897474	243	243/243	100	0.013	0.015	0.014	0.004	-	0.002	0.010
Unidentified sp. 3980_696	Basidiomycota	FR682312	340	304/345	88	-	-	-	0.064	0.004	0.034	0.010
Apiotrichum gracile	Basidiomycota	MK268132	299	299/299	100	0.016	0.013	0.015	-	-	-	0.010
Sordariomycetes sp. 3980_711	Ascomycota	KX909189	261	261/261	100	0.029	0.002	0.015	-	-	-	0.010
Unidentified sp. 3980_716	Ascomycota	KP897537	242	240/242	99	0.004	0.025	0.015	-	-	-	0.010
Unidentified sp. 3980_736	Ascomycota	GQ159992	206	206/206	100	0.004	0.025	0.015	-	-	-	0.010
Mycosphaerellaceae sp. 3980_775	Ascomycota	FJ554069	246	246/246	100	0.024	0.005	0.014	-	0.004	0.002	0.010
Dothideomycetes sp. 3980_778	Ascomycota	KY430482	251	240/251	96	0.020	0.008	0.014	0.004	-	0.002	0.010
Unidentified sp. 3980_783	Ascomycota	MG828304	242	242/242	100	-	-	-	-	0.068	0.034	0.010
Taphrina vestergrenii	Ascomycota	NR_155886	315	314/315	99	0.020	0.007	0.013	0.008	-	0.004	0.010
Unidentified sp. 3980_823	Ascomycota	HM069448	242	242/242	100	0.013	0.017	0.015	-	-	-	0.010
Unidentified sp. 3980_851	Ascomycota	JQ313096	236	208/209	99	0.013	0.005	0.009	-	0.028	0.014	0.010
Unidentified sp. 3980_884	Ascomycota	KF617784	241	239/241	99	0.027	0.002	0.014	0.004	-	0.002	0.010
Unidentified sp. 3980_893	Ascomycota	KP892439	254	254/254	100	-	-	-	0.064	0.004	0.034	0.010
Helotiales sp. 3980_917	Ascomycota	HQ022246	242	233/242	96	-	-	-	0.020	0.048	0.034	0.010
Unidentified sp. 3980_1110	Ascomycota	FM992983	216	216/216	100	-	-	-	0.044	0.024	0.034	0.010
Armillaria sp. 3980_466	Basidiomycota	KY389149	477	476/477	99	0.022	0.007	0.014	-	-	-	0.010
Unidentified sp. 3980_479	Basidiomycota	KT196441	253	229/237	97	0.002	-	0.001	0.024	0.036	0.030	0.010
Unidentified sp. 3980_489	Basidiomycota	MF481616	367	340/378	90	-	-	-	0.020	0.044	0.032	0.010
Amanita muscaria	Basidiomycota	MK461189	319	319/319	100	-	-	-	0.040	0.024	0.032	0.010
Unidentified sp. 3980_529	Basidiomycota	KM494195	308	262/317	83	0.005	0.022	0.014	-	-	-	0.010
Filobasidium stepposum	Basidiomycota	MK554577	336	336/336	100	0.011	0.015	0.013	0.004	-	0.002	0.010
Unidentified sp. 3980_583	Basidiomycota	KP897391	294	274/275	99	0.007	0.020	0.014	-	-	-	0.010
Sordariomycetes sp. 3980_590	Ascomycota	KX909198	257	256/257	99	0.027	-	0.013	-	0.004	0.002	0.010
Unidentified sp. 3980_600	Ascomycota	MG827707	263	263/263	100	0.002	0.025	0.014	-	-	-	0.010
Unidentified sp. 3980_616	Basidiomycota	KM493088	277	277/277	100	-	0.027	0.014	-	-	-	0.010
Unidentified sp. 3980_635	Basidiomycota	AB476491	252	252/252	100	-	-	-	0.020	0.044	0.032	0.010
Unidentified sp. 3980_637	Ascomycota	KU063809	264	248/266	93	0.016	0.010	0.013	-	0.004	0.002	0.010
Solicoccozyma terreus	Basidiomycota	MK397479	326	326/326	100	0.018	0.008	0.013	0.004	-	0.002	0.010
Unidentified sp. 3980_649	Mucoromycota	KU062155	337	301/304	99	-	-	-	0.064	-	0.032	0.010
Unidentified sp. 3980_670	Ascomycota	NR_137807	236	223/240	93	-	-	-	0.060	0.004	0.032	0.010
Fusarium graminearum	Ascomycota	MK212894	245	245/245	100	0.009	0.015	0.012	-	0.008	0.004	0.010
Geomyces sp. 3980_693	Ascomycota	KC916694	239	236/239	99	0.007	0.020	0.014	-	-	-	0.010
Unidentified sp. 3980_698	Ascomycota	KU063136	245	245/245	100	0.020	0.008	0.014	-	-	-	0.010
Russula albonigra	Basidiomycota	MG597413	321	321/321	100	0.013	0.015	0.014	-	-	-	0.010
Unidentified sp. 3980_706	Ascomycota	KT219416	255	253/255	99	0.005	0.020	0.013	-	0.004	0.002	0.010
Helotiales sp. 3980_714	Ascomycota	JX507666	238	238/238	100	0.013	0.013	0.013	0.004	-	0.002	0.010
Unidentified sp. 3980_724	Basidiomycota	KP891242	245	243/245	99	-	-	-	0.064	-	0.032	0.010
Unidentified sp. 3980_733	Ascomycota	FJ475816	240	240/240	100	-	-	-	0.020	0.044	0.032	0.010
Unidentified sp. 3980_737	Basidiomycota	KP891645	333	124/152	82	-	-	-	0.060	0.004	0.032	0.010
Dothideomycetes sp. 3980_741	Ascomycota	MF615043	248	248/248	100	0.011	0.017	0.014	-	-	-	0.010
Unidentified sp. 3980_748	Ascomycota	FJ028718	283	253/293	86	-	-	-	0.036	0.028	0.032	0.010

Rhizopogon rubescens	Basidiomycota	LC198723	344	344/344	100	0.027	0.002	0.014	-	-	-	0.010
Unidentified sp. 3980_792	Ascomycota	DQ979666	265	225/225	100	0.009	0.017	0.013	0.004	-	0.002	0.010
Unidentified sp. 3980_829	Ascomycota	MG827459	249	249/249	100	0.026	0.003	0.014	-	-	-	0.010
Unidentified sp. 3980_992	Ascomycota	KU839285	238	238/238	100	-	-	-	0.004	0.060	0.032	0.010
Tremella globispora	Basidiomycota	NR_155889	238	234/239	98	0.026	0.002	0.013	0.004	-	0.002	0.010
Rhabdospora lupini	Ascomycota	MH859359	240	240/240	100	0.007	0.018	0.013	-	0.004	0.002	0.010
Cladophialophora minutissima	Ascomycota	MG597448	253	252/253	99	-	-	-	0.008	0.052	0.030	0.009
Lemonniera sp. 3980_575	Ascomycota	KX096679	241	241/241	100	0.009	0.010	0.010	0.008	0.008	0.008	0.009
Pleosporales sp. 3980_577	Ascomycota	KT269954	248	248/248	100	-	-	-	0.008	0.052	0.030	0.009
Cortinarius brunneus	Basidiomycota	MG597349	286	286/286	100	0.011	0.010	0.010	0.008	0.004	0.006	0.009
Cenococcum sp. 3980_604	Ascomycota	EU516732	241	240/241	99	-	-	-	0.020	0.040	0.030	0.009
Gymnopilus odini	Basidiomycota	MF039257	311	311/311	100	-	-	-	0.044	0.016	0.030	0.009
Unidentified sp. 3980_631	Ascomycota	KP897186	251	251/251	100	-	-	-	0.052	0.008	0.030	0.009
Unidentified sp. 3980_639	Basidiomycota	KU062351	300	276/279	99	0.013	0.007	0.010	-	0.016	0.008	0.009
Unidentified sp. 3980_652	Mucoromycota	MF569210	333	333/333	100	0.022	0.005	0.013	-	-	-	0.009
Unidentified sp. 3980_660	Basidiomycota	FJ820512	309	308/309	99	-	-	-	-	0.060	0.030	0.009
Unidentified sp. 3980_680	Ascomycota	KP897717	253	245/254	96	-	-	-	0.060	-	0.030	0.009
Metapochonia bulbillosa	Ascomycota	MH864652	279	279/279	100	0.015	0.012	0.013	-	-	-	0.009
Paraphaeomoniella sp.	Ascomycota	NR_137711	264	254/264	96	0.013	0.013	0.013	-	-	-	0.009
Godronia fuliginosa	Ascomycota	MH858760	235	235/235	100	-	-	-	0.024	0.036	0.030	0.009
Unidentified sp. 3980_731	Basidiomycota	KP891793	267	267/267	100	0.026	0.002	0.013	-	-	-	0.009
Unidentified sp. 3980_734	Basidiomycota	NR_137680	331	288/343	84	0.007	0.018	0.013	-	-	-	0.009
Unidentified sp. 3980_740	Ascomycota	KU934887	242	229/245	93	0.020	0.007	0.013	-	-	-	0.009
Unidentified sp. 3980_754	Basidiomycota	KP891555	297	297/297	100	0.015	0.012	0.013	-	-	-	0.009
Chalara piceae-abietis	Ascomycota	FR667230	240	237/240	99	0.013	0.007	0.010	-	0.016	0.008	0.009
Penicillium nodositatum	Ascomycota	MH971254	260	260/260	100	0.015	0.012	0.013	-	-	-	0.009
Unidentified sp. 3980_795	Ascomycota	KT194835	245	245/248	99	-	-	-	-	0.060	0.030	0.009
Sphaerostilbella sp. 3980_802	Ascomycota	MH858660	270	263/272	97	-	-	-	0.012	0.048	0.030	0.009
Unidentified sp. 3980_811	Ascomycota	AM901752	259	259/259	100	-	-	-	0.028	0.032	0.030	0.009
Unidentified sp. 3980_838	Basidiomycota	AF444533	314	287/319	90	-	-	-	0.056	0.004	0.030	0.009
Unidentified sp. 3980_950	Basidiomycota	KP897502	315	314/316	99	0.022	0.005	0.013	-	-	-	0.009
Unidentified sp. 3980_1042	Ascomycota	AM260905	213	213/213	100	0.004	0.022	0.013	-	-	-	0.009
Inocybe pusio	Basidiomycota	MH366589	312	237/237	100	-	-	-	-	0.056	0.028	0.008
Gliomastix murorum	Ascomycota	MH864097	272	272/272	100	-	-	-	0.024	0.032	0.028	0.008
Unidentified sp. 3980_591	Basidiomycota	MG827815	251	250/253	99	-	0.022	0.011	0.004	-	0.002	0.008
Mortierella zonata	Mucoromycota	JX975983	343	337/342	99	-	-	-	-	0.056	0.028	0.008
Unidentified sp. 3980_625	Basidiomycota	KP892243	332	163/206	79	-	-	-	0.024	0.032	0.028	0.008
Unidentified sp. 3980_642	Ascomycota	KT220084	250	250/250	100	0.011	0.013	0.012	-	-	-	0.008
Inocybe sp. 3980_658	Basidiomycota	KM409417	293	292/293	99	-	-	-	0.048	0.008	0.028	0.008
Unidentified sp. 3980_665	Basidiomycota	NR_073307	314	248/326	76	-	-	-	0.048	0.008	0.028	0.008
Leotiomycetes sp. 3980_681	Ascomycota	KX908528	249	244/250	98	0.013	0.010	0.011	0.004	-	0.002	0.008
Unidentified sp. 3980_685	Ascomycota	KX148014	259	247/262	94	0.005	0.013	0.010	0.012	-	0.006	0.008
Paraphoma chrysanthemicola	Ascomycota	MH063752	235	235/235	100	0.011	0.013	0.012	-	-	-	0.008
Tomentella fuscocinerea	Basidiomycota	GU214810	314	312/314	99	0.002	0.003	0.003	0.020	0.024	0.022	0.008
Helotiales sp. 3980_723	Ascomycota	KU556592	242	240/242	99	-	-	-	0.040	0.016	0.028	0.008
Erythrobasidium hasegawianum	Basidiomycota	GQ911540	318	316/318	99	-	-	-	0.052	0.004	0.028	0.008

Unidentified sp. 3980_766	Basidiomycota	MF570638	279	229/294	78	0.002	0.022	0.012	-	-	-	0.008
Unidentified sp. 3980_774	Ascomycota	NR_160092	270	89/96	93	0.004	0.020	0.012	-	-	-	0.008
Unidentified sp. 3980_785	Ascomycota	KC966234	243	242/243	99	0.018	0.007	0.012	-	-	-	0.008
Unidentified sp. 3980_805	Ascomycota	FR682205	253	236/254	93	-	0.023	0.012	-	-	-	0.008
Unidentified sp. 3980_807	Ascomycota	KX194584	233	230/233	99	0.015	0.008	0.011	-	0.004	0.002	0.008
Rhizopogon luteorubescens	Basidiomycota	KX833229	334	333/335	99	-	-	-	0.004	0.052	0.028	0.008
Exophiala equina	Ascomycota	MH864584	284	284/284	100	-	-	-	0.020	0.036	0.028	0.008
Unidentified sp. 3980_940	Basidiomycota	KP897834	250	250/251	99	-	-	-	0.044	0.012	0.028	0.008
Acrodontium luzulae	Ascomycota	NR_154720	239	239/239	100	0.009	0.015	0.012	-	-	-	0.008
Unidentified sp. 3980_959	Ascomycota	MG828146	273	273/273	100	0.024	0.002	0.012	-	-	-	0.008
Unidentified sp. 3980_978	Ascomycota	KP892073	245	228/250	91	0.013	0.012	0.012	-	-	-	0.008
Agyriales sp. 3980_987	Ascomycota	MF485434	273	273/273	100	-	0.023	0.012	-	-	-	0.008
Unidentified sp. 3980_542	Mucoromycota	MF569741	359	319/361	88	-	-	-	0.044	0.008	0.026	0.008
Unidentified sp. 3980_574	Basidiomycota	HQ433134	309	309/309	100	0.007	0.015	0.011	-	-	-	0.008
Knufia sp. 3980_622	Ascomycota	JX843780	267	255/267	96	0.015	0.007	0.010	-	0.004	0.002	0.008
Unidentified sp. 3980_636	Mucoromycota	MF571163	286	281/286	98	0.009	0.013	0.011	-	-	-	0.008
Unidentified sp. 3980_657	Ascomycota	MH865053	241	219/243	90	-	-	-	0.052	-	0.026	0.008
Stropharia cyanea	Basidiomycota	KT336609	295	295/295	100	0.018	0.005	0.011	-	-	-	0.008
Ceratobasidium sp. 3980_682	Basidiomycota	MG597338	324	324/324	100	0.011	0.008	0.010	0.004	0.004	0.004	0.008
Unidentified sp. 3980_688	Basidiomycota	KP898078	305	297/306	97	-	-	-	0.044	0.008	0.026	0.008
Penicillium brevicompactum	Ascomycota	MH865309	255	255/255	100	0.002	0.020	0.011	-	-	-	0.008
Ramaria apiculata	Basidiomycota	AJ408385	309	305/309	99	0.013	0.010	0.011	-	-	-	0.008
Unidentified sp. 3980_709	Basidiomycota	KP891772	297	296/297	99	0.004	0.005	0.004	-	0.032	0.016	0.008
Unidentified sp. 3980_718	Ascomycota	HM240094	256	253/256	99	-	-	-	0.036	0.016	0.026	0.008
Unidentified sp. 3980_722	Basidiomycota	MG827534	296	296/296	100	0.022	0.002	0.011	-	-	-	0.008
Exobasidium sp. 3980_726	Basidiomycota	KR265974	287	271/288	94	0.005	0.017	0.011	-	-	-	0.008
Leotiomycetes sp. 3980_745	Ascomycota	KJ826752	242	241/242	99	0.022	0.002	0.011	-	-	-	0.008
Unidentified sp. 3980_759	Basidiomycota	KP892264	304	303/306	99	0.022	0.002	0.011	-	-	-	0.008
Unidentified sp. 3980_781	Ascomycota	KJ827105	254	254/254	100	-	-	-	-	0.052	0.026	0.008
Orbilia sp. 3980_787	Ascomycota	MH221060	280	272/280	97	0.016	0.007	0.011	-	-	-	0.008
Unidentified sp. 3980_794	Cryptomycota	KU062065	219	218/223	98	-	-	-	0.048	0.004	0.026	0.008
Unidentified sp. 3980_796	Ascomycota	HQ701751	244	244/244	100	0.005	0.017	0.011	-	-	-	0.008
Unidentified sp. 3980_808	Ascomycota	KU061727	244	244/245	99	-	-	-	0.024	0.028	0.026	0.008
Unidentified sp. 3980_809	Ascomycota	KP897227	251	251/251	100	0.005	0.002	0.003	0.004	0.004	0.004	0.004
Unidentified sp. 3980_817	Ascomycota	KU687395	250	247/249	99	0.015	0.008	0.011	-	-	-	0.008
Unidentified sp. 3980_843	Basidiomycota	KT241667	235	215/240	90	0.013	0.008	0.010	-	-	-	0.007
Desmazierella acicola	Ascomycota	MG098266	269	269/269	100	0.005	0.017	0.011	-	-	-	0.008
Unidentified sp. 3980_877	Basidiomycota	MG231611	228	62/68	91	0.016	0.005	0.010	-	-	-	0.007
Lyomyces sambuci	Basidiomycota	JX857727	290	290/290	100	0.004	0.002	0.003	0.012	0.028	0.020	0.008
Lecideales sp. 3980_95	Ascomycota	KJ827126	239	239/239	100	0.009	0.013	0.011	-	-	-	0.008
Pucciniomycetes sp. 3980_1018	Basidiomycota	JF705944	299	297/299	99	-	-	-	0.052	-	0.026	0.008
Chaetothyriales sp. 3980_1028	Ascomycota	KJ827147	257	256/257	99	-	-	-	-	0.052	0.026	0.008
Sarea resinae	Ascomycota	MH857390	249	249/249	100	0.009	0.013	0.011	-	-	-	0.008
Gemmamyces piceae	Ascomycota	NR_147687	245	245/245	100	-	-	-	0.020	0.032	0.026	0.008
Phaeotremella sp. 3980_615	Basidiomycota	MF076919	251	237/251	94	0.016	0.003	0.010	-	0.004	0.002	0.007
Cortinarius diasemospermus	Basidiomycota	KX355534	277	277/277	100	0.004	-	0.002	0.040	-	0.020	0.007

Chaetothyriales sp. 3980_666	Ascomycota	MH451802	256	251/256	98	-	-	-	0.028	0.020	0.024	0.007
Saccharomycetes sp. 3980_674	Ascomycota	MH451401	294	294/294	100	-	-	-	0.048	-	0.024	0.007
Unidentified sp. 3980_686	Ascomycota	MG827979	256	256/256	100	0.013	0.007	0.010	-	-	-	0.007
Unidentified sp. 3980_689	Basidiomycota	FR682252	294	294/295	99	0.013	0.007	0.010	0.004	-	0.002	0.007
Pseudevernia furfuracea	Ascomycota	GU300785	244	244/244	100	0.013	0.008	0.010	-	-	-	0.007
Unidentified sp. 3980_721	Ascomycota	KP898138	250	247/250	99	0.002	0.018	0.010	-	-	-	0.007
Infundichalara microchona	Ascomycota	KF359590	240	239/240	99	0.005	0.007	0.006	0.004	0.016	0.010	0.007
Fomitopsis pinicola	Basidiomycota	MH931272	293	293/293	100	-	-	-	0.012	0.036	0.024	0.007
Rhizoctonia carotae	Basidiomycota	MH861139	298	296/298	99	0.015	0.005	0.010	0.004	-	0.002	0.007
Unidentified sp. 3980_779	Ascomycota	KP891264	308	299/308	97	0.002	0.018	0.010	-	-	-	0.007
Serpula himantioides	Basidiomycota	GU187545	292	292/292	100	0.018	0.003	0.010	-	-	-	0.007
Mycena sp. 3980_814	Basidiomycota	MF943119	311	307/312	98	0.007	0.013	0.010	-	-	-	0.007
Unidentified sp. 3980_824	Ascomycota	NR_160207	241	203/255	80	0.002	0.002	0.002	-	0.040	0.020	0.007
Penicillium arenicola	Ascomycota	NR_160106	265	265/265	100	0.020	-	0.010	-	-	-	0.007
Chrysozyma griseoflava	Basidiomycota	KY102551	328	325/328	99	-	-	-	0.028	0.020	0.024	0.007
Unidentified sp. 3980_839	Ascomycota	HQ433050	259	258/259	99	-	0.020	0.010	-	-	-	0.007
Sistotrema sp. 3980_845	Basidiomycota	JX561240	302	287/302	95	0.007	-	0.003	0.032	-	0.016	0.007
Unidentified sp. 3980_853	Ascomycota	KP898086	247	244/247	99	-	-	-	0.024	0.024	0.024	0.007
Rhodotorula sp. 3980_861	Basidiomycota	DQ250654	302	275/286	96	0.016	0.005	0.010	-	-	-	0.007
Unidentified sp. 3980_874	Basidiomycota	KP891580	266	266/266	100	-	-	-	0.008	0.040	0.024	0.007
Unidentified sp. 3980_891	Basidiomycota	KP891937	303	303/303	100	0.016	0.005	0.010	-	-	-	0.007
Unidentified sp. 3980_899	Basidiomycota	KR266141	300	270/303	89	0.009	0.012	0.010	-	-	-	0.007
Unidentified sp. 3980_904	Ascomycota	MG828044	249	249/249	100	-	0.020	0.010	-	-	-	0.007
Unidentified sp. 3980_924	Ascomycota	AY599236	267	263/267	99	0.007	0.012	0.010	-	-	-	0.007
Unidentified sp. 3980_925	Ascomycota	KR266590	234	234/234	100	-	-	-	0.004	0.044	0.024	0.007
Unidentified sp. 3980_982	Ascomycota	KP891094	268	263/268	98	-	-	-	0.004	0.044	0.024	0.007
Helotiales sp. 3980_990	Ascomycota	MH451440	234	233/234	99	-	-	-	0.036	0.012	0.024	0.007
Helotiales sp. 3980_1058	Ascomycota	KX610429	240	240/240	100	-	-	-	0.032	0.016	0.024	0.007
Unidentified sp. 3980_1062	Ascomycota	MG827535	244	244/244	100	0.002	0.018	0.010	-	-	-	0.007
Unidentified sp. 3980_1071	Ascomycota	KU188654	242	241/242	99	-	0.020	0.010	-	-	-	0.007
Agaricales sp. 3980_1092	Basidiomycota	MF486014	256	255/256	99	-	-	-	0.036	0.012	0.024	0.007
Unidentified sp. 3980_1164	Ascomycota	KX192562	250	247/250	99	0.020	0.002	0.010	-	-	-	0.007
Cenangium ferruginosum	Ascomycota	MH859846	239	239/239	100	0.015	0.007	0.010	-	-	-	0.007
Inocybe sp. 3980_654	Basidiomycota	JX029930	288	288/288	100	-	-	-	0.044	-	0.022	0.007
Unidentified sp. 3980_690	Basidiomycota	MG827690	266	256/266	96	0.002	0.013	0.008	-	0.008	0.004	0.007
Unidentified sp. 3980_793	Ascomycota	MF487303	235	208/235	89	0.002	0.017	0.010	-	-	-	0.007
Metarhizium anisopliae	Ascomycota	MK142277	270	270/270	100	-	-	-	0.016	0.028	0.022	0.007
Eurotiomycetes sp. 3980_828	Ascomycota	KP991835	272	271/272	99	0.020	-	0.010	-	-	-	0.007
Unidentified sp. 3980_846	Ascomycota	HQ432986	240	240/240	100	-	-	-	0.024	0.020	0.022	0.007
Unidentified sp. 3980_856	Ascomycota	KM493818	250	250/250	100	0.004	0.013	0.009	-	0.004	0.002	0.007
Sphaerulina azaleae	Ascomycota	MH865035	238	238/238	100	0.009	0.010	0.010	-	-	-	0.007
Ophiognomonia alni-viridis	Ascomycota	MH864899	258	254/258	98	0.005	0.002	0.003	0.012	0.016	0.014	0.007
Unidentified sp. 3980_903	Ascomycota	MG827923	243	243/243	100	0.015	0.003	0.009	0.004	-	0.002	0.007
Unidentified sp. 3980_912	Ascomycota	FJ197927	270	270/270	100	0.002	0.017	0.010	-	-	-	0.007
Unidentified sp. 3980_913	Ascomycota	HQ433019	238	223/239	93	0.011	0.007	0.009	-	0.004	0.002	0.007
Trichoderma aerugineum	Ascomycota	NR_134379	266	262/267	98	0.005	0.005	0.005	-	0.020	0.010	0.007

Unidentified sp. 3980_957	Ascomycota	KJ827417	248	222/251	88	-	-	-	0.020	0.024	0.022	0.007
Dermateaceae sp. 3980_973	Ascomycota	FJ475779	242	241/242	99	0.020	-	0.010	-	-	-	0.007
Unidentified sp. 3980_975	Ascomycota	MG827704	244	244/244	100	-	-	-	-	0.044	0.022	0.007
Sordariomycetes sp. 3980_1000	Ascomycota	MH450927	244	244/244	100	-	-	-	0.044	-	0.022	0.007
Unidentified sp. 3980_1059	Ascomycota	JF424297	246	235/252	93	-	-	-	0.044	-	0.022	0.007
Unidentified sp. 3980_1152	Ascomycota	KP889536	262	258/263	98	-	0.018	0.010	-	-	-	0.007
Unidentified sp. 3980_1194	Ascomycota	MG827473	250	250/250	100	-	-	-	0.024	0.020	0.022	0.007
Unidentified sp. 3980_1281	Basidiomycota	KU062524	291	289/291	99	0.015	0.005	0.010	-	-	-	0.007
Cortinariaceae sp. 3980_1293	Basidiomycota	JN847504	236	235/236	99	-	-	-	0.008	0.036	0.022	0.007
Unidentified sp. 3980_1602	Ascomycota	MF347758	238	237/239	99	0.018	-	0.009	0.004	-	0.002	0.007
Ampulloclitocybe clavipes	Basidiomycota	LC370431	308	305/308	99	-	-	-	0.036	0.008	0.022	0.007
Helotiales sp. 3980_1990	Ascomycota	LC218320	240	240/240	100	-	-	-	0.008	0.036	0.022	0.007
Leptodontidium camptobactrum	Ascomycota	MH857172	238	238/238	100	0.018	-	0.009	-	-	-	0.006
Unidentified sp. 3980_720	Basidiomycota	GU328564	306	296/306	97	-	-	-	0.020	0.016	0.018	0.005
Unidentified sp. 3980_725	Ascomycota	KT219961	214	210/214	98	-	-	-	0.024	0.016	0.020	0.006
Cortinarius fulvescens	Basidiomycota	MG597369	281	281/284	99	-	-	-	0.032	0.004	0.018	0.005
Unidentified sp. 3980_743	Ascomycota	KU189056	270	269/270	99	-	-	-	0.028	0.012	0.020	0.006
Unidentified sp. 3980_749	Ascomycota	KP898194	291	290/291	99	0.002	0.015	0.009	-	-	-	0.006
Unidentified sp. 3980_789	Basidiomycota	JQ313112	319	274/326	84	-	-	-	0.024	0.016	0.020	0.006
Sistotrema sp. 3980_791	Basidiomycota	LC015733	317	316/317	99	0.013	0.005	0.009	-	-	-	0.006
Unidentified sp. 3980_797	Ascomycota	KP897572	289	273/289	94	0.015	0.003	0.009	-	-	-	0.006
Syncephalis sp. 3980_841	Zoopagomycota	KU317676	366	357/368	97	0.002	-	0.001	-	0.036	0.018	0.006
Unidentified sp. 3980_857	Ascomycota	KP897413	248	248/248	100	0.018	-	0.009	-	-	-	0.006
Phaeosphaeria eustoma	Ascomycota	MH862385	245	245/245	100	0.005	0.008	0.007	-	0.008	0.004	0.006
Claussenomyces kirschsteinianus	Ascomycota	KY689631	238	238/238	100	0.018	-	0.009	-	-	-	0.006
Neohortaea acidophila	Ascomycota	GU214636	242	201/205	98	0.011	0.007	0.009	-	-	-	0.006
Neosetophoma italica	Ascomycota	LC206635	249	249/249	100	0.013	0.003	0.008	0.004	-	0.002	0.006
Unidentified sp. 3980_880	Basidiomycota	KP897682	295	295/295	100	0.018	-	0.009	-	-	-	0.006
Unidentified sp. 3980_886	Ascomycota	KP897725	260	246/262	94	0.011	0.007	0.009	-	-	-	0.006
Unidentified sp. 3980_890	Ascomycota	HQ433026	213	211/213	99	0.013	0.005	0.009	-	-	-	0.006
Chaetothyriales sp. 3980_931	Ascomycota	KP400577	289	289/289	100	-	-	-	-	0.040	0.020	0.006
Amphinema sp. 3980_935	Basidiomycota	EU668284	273	273/273	100	-	-	-	0.040	-	0.020	0.006
Leccinum scabrum	Basidiomycota	AY853567	390	389/390	99	0.015	0.002	0.008	-	0.004	0.002	0.006
Dothideomycetes sp. 3980_970	Ascomycota	MF486298	251	245/252	97	-	-	-	0.016	0.020	0.018	0.005
Pezizomycotina sp. 3980_1002	Ascomycota	FJ553528	259	258/259	99	0.016	-	0.008	0.004	-	0.002	0.006
Unidentified sp. 3980_1012	Ascomycota	GQ280580	246	226/257	88	0.002	0.002	0.002	-	0.032	0.016	0.006
Unidentified sp. 3980_1107	Basidiomycota	KJ008838	254	254/254	100	-	-	-	0.016	0.024	0.020	0.006
Unidentified sp. 3980_1156	Ascomycota	MF976075	270	261/270	97	-	0.017	0.009	-	-	-	0.006
Paramyrothecium parvum	Ascomycota	MH855673	284	284/284	100	-	-	-	0.040	-	0.020	0.006
Unidentified sp. 3980_1206	Basidiomycota	KP897560	322	322/323	99	-	-	-	0.008	0.032	0.020	0.006
Cortinarius sp. 3980_1246	Basidiomycota	JQ711807	276	276/276	100	0.013	0.005	0.009	-	-	-	0.006
Unidentified sp. 3980_1285	Ascomycota	KP891386	256	255/256	99	-	-	-	0.004	0.036	0.020	0.006
Unidentified sp. 3980_1399	Ascomycota	KU062760	252	252/254	99	-	-	-	0.024	0.016	0.020	0.006
Penicillium westlingii	Ascomycota	MH865963	260	260/260	100	0.004	0.013	0.009	-	-	-	0.006
Unidentified sp. 3980_2181	Ascomycota	JF449830	312	291/316	92	0.016	-	0.008	-	-	-	0.005
Unidentified sp. 3980_684	Ascomycota	HQ650647	253	228/255	89	0.011	0.005	0.008	-	-	-	0.005

Taphrina nana	Ascomycota	MH857501	293	293/293	100	-	0.002	0.001	-	-	-	0.001
Strobilurus sp. 3980_735	Basidiomycota	KY430535	380	380/380	100	0.009	0.005	0.007	-	0.004	0.002	0.005
Cyphobasidiales sp. 3980_742	Basidiomycota	KU948755	289	276/287	96	-	-	-	0.036	-	0.018	0.005
Coleosporium euphrasiae	Basidiomycota	KY810469	624	342/342	100	-	0.015	0.008	-	-	-	0.005
Pezizomycotina sp. 3980_806	Ascomycota	MF614995	241	235/241	98	0.007	0.008	0.008	-	-	-	0.005
Tremellomycetes sp. 3980_810	Basidiomycota	MH450959	305	305/305	100	0.007	0.007	0.007	0.004	-	0.002	0.005
Dothideales sp. 3980_813	Ascomycota	KJ827737	259	242/243	99	0.004	0.002	0.003	0.020	0.004	0.012	0.005
Bacidia arceutina	Ascomycota	FR799126	248	232/233	99	0.007	0.008	0.008	-	-	-	0.005
Unidentified sp. 3980_835	Ascomycota	AM901896	257	257/257	100	0.002	0.012	0.007	0.004	-	0.002	0.005
Onygenales sp. 3980_858	Ascomycota	MF487394	215	207/217	95	0.009	0.007	0.008	-	-	-	0.005
Stereum rugosum	Basidiomycota	AM269811	291	291/291	100	0.005	0.010	0.008	-	-	-	0.005
Pochonia chlamydosporia	Ascomycota	MH424152	287	287/287	100	0.002	0.013	0.008	-	-	-	0.005
Unidentified sp. 3980_922	Basidiomycota	JF705944	303	278/303	92	-	-	-	-	0.036	0.018	0.005
Cryptococcus sp. 3980_928	Basidiomycota	LT548267	318	316/319	99	-	-	-	0.036	-	0.018	0.005
Unidentified sp. 3980_932	Ascomycota	LT608097	243	243/243	100	0.005	0.008	0.007	-	0.004	0.002	0.005
Dioszegia fristingensis	Basidiomycota	KX096671	217	217/217	100	0.015	0.002	0.008	-	-	-	0.005
Unidentified sp. 3980_958	Ascomycota	KP898176	258	246/259	95	-	0.015	0.008	-	-	-	0.005
Thysanophora penicillioides	Ascomycota	MF614987	254	254/254	100	-	-	-	0.036	-	0.018	0.005
Unidentified sp. 3980_1015	Ascomycota	KC965637	212	211/212	99	-	-	-	0.024	0.012	0.018	0.005
Unidentified sp. 3980_1024	Ascomycota	KP897431	283	273/286	95	0.007	0.008	0.008	-	-	-	0.005
Pleosporales sp. 3980_1027	Ascomycota	KR909165	248	248/248	100	0.009	0.005	0.007	-	0.004	0.002	0.005
Unidentified sp. 3980_1045	Basidiomycota	MF666584	209	199/200	99	0.004	0.005	0.004	0.004	0.012	0.008	0.005
Unidentified sp. 3980_1046	Basidiomycota	KP891218	319	318/319	99	-	-	-	-	0.036	0.018	0.005
Pleosporales sp. 3980_1094	Ascomycota	MH450996	251	251/251	100	-	0.015	0.008	-	-	-	0.005
Leotiomyceta sp. 3980_1145	Ascomycota	KF359573	249	248/249	99	0.016	-	0.008	-	-	-	0.005
Unidentified sp. 3980_1167	Basidiomycota	NR_077095	302	251/307	82	0.005	0.008	0.007	-	0.004	0.002	0.005
Unidentified sp. 3980_1176	Ascomycota	MF666033	251	200/200	100	0.015	-	0.007	0.004	-	0.002	0.005
Unidentified sp. 3980_1208	Ascomycota	FR682254	256	256/257	99	-	-	-	-	0.036	0.018	0.005
Unidentified sp. 3980_1226	Basidiomycota	KU063214	313	285/288	99	-	-	-	0.024	0.012	0.018	0.005
Unidentified sp. 3980_1227	Ascomycota	KU063026	258	257/259	99	0.007	0.008	0.008	-	-	-	0.005
Pleosporales sp. 3980_1298	Ascomycota	MF486226	250	248/251	99	-	-	-	0.004	0.032	0.018	0.005
Sordariomycetes sp. 3980_1342	Ascomycota	JN655620	238	237/238	99	0.013	0.003	0.008	-	-	-	0.005
Botryosphaeriales sp. 3980_1357	Ascomycota	KJ827062	243	239/244	98	0.009	0.005	0.007	-	-	-	0.005
Cortinarius hemitrichus	Basidiomycota	MH270626	280	280/280	100	0.015	0.002	0.008	-	-	-	0.005
Unidentified sp. 3980_1634	Ascomycota	KP897485	255	255/255	100	-	-	-	0.036	-	0.018	0.005
Hyphodontia pallidula	Basidiomycota	KP814392	286	284/286	99	-	-	-	0.028	0.008	0.018	0.005
Unidentified sp. 3980_747	Mucoromycota	KT736107	272	244/261	93	-	-	-	0.012	0.020	0.016	0.005
Thelephora terrestris	Basidiomycota	MH861911	313	312/313	99	0.002	0.002	0.002	0.024	-	0.012	0.005
Tylospora fibrillosa	Basidiomycota	MF926576	287	287/287	100	-	0.005	0.003	-	0.020	0.010	0.005
Mucor hiemalis	Mucoromycota	MF615073	268	267/269	99	0.009	0.002	0.005	0.004	0.004	0.004	0.005
Oberwinklerozyma silvestris	Basidiomycota	KY104386	316	311/316	98	0.005	0.007	0.006	-	0.004	0.002	0.005
Hebeloma dunense	Basidiomycota	MK281078	308	308/310	99	-	0.005	0.003	0.008	0.012	0.010	0.005
Fusicladium fagi	Ascomycota	LS998793	240	237/240	99	0.002	0.010	0.006	-	0.004	0.002	0.005
Cryptococcus sp. 3980_819	Basidiomycota	AF444361	317	316/317	99	0.007	0.007	0.007	-	-	-	0.005
Stereopsis vitellina	Basidiomycota	JN649374	298	298/298	100	-	-	-	0.016	0.016	0.016	0.005
Melanomma pulvis-pyrius	Ascomycota	MH863714	244	244/244	100	0.002	0.010	0.006	-	0.004	0.002	0.005

Unidentified sp. 3980_827	Ascomycota	KX148021	259	245/262	94	-	0.010	0.005	0.004	0.004	0.004	0.005
Unidentified sp. 3980_830	Ascomycota	FR682188	243	220/248	89	0.015	-	0.007	-	-	-	0.005
Leucosporidium intermedium	Basidiomycota	KY104186	307	307/307	100	0.004	0.007	0.005	0.008	-	0.004	0.005
Diaporthe sp. 3980_862	Ascomycota	MK012547	255	255/255	100	0.002	0.012	0.007	-	-	-	0.005
Cenococcum sp. 3980_863	Ascomycota	GU180255	241	239/242	99	-	-	-	0.016	0.016	0.016	0.005
Parapyrenochaeta sp. 3980_878	Ascomycota	MK441755	254	254/254	100	-	-	-	-	0.032	0.016	0.005
Spizellomycetales sp. 3980_882	Chytridiomycota	MF482712	333	330/333	99	0.007	0.005	0.006	-	0.004	0.002	0.005
Tremellomycetes sp. 3980_910	Basidiomycota	MH450808	252	252/252	100	-	-	-	0.016	0.016	0.016	0.005
Caloplaca ulcerosa	Ascomycota	GU080299	254	254/254	100	0.007	0.007	0.007	-	-	-	0.005
Coprinellus disseminatus	Basidiomycota	MG597342	301	301/301	100	-	-	-	0.016	0.016	0.016	0.005
Unidentified sp. 3980_953	Ascomycota	KF296986	240	240/240	100	-	0.013	0.007	-	-	-	0.005
Unidentified sp. 3980_964	Basidiomycota	MF483391	345	228/273	84	0.002	0.012	0.007	-	-	-	0.005
Unidentified sp. 3980_980	Ascomycota	MF594688	266	229/276	83	-	-	-	0.032	-	0.016	0.005
Unidentified sp. 3980_989	Basidiomycota	KP891533	334	333/334	99	-	0.013	0.007	-	-	-	0.005
Coniothyrium cereale	Ascomycota	MH863077	249	249/249	100	0.005	0.008	0.007	-	-	-	0.005
Mycosymbioces mycenaphila	Ascomycota	NR_137807	238	238/238	100	0.011	0.003	0.007	-	-	-	0.005
Unidentified sp. 3980_1004	Basidiomycota	KY106002	276	236/279	85	0.011	0.003	0.007	-	-	-	0.005
Unidentified sp. 3980_1021	Ascomycota	KX192555	255	234/255	92	0.007	0.007	0.007	-	-	-	0.005
Unidentified sp. 3980_1044	Basidiomycota	KU188616	302	302/302	100	0.004	0.005	0.004	-	0.012	0.006	0.005
Unidentified sp. 3980_1047	Ascomycota	KF617397	237	234/239	98	-	-	-	0.008	0.024	0.016	0.005
Unidentified sp. 3980_1048	Ascomycota	KU062822	253	253/253	100	-	0.013	0.007	-	-	-	0.005
Ballistosporomyces sasicola	Basidiomycota	KY101699	298	294/298	99	-	-	-	0.028	0.004	0.016	0.005
Mucor luteus	Mucoromycota	KF876830	267	265/267	99	0.002	0.012	0.007	-	-	-	0.005
Sordariomycetes sp. 3980_1070	Ascomycota	KU702386	268	267/268	99	-	-	-	0.028	0.004	0.016	0.005
Phaeosphaeria sp. 3980_1083	Ascomycota	EU852369	245	244/245	99	0.002	0.012	0.007	-	-	-	0.005
Phellodon tomentosus	Basidiomycota	KC571767	337	336/337	99	0.009	0.005	0.007	-	-	-	0.005
Peltaster sp. 3980_1101	Ascomycota	HQ386245	255	255/255	100	-	0.013	0.007	-	-	-	0.005
Unidentified sp. 3980_1113	Ascomycota	KF527448	255	255/255	100	-	0.013	0.007	-	-	-	0.005
Unidentified sp. 3980_1125	Ascomycota	KP897465	254	254/254	100	-	-	-	0.024	0.008	0.016	0.005
Unidentified sp. 3980_1137	Ascomycota	HM069492	239	239/239	100	0.004	0.010	0.007	-	-	-	0.005
Rutstroemia firma	Ascomycota	LT158448	243	240/243	99	-	-	-	0.016	0.016	0.016	0.005
Unidentified sp. 3980_1174	Basidiomycota	KP897178	264	264/264	100	0.011	0.003	0.007	-	-	-	0.005
Unidentified sp. 3980_1184	Ascomycota	KX221368	237	236/237	99	-	-	-	0.028	0.004	0.016	0.005
Leotiomycetes sp. 3980_1191	Ascomycota	FR846475	242	242/242	100	0.007	0.007	0.007	-	-	-	0.005
Hormococcus conorum	Ascomycota	MH856727	248	247/248	99	-	-	-	0.012	0.020	0.016	0.005
Unidentified sp. 3980_1209	Ascomycota	DQ309130	239	239/239	100	0.007	0.002	0.004	-	0.012	0.006	0.005
Unidentified sp. 3980_1235	Ascomycota	JQ312745	237	234/238	98	0.011	0.003	0.007	-	-	-	0.005
Unidentified sp. 3980_1287	Mucoromycota	LC310483	359	35/35	100	-	-	-	0.004	0.028	0.016	0.005
Unidentified sp. 3980_1337	Basidiomycota	KC571714	289	271/309	88	-	0.012	0.006	-	0.004	0.002	0.005
Unidentified sp. 3980_1338	Ascomycota	KP891871	295	262/303	86	0.007	0.002	0.004	0.012	-	0.006	0.005
Unidentified sp. 3980_1376	Ascomycota	KX220817	274	274/274	100	-	-	-	-	0.032	0.016	0.005
Unidentified sp. 3980_1394	Ascomycota	MG827666	248	247/248	99	-	-	-	-	0.032	0.016	0.005
Unidentified sp. 3980_1422	Basidiomycota	DQ421143	311	308/311	99	-	-	-	-	0.032	0.016	0.005
Unidentified sp. 3980_1426	Basidiomycota	KP897264	296	296/297	99	-	0.013	0.007	-	-	-	0.005
Unidentified sp. 3980_1560	Ascomycota	GQ160168	216	201/216	93	-	-	-	0.032	-	0.016	0.005
Unidentified sp. 3980_1619	Ascomycota	MF976323	261	250/263	95	0.009	0.005	0.007	-	-	-	0.005

Unidentified sp. 3980_1727	Ascomycota	KF617845	267	261/267	98	-	-	-	0.032	-	0.016	0.005
Setomelanomma holmii	Ascomycota	MH863660	250	245/250	98	-	-	-	0.032	-	0.016	0.005
Unidentified sp. 3980_1961	Ascomycota	KU061770	242	242/244	99	0.007	0.007	0.007	-	-	-	0.005
Peniophora incarnata	Basidiomycota	KP814551	287	287/287	100	0.005	0.007	0.006	-	-	-	0.004
Unidentified sp. 3980_842	Basidiomycota	KU188551	271	270/271	99	0.005	0.007	0.006	-	-	-	0.004
Unidentified sp. 3980_844	Basidiomycota	MH452480	338	316/341	93	0.004	0.002	0.003	0.008	0.008	0.008	0.004
Unidentified sp. 3980_847	Basidiomycota	KM493615	304	304/304	100	-	-	-	-	0.028	0.014	0.004
Aspergillus domesticus	Ascomycota	KY087689	262	262/262	100	0.004	0.002	0.003	0.008	0.008	0.008	0.004
Glomus sp. 3980_866	Mucoromycota	MG429681	285	280/287	98	0.007	0.002	0.004	0.008	-	0.004	0.004
Unidentified sp. 3980_894	Basidiomycota	KP897644	265	264/265	99	-	-	-	0.012	0.016	0.014	0.004
Coprinopsis atramentaria	Basidiomycota	FN396110	306	306/306	100	0.011	0.002	0.006	-	-	-	0.004
Unidentified sp. 3980_915	Basidiomycota	KP890980	346	346/346	100	0.011	0.002	0.006	-	-	-	0.004
Cortinarius mucosus	Basidiomycota	FJ039581	296	296/296	100	0.002	0.003	0.003	0.004	0.012	0.008	0.004
Pleosporales sp. 3980_921	Ascomycota	MH450946	247	247/247	100	0.002	0.003	0.003	-	0.016	0.008	0.004
Agaricostilbales sp. 3980_927	Basidiomycota	KX403444	318	187/200	94	-	-	-	0.020	0.008	0.014	0.004
Boletus edulis	Basidiomycota	KP031595	400	398/400	99	0.007	0.005	0.006	-	-	-	0.004
Unidentified sp. 3980_934	Basidiomycota	HQ433145	284	283/284	99	0.002	0.002	0.002	0.004	0.016	0.010	0.004
Russula roseipes	Basidiomycota	MG679823	361	361/361	100	0.007	0.005	0.006	-	-	-	0.004
Unidentified sp. 3980_939	Ascomycota	KT219555	261	248/256	97	-	-	-	0.024	0.004	0.014	0.004
Cortinarius murinascens	Basidiomycota	KX355523	280	279/280	99	-	-	-	0.016	0.012	0.014	0.004
Clonostachys rosea	Ascomycota	MK036356	260	260/260	100	-	0.002	0.001	0.008	0.016	0.012	0.004
Unidentified sp. 3980_961	Basidiomycota	JQ313112	314	312/315	99	0.011	0.002	0.006	-	-	-	0.004
Unidentified sp. 3980_991	Ascomycota	KT219383	263	240/256	94	0.004	0.005	0.004	-	0.008	0.004	0.004
Bjerkandera adusta	Basidiomycota	MH857085	281	281/281	100	-	-	-	0.028	-	0.014	0.004
Unidentified sp. 3980_997	Ascomycota	KC222763	255	244/259	94	0.004	0.008	0.006	-	-	-	0.004
Unidentified sp. 3980_1035	Basidiomycota	MH451574	318	268/318	84	-	-	-	0.028	-	0.014	0.004
Unidentified sp. 3980_1036	Ascomycota	KP897646	265	264/265	99	0.004	0.003	0.003	0.008	0.004	0.006	0.004
Unidentified sp. 3980_1049	Basidiomycota	LC277042	348	348/348	100	0.007	0.005	0.006	-	-	-	0.004
Phaeotheca fissurella	Ascomycota	MH864116	238	238/238	100	0.002	0.010	0.006	-	-	-	0.004
Unidentified sp. 3980_1065	Basidiomycota	KU061614	272	272/273	99	-	-	-	-	0.028	0.014	0.004
Unidentified sp. 3980_1078	Basidiomycota	GU452516	272	265/272	97	0.005	0.007	0.006	-	-	-	0.004
Unidentified sp. 3980_1079	Mucoromycota	LT608035	344	344/344	100	-	0.002	0.001	-	0.024	0.012	0.004
Unidentified sp. 3980_1084	Ascomycota	MG827507	246	246/246	100	0.005	0.007	0.006	-	-	-	0.004
Unidentified sp. 3980_1093	Ascomycota	KP897196	254	254/254	100	-	-	-	-	0.028	0.014	0.004
Peniophora pini	Basidiomycota	MG547963	287	287/287	100	-	-	-	-	0.028	0.014	0.004
Unidentified sp. 3980_1138	Basidiomycota	KX221225	327	327/327	100	-	-	-	0.024	0.004	0.014	0.004
Unidentified sp. 3980_1143	Ascomycota	KT220108	247	247/247	100	0.005	0.005	0.005	0.004	-	0.002	0.004
Unidentified sp. 3980_1153	Basidiomycota	KY006611	243	243/243	100	0.013	-	0.006	-	-	-	0.004
Unidentified sp. 3980_1157	Ascomycota	MG827536	250	250/250	100	-	-	-	0.008	0.020	0.014	0.004
Unidentified sp. 3980_1158	Ascomycota	KP897650	257	248/261	95	0.005	0.005	0.005	0.004	-	0.002	0.004
Unidentified sp. 3980_1160	Ascomycota	KP897425	303	298/308	97	0.005	0.007	0.006	-	-	-	0.004
Unidentified sp. 3980_1162	Basidiomycota	KY006608	309	309/309	100	0.007	0.005	0.006	-	-	-	0.004
Vascellum intermedium	Basidiomycota	DQ112556	306	306/306	100	0.004	0.008	0.006	-	-	-	0.004
Unidentified sp. 3980_1197	Ascomycota	KF617235	237	234/237	99	0.005	0.005	0.005	-	0.004	0.002	0.004
Unidentified sp. 3980_1201	Ascomycota	HQ433050	264	226/271	83	0.011	0.002	0.006	-	-	-	0.004
Unidentified sp. 3980_1207	Ascomycota	KP887943	258	258/258	100	0.004	0.008	0.006	-	-	-	0.004

Unidentified sp. 3980_1224	Ascomycota	KP891502	252	251/252	99	-	0.010	0.005	0.004	-	0.002	0.004
Unidentified sp. 3980_1244	Ascomycota	KU839402	242	242/242	100	0.011	0.002	0.006	-	-	-	0.004
Unidentified sp. 3980_1248	Ascomycota	KP891693	241	240/241	99	-	-	-	0.008	0.020	0.014	0.004
Unidentified sp. 3980_1251	Ascomycota	KP897764	320	319/320	99	0.007	0.005	0.006	-	-	-	0.004
Unidentified sp. 3980_1254	Basidiomycota	KP892240	299	259/301	86	-	-	-	0.012	0.016	0.014	0.004
Herpotrichiellaceae sp. 3980_1256	Ascomycota	GQ223468	288	282/282	100	0.002	0.008	0.005	0.004	-	0.002	0.004
Unidentified sp. 3980_1270	Ascomycota	KC965730	271	270/271	99	-	0.012	0.006	-	-	-	0.004
Unidentified sp. 3980_1297	Ascomycota	FN298730	278	277/279	99	0.005	-	0.003	0.012	0.004	0.008	0.004
Unidentified sp. 3980_1299	Ascomycota	MF569216	262	261/262	99	0.002	-	0.001	0.024	-	0.012	0.004
Richoniella sp. 3980_1324	Basidiomycota	KP191912	298	282/295	96	0.009	0.003	0.006	-	-	-	0.004
Saccharomycetes sp. 3980_1335	Ascomycota	FJ553927	216	216/216	100	0.005	0.007	0.006	-	-	-	0.004
Unidentified sp. 3980_1343	Basidiomycota	KY687531	337	329/345	95	0.011	0.002	0.006	-	-	-	0.004
Unidentified sp. 3980_1350	Ascomycota	KU061304	235	232/235	99	-	-	-	0.028	-	0.014	0.004
Unidentified sp. 3980_1356	Ascomycota	KT219565	202	199/202	99	-	-	-	-	0.028	0.014	0.004
Unidentified sp. 3980_1402	Basidiomycota	KP892469	204	198/207	96	-	-	-	0.012	0.016	0.014	0.004
Trametes hirsuta	Basidiomycota	MH860685	285	284/285	99	-	-	-	0.004	0.024	0.014	0.004
Unidentified sp. 3980_1470	Ascomycota	KX776884	254	250/250	100	-	-	-	0.012	0.016	0.014	0.004
Unidentified sp. 3980_1521	Ascomycota	KP897311	236	236/236	100	-	-	-	0.004	0.024	0.014	0.004
Unidentified sp. 3980_1633	Chytridiomycota	KP888041	333	333/333	100	-	-	-	0.016	0.012	0.014	0.004
Inocybe sp. 3980_1865	Basidiomycota	KX867465	263	263/263	100	0.011	-	0.005	0.004	-	0.002	0.004
Unidentified sp. 3980_1932	Ascomycota	KU063314	244	243/245	99	-	-	-	0.012	0.016	0.014	0.004
Rhodotorula sp. 3980_2145	Basidiomycota	MK493334	301	300/301	99	0.002	0.010	0.006	-	-	-	0.004
Unidentified sp. 3980_868	Ascomycota	KP898098	282	268/285	94	0.009	0.002	0.005	-	-	-	0.004
Unidentified sp. 3980_887	Basidiomycota	JQ312970	291	288/295	98	0.002	0.008	0.005	-	-	-	0.004
Tremella sp. 3980_888	Basidiomycota	AF042425	239	233/239	97	-	-	-	0.012	0.012	0.012	0.004
Unidentified sp. 3980_896	Ascomycota	KU062830	247	229/250	92	0.005	0.005	0.005	-	-	-	0.004
Unidentified sp. 3980_902	Ascomycota	KP897663	268	265/268	99	-	0.002	0.001	-	0.020	0.010	0.004
Cantharellus cibarius	Basidiomycota	DQ200926	442	442/442	100	0.002	0.008	0.005	-	-	-	0.004
Hebeloma crustuliniforme	Basidiomycota	MH931275	310	310/310	100	0.002	0.008	0.005	-	-	-	0.004
Unidentified sp. 3980_974	Ascomycota	MF976122	241	240/241	99	0.004	-	0.002	0.012	0.004	0.008	0.004
Unidentified sp. 3980_986	Ascomycota	JQ312774	243	243/243	100	0.005	0.005	0.005	-	-	-	0.004
Unidentified sp. 3980_988	Basidiomycota	KX220798	297	294/297	99	0.004	-	0.002	-	0.016	0.008	0.004
Peniophora simulans	Basidiomycota	MH861850	287	285/287	99	-	0.010	0.005	-	-	-	0.004
Unidentified sp. 3980_1009	Ascomycota	MG207157	240	234/240	98	0.009	0.002	0.005	-	-	-	0.004
Unidentified sp. 3980_1011	Chytridiomycota	JN943807	320	298/322	93	-	-	-	-	0.024	0.012	0.004
Unidentified sp. 3980_1017	Basidiomycota	AM902046	338	337/338	99	0.005	0.003	0.004	0.004	-	0.002	0.004
Hypholoma sp. 3980_1025	Basidiomycota	KC176333	304	302/304	99	-	0.010	0.005	-	-	-	0.004
Unidentified sp. 3980_1030	Ascomycota	KF617402	265	264/266	99	-	0.010	0.005	-	-	-	0.004
Unidentified sp. 3980_1033	Ascomycota	JQ666458	270	268/270	99	0.005	0.005	0.005	-	-	-	0.004
Cantharellales sp. 3980_1034	Basidiomycota	MF483456	318	317/318	99	-	-	-	0.024	-	0.012	0.004
Unidentified sp. 3980_1053	Ascomycota	AY541272	259	218/264	83	0.004	0.007	0.005	-	-	-	0.004
Unidentified sp. 3980_1066	Cryptomycota	MG161259	204	189/204	93	0.004	0.007	0.005	-	-	-	0.004
Unidentified sp. 3980_1067	Basidiomycota	KP406574	325	286/343	83	-	-	-	0.016	0.008	0.012	0.004
Unidentified sp. 3980_1069	Basidiomycota	MF484636	288	269/296	91	-	-	-	-	0.024	0.012	0.004
Phlebia radiata	Basidiomycota	MH857641	287	287/287	100	-	-	-	0.024	-	0.012	0.004
Agaricales sp. 3980_1088	Basidiomycota	DQ182453	298	292/299	98	0.002	0.008	0.005	-	-	-	0.004

Mortierellomycetes sp. 3980_1114	Mucoromycota	MH451539	342	342/342	100	0.004	-	0.002	0.012	0.004	0.008	0.004
Helotiales sp. 3980_1122	Ascomycota	MF487485	208	208/208	100	0.004	0.007	0.005	-	-	-	0.004
Helotiales sp. 3980_1126	Ascomycota	MF615017	239	239/239	100	0.002	0.008	0.005	-	-	-	0.004
Unidentified sp. 3980_1132	Basidiomycota	KX196116	348	323/351	92	-	-	-	-	0.024	0.012	0.004
Unidentified sp. 3980_1134	Basidiomycota	KP891331	311	307/311	99	0.002	0.007	0.004	-	-	-	0.003
Unidentified sp. 3980_1136	Mucoromycota	KP897541	265	249/269	93	-	-	-	0.012	0.012	0.012	0.004
Unidentified sp. 3980_1140	Basidiomycota	KM494140	231	228/231	99	0.011	-	0.005	-	-	-	0.004
Sebacinales sp. 3980_1146	Basidiomycota	FJ475814	305	305/305	100	0.007	0.003	0.005	-	-	-	0.004
Pholiota spumosa	Basidiomycota	MH856605	306	306/306	100	0.004	0.007	0.005	-	-	-	0.004
Unidentified sp. 3980_1193	Ascomycota	KP891582	205	199/205	97	0.002	0.008	0.005	-	-	-	0.004
Unidentified sp. 3980_1196	Basidiomycota	KU061893	277	276/277	99	0.009	0.002	0.005	-	-	-	0.004
Bannoa sp. 3980_1200	Basidiomycota	KY101704	306	298/308	97	-	-	-	-	0.024	0.012	0.004
Sordariomycetes sp. 3980_1203	Ascomycota	MH451985	244	244/244	100	-	-	-	0.020	0.004	0.012	0.004
Unidentified sp. 3980_1229	Ascomycota	KP888103	235	217/235	92	0.009	0.002	0.005	-	-	-	0.004
Unidentified sp. 3980_1240	Basidiomycota	KP891606	322	320/322	99	0.004	0.007	0.005	-	-	-	0.004
Unidentified sp. 3980_1262	Ascomycota	MF486549	325	299/330	91	-	-	-	0.004	0.020	0.012	0.004
Unidentified sp. 3980_1269	Ascomycota	KP897350	250	250/250	100	-	0.002	0.001	0.008	0.012	0.010	0.004
Unidentified sp. 3980_1283	Basidiomycota	KU063014	305	286/287	99	0.007	0.003	0.005	-	-	-	0.004
Unidentified sp. 3980_1284	Ascomycota	MF570804	360	360/361	99	0.005	0.003	0.004	0.004	-	0.002	0.004
Unidentified sp. 3980_1289	Ascomycota	MF486799	242	242/242	100	0.004	0.003	0.003	-	0.008	0.004	0.004
Unidentified sp. 3980_1292	Ascomycota	MG827585	244	244/244	100	-	0.010	0.005	-	-	-	0.004
Unidentified sp. 3980_1304	Mucoromycota	GU315929	289	245/252	97	0.005	0.005	0.005	-	-	-	0.004
Unidentified sp. 3980_1312	Ascomycota	MG827600	243	243/243	100	-	-	-	0.020	0.004	0.012	0.004
Tomentella sp. 3980_1348	Basidiomycota	MF352799	317	317/317	100	-	-	-	0.020	0.004	0.012	0.004
Pholiota lenta	Basidiomycota	JF908582	300	300/300	100	-	-	-	0.024	-	0.012	0.004
Phellodon melaleucus	Basidiomycota	MH118171	299	299/299	100	0.007	-	0.003	0.004	0.004	0.004	0.004
Lachnum sp. 3980_1458	Ascomycota	AB481283	239	231/239	97	0.002	0.008	0.005	-	-	-	0.004
Unidentified sp. 3980_1466	Ascomycota	KP889888	231	231/231	100	0.005	0.005	0.005	-	-	-	0.004
Unidentified sp. 3980_1499	Ascomycota	FJ820723	237	226/237	95	0.007	0.002	0.004	0.004	-	0.002	0.004
Unidentified sp. 3980_1518	Ascomycota	KP897946	274	218/274	80	0.004	0.005	0.004	-	0.004	0.002	0.004
Unidentified sp. 3980_1527	Ascomycota	MF569641	243	243/243	100	-	-	-	0.012	0.012	0.012	0.004
Unidentified sp. 3980_1625	Basidiomycota	KX193431	314	296/318	93	0.002	0.007	0.004	-	0.004	0.002	0.004
Unidentified sp. 3980_1641	Mucoromycota	MF483707	284	36/36	100	-	-	-	0.012	0.012	0.012	0.004
Unidentified sp. 3980_1909	Ascomycota	KT196052	242	240/242	99	-	0.010	0.005	-	-	-	0.004
Unidentified sp. 3980_1911	Ascomycota	HM239764	250	248/251	99	0.011	-	0.005	-	-	-	0.004
Unidentified sp. 3980_1956	Basidiomycota	KM493594	301	280/303	92	0.007	-	0.003	0.004	0.004	0.004	0.004
Helotiales sp. 3980_1977	Ascomycota	KJ826706	237	234/237	99	0.007	0.003	0.005	-	-	-	0.004
Unidentified sp. 3980_2182	Basidiomycota	KT241667	233	214/239	90	0.007	0.002	0.004	0.004	-	0.002	0.004
Eleutheromyces sp. 3980_2297	Ascomycota	KJ710471	243	234/243	96	0.011	-	0.005	-	-	-	0.004
Unidentified sp. 3980_4071	Ascomycota	KU838300	259	259/259	100	0.004	-	0.002	-	0.016	0.008	0.004
Unidentified sp. 3980_5024	Basidiomycota	KC965576	275	235/281	84	-	-	-	0.024	-	0.012	0.004
Unidentified sp. 3980_955	Ascomycota	MG827687	263	262/263	99	0.005	0.003	0.004	-	-	-	0.003
Unidentified sp. 3980_968	Basidiomycota	KP892240	287	281/290	97	-	0.008	0.004	-	-	-	0.003
Plectosphaerella cucumerina	Ascomycota	MK079567	261	261/263	99	-	-	-	-	0.020	0.010	0.003
Tomentella sp. 3980_971	Basidiomycota	KM403022	314	312/314	99	0.007	0.002	0.004	-	-	-	0.003
Unidentified sp. 3980_983	Basidiomycota	HM037684	298	297/298	99	0.002	-	0.001	-	0.016	0.008	0.003

Unidentified sp. 3980_985	Ascomycota	LT608021	338	337/338	99	-	-	-	0.004	0.016	0.010	0.003
Galerina marginata	Basidiomycota	KX236118	307	307/307	100	-	-	-	-	0.020	0.010	0.003
Unidentified sp. 3980_998	Mucoromycota	KX222258	282	274/282	97	0.005	0.002	0.003	0.004	-	0.002	0.003
Unidentified sp. 3980_999	Ascomycota	KP891972	266	265/266	99	0.002	0.007	0.004	-	-	-	0.003
Lophiotrema vagabundum	Ascomycota	LC194499	252	252/252	100	0.004	0.005	0.004	-	-	-	0.003
Coleosporium euphrasiae	Basidiomycota	KY810469	639	341/342	99	0.004	0.005	0.004	-	-	-	0.003
Unidentified sp. 3980_1022	Ascomycota	KP891832	268	268/268	100	0.002	0.007	0.004	-	-	-	0.003
Unidentified sp. 3980_1032	Ascomycota	MG827432	241	240/241	99	0.005	0.002	0.003	0.004	-	0.002	0.003
Unidentified sp. 3980_1054	Basidiomycota	AB476528	290	275/299	92	-	0.002	0.001	0.008	0.008	0.008	0.003
Unidentified sp. 3980_1061	Ascomycota	EF373580	256	250/258	97	-	0.002	0.001	0.004	0.012	0.008	0.003
Unidentified sp. 3980_1064	Basidiomycota	KM493585	308	304/308	99	-	-	-	0.020	-	0.010	0.003
Leratiomyces sp. 3980_1076	Basidiomycota	MH043621	302	302/302	100	0.005	0.003	0.004	-	-	-	0.003
Unidentified sp. 3980_1081	Ascomycota	MF570965	318	311/319	97	0.005	0.003	0.004	-	-	-	0.003
Lepista nuda	Basidiomycota	KX449413	303	303/303	100	0.004	0.005	0.004	-	-	-	0.003
Inocybe soluta	Basidiomycota	HQ604553	290	288/290	99	0.004	0.005	0.004	-	-	-	0.003
Unidentified sp. 3980_1086	Ascomycota	KP891276	241	240/241	99	0.005	0.002	0.003	-	0.004	0.002	0.003
Unidentified sp. 3980_1105	Basidiomycota	KX194538	252	251/252	99	0.004	0.005	0.004	-	-	-	0.003
Fusarium sp. 3980_1108	Ascomycota	EU860075	257	255/255	100	-	0.008	0.004	-	-	-	0.003
Unidentified sp. 3980_1109	Basidiomycota	MG231611	219	62/68	91	0.005	0.003	0.004	-	-	-	0.003
Cenococcum sp. 3980_1111	Ascomycota	KX844654	239	239/239	100	-	-	-	-	0.020	0.010	0.003
Unidentified sp. 3980_1112	Basidiomycota	KY104505	272	245/277	88	0.002	0.007	0.004	-	-	-	0.003
Unidentified sp. 3980_1119	Ascomycota	KT957806	255	252/255	99	0.002	0.003	0.003	0.008	-	0.004	0.003
Unidentified sp. 3980_1120	Basidiomycota	KT334768	305	305/305	100	-	-	-	0.008	0.012	0.010	0.003
Unidentified sp. 3980_1124	Cryptomycota	FJ475780	225	225/225	100	0.004	0.003	0.003	0.004	-	0.002	0.003
Bryochiton sp. 3980_1128	Ascomycota	KM186836	246	213/224	95	0.002	-	0.001	-	0.016	0.008	0.003
Unidentified sp. 3980_1133	Basidiomycota	KX816316	315	315/315	100	0.002	0.007	0.004	-	-	-	0.003
Unidentified sp. 3980_1139	Ascomycota	MF570233	274	264/274	96	0.004	0.005	0.004	-	-	-	0.003
Umbelopsis ramanniana	Mucoromycota	GU934569	314	312/314	99	-	-	-	0.020	-	0.010	0.003
Unidentified sp. 3980_1161	Basidiomycota	KY810469	631	339/343	99	0.004	-	0.002	0.008	0.004	0.006	0.003
Unidentified sp. 3980_1165	Basidiomycota	KP897584	284	248/291	85	-	-	-	0.008	0.012	0.010	0.003
Fellomyces sp. 3980_1177	Basidiomycota	AJ608670	275	260/276	94	-	-	-	-	0.020	0.010	0.003
Unidentified sp. 3980_1180	Ascomycota	MF976615	238	219/239	92	0.005	0.003	0.004	-	-	-	0.003
Unidentified sp. 3980_1181	Ascomycota	MH864071	248	220/250	88	-	-	-	0.020	-	0.010	0.003
Unidentified sp. 3980_1183	Basidiomycota	JX998756	288	280/288	97	-	-	-	-	0.020	0.010	0.003
Unidentified sp. 3980_1187	Ascomycota	KX194194	235	234/235	99	-	0.008	0.004	-	-	-	0.003
Unidentified sp. 3980_1190	Ascomycota	KP891184	237	237/238	99	-	-	-	0.012	0.008	0.010	0.003
Unidentified sp. 3980_1217	Ascomycota	MF570274	240	240/240	100	-	-	-	0.016	0.004	0.010	0.003
Unidentified sp. 3980_1223	Ascomycota	MG827607	251	251/251	100	0.009	-	0.004	-	-	-	0.003
Unidentified sp. 3980_1234	Chytridiomycota	KU064088	319	318/324	98	0.009	-	0.004	-	-	-	0.003
Penicillium sp. 3980_1247	Ascomycota	KF932962	254	249/256	97	-	-	-	0.020	-	0.010	0.003
Unidentified sp. 3980_1257	Basidiomycota	KX196116	304	46/51	90	-	0.002	0.001	-	0.016	0.008	0.003
Unidentified sp. 3980_1260	Basidiomycota	KX816144	301	289/303	95	-	0.008	0.004	-	-	-	0.003
Unidentified sp. 3980_1263	Ascomycota	KP897614	254	253/254	99	0.005	0.003	0.004	-	-	-	0.003
Unidentified sp. 3980_1275	Ascomycota	KU582138	236	236/236	100	0.002	0.003	0.003	0.008	-	0.004	0.003
Unidentified sp. 3980_1277	Basidiomycota	KP891581	291	291/291	100	0.002	0.007	0.004	-	-	-	0.003
Unidentified sp. 3980_1278	Ascomycota	KR266841	244	242/244	100	0.005	0.003	0.004	-	-	-	0.003

Unidentified sp. 3980_1280	Basidiomycota	MG828377	245	244/247	99	0.007	0.002	0.004	-	-	-	0.003
Unidentified sp. 3980_1306	Ascomycota	MG827906	242	226/244	93	-	-	-	0.004	0.016	0.010	0.003
Entoloma sericeum	Basidiomycota	KY706153	301	301/301	100	-	-	-	-	0.020	0.010	0.003
Acremonium furcatum	Ascomycota	MH859660	266	266/266	100	-	0.008	0.004	-	-	-	0.003
Cantharellales sp. 3980_1332	Basidiomycota	HQ022036	300	296/300	99	-	0.008	0.004	-	-	-	0.003
Unidentified sp. 3980_1333	Ascomycota	KC222735	273	264/274	96	0.009	-	0.004	-	-	-	0.003
Unidentified sp. 3980_1358	Basidiomycota	AB476528	299	298/299	99	0.007	0.002	0.004	-	-	-	0.003
Entoloma conferendum	Basidiomycota	MF977971	296	296/296	100	-	-	-	0.020	-	0.010	0.003
Unidentified sp. 3980_1370	Basidiomycota	KP897721	300	297/304	98	-	0.008	0.004	-	-	-	0.003
Unidentified sp. 3980_1388	Basidiomycota	AM697935	345	342/345	99	0.009	-	0.004	-	-	-	0.003
Unidentified sp. 3980_1397	Ascomycota	MF571117	254	230/259	89	0.004	0.003	0.003	0.004	-	0.002	0.003
Unidentified sp. 3980_1400	Basidiomycota	JF461322	223	57/61	93	0.007	0.002	0.004	-	-	-	0.003
Unidentified sp. 3980_1403	Basidiomycota	KX223017	326	284/328	87	-	-	-	0.016	0.004	0.010	0.003
Unidentified sp. 3980_1416	Ascomycota	MG827423	240	240/240	100	-	-	-	0.008	0.012	0.010	0.003
Helotiales sp. 3980_1421	Ascomycota	KJ826609	242	237/244	97	-	-	-	0.016	0.004	0.010	0.003
Coleosporium euphrasiae	Basidiomycota	KY810469	642	337/341	99	-	-	-	0.020	-	0.010	0.003
Unidentified sp. 3980_1432	Basidiomycota	JF300840	315	315/315	100	-	-	-	0.008	0.012	0.010	0.003
Chlorophyllum olivieri	Basidiomycota	MH979435	292	291/291	100	0.004	0.005	0.004	-	-	-	0.003
Unidentified sp. 3980_1442	Ascomycota	MG827676	246	237/247	96	-	-	-	-	0.020	0.010	0.003
Unidentified sp. 3980_1443	Basidiomycota	KM493387	286	282/287	98	0.007	0.002	0.004	-	-	-	0.003
Unidentified sp. 3980_1455	Ascomycota	MG206673	252	251/253	99	-	-	-	0.012	0.008	0.010	0.003
Unidentified sp. 3980_1473	Ascomycota	KX192643	268	225/277	81	-	-	-	0.016	0.004	0.010	0.003
Cosmospora berkeleyana	Ascomycota	MH859041	253	253/253	100	0.009	-	0.004	-	-	-	0.003
Tricholoma cingulatum	Basidiomycota	KJ705244	298	296/299	99	-	-	-	0.004	0.016	0.010	0.003
Unidentified sp. 3980_1557	Ascomycota	AM901935	265	265/265	100	-	-	-	0.016	0.004	0.010	0.003
Unidentified sp. 3980_1566	Basidiomycota	MG207070	252	252/252	100	0.005	0.003	0.004	-	-	-	0.003
Metarhizium anisopliae	Ascomycota	HQ380866	270	270/270	100	0.009	-	0.004	-	-	-	0.003
Unidentified sp. 3980_1600	Ascomycota	KP897174	249	249/249	100	-	-	-	0.008	0.012	0.010	0.003
Diplodia sp. 3980_1616	Ascomycota	MH865376	253	253/253	100	-	-	-	0.020	-	0.010	0.003
Unidentified sp. 3980_1649	Ascomycota	MH451552	268	268/268	100	-	-	-	0.012	0.008	0.010	0.003
Fusarium oxysporum	Ascomycota	MH455293	244	244/244	100	-	0.008	0.004	-	-	-	0.003
Unidentified sp. 3980_1659	Ascomycota	KP897213	295	295/297	99	-	0.008	0.004	-	-	-	0.003
Amphinema sp. 3980_1666	Basidiomycota	MK285739	272	272/272	100	-	-	-	0.012	0.008	0.010	0.003
Meyerozyma guilliermondii	Ascomycota	MK243634	286	286/286	100	-	-	-	0.020	-	0.010	0.003
Unidentified sp. 3980_1747	Ascomycota	KP897539	271	271/271	100	0.005	-	0.003	-	0.008	0.004	0.003
Unidentified sp. 3980_1778	Ascomycota	KP892199	264	263/264	99	-	-	-	-	0.020	0.010	0.003
Hypoxylon howeanum	Ascomycota	MH859700	256	256/258	99	-	-	-	0.004	-	0.002	0.001
Unidentified sp. 3980_1981	Ascomycota	MG828088	246	246/246	100	-	-	-	0.020	-	0.010	0.003
Unidentified sp. 3980_2020	Basidiomycota	MG827893	285	285/285	100	0.002	0.007	0.004	-	-	-	0.003
Unidentified sp. 3980_2021	Basidiomycota	KX195904	216	199/218	91	0.009	-	0.004	-	-	-	0.003
Unidentified sp. 3980_2045	Ascomycota	MG206602	241	228/242	94	0.009	-	0.004	-	-	-	0.003
Unidentified sp. 3980_2132	Ascomycota	LC096379	243	242/243	99	0.002	0.007	0.004	-	-	-	0.003
Unidentified sp. 3980_2164	Ascomycota	KT155796	290	78/84	93	-	-	-	0.020	-	0.010	0.003
Unidentified sp. 3980_2176	Basidiomycota	LT608066	277	277/277	100	0.004	0.005	0.004	-	-	-	0.003
Unidentified sp. 3980_2274	Ascomycota	KP889538	240	231/240	96	-	0.005	0.003	-	0.008	0.004	0.003
Unidentified sp. 3980_2332	Ascomycota	KP897238	257	257/257	100	-	0.002	0.001	-	0.016	0.008	0.003

Unidentified sp. 3980_3217	Ascomycota	MG827696	253	253/253	100	0.009	-	0.004	-	-	-	0.003
Unidentified sp. 3980_5580	Ascomycota	KX195953	253	251/253	99	-	-	-	0.012	0.008	0.010	0.003
Unidentified sp. 3980_1099	Basidiomycota	GQ999405	321	318/322	99	0.005	0.002	0.003	-	-	-	0.002
Cortinarius populinus	Basidiomycota	AY669521	291	289/291	99	0.002	0.002	0.002	0.008	-	0.004	0.002
Unidentified sp. 3980_1102	Mucoromycota	KC965558	213	210/215	98	0.004	0.003	0.003	-	-	-	0.002
Unidentified sp. 3980_1116	Ascomycota	KU063507	324	61/62	98	0.007	-	0.003	-	-	-	0.002
Unidentified sp. 3980_1131	Ascomycota	KP891156	246	244/246	99	-	-	-	-	0.016	0.008	0.002
Unidentified sp. 3980_1135	Ascomycota	KJ827181	236	236/236	100	0.004	0.003	0.003	-	-	-	0.002
Unidentified sp. 3980_1147	Basidiomycota	KR698874	299	297/299	99	0.005	-	0.003	-	0.004	0.002	0.002
Unidentified sp. 3980_1159	Ascomycota	KM494175	521	65/70	93	0.004	0.003	0.003	-	-	-	0.002
Unidentified sp. 3980_1170	Ascomycota	KP891382	234	233/234	99	-	0.002	0.001	0.004	0.008	0.006	0.002
Unidentified sp. 3980_1210	Ascomycota	KU063709	280	218/288	76	0.007	-	0.003	-	-	-	0.002
Unidentified sp. 3980_1211	Basidiomycota	KT585733	298	293/299	98	-	0.002	0.001	0.012	-	0.006	0.002
Cutaneotrichosporon moniliiforme	Basidiomycota	KY558357	259	258/259	99	-	0.005	0.003	-	-	-	0.002
Unidentified sp. 3980_1221	Mucoromycota	KX222715	362	360/362	99	-	0.007	0.003	-	-	-	0.002
Unidentified sp. 3980_1233	Ascomycota	KP897593	239	236/240	98	0.007	-	0.003	-	-	-	0.002
Unidentified sp. 3980_1243	Ascomycota	KT197511	249	244/250	98	0.004	0.003	0.003	-	-	-	0.002
Unidentified sp. 3980_1249	Basidiomycota	MG827975	277	276/277	99	-	-	-	0.004	0.012	0.008	0.002
Cortinarius fulvescens	Basidiomycota	KX388634	298	298/299	99	-	-	-	0.016	-	0.008	0.002
Unidentified sp. 3980_1258	Ascomycota	KF296849	241	236/241	98	-	0.007	0.003	-	-	-	0.002
Unidentified sp. 3980_1261	Ascomycota	MF570081	254	253/254	99	-	-	-	0.004	0.012	0.008	0.002
Unidentified sp. 3980_1268	Mucoromycota	KX222515	353	328/364	90	0.002	0.005	0.003	-	-	-	0.002
Cortinarius saniosus	Basidiomycota	KX355519	292	291/292	99	0.004	0.003	0.003	-	-	-	0.002
Unidentified sp. 3980_1276	Ascomycota	MG827440	238	237/238	99	0.002	-	0.001	-	-	-	0.001
Unidentified sp. 3980_1282	Basidiomycota	KX220524	279	277/279	99	0.005	-	0.003	0.004	-	0.002	0.002
Cortinarius imbutus	Basidiomycota	KX964507	272	271/272	99	-	-	-	0.016	-	0.008	0.002
Unidentified sp. 3980_1295	Ascomycota	KF297116	337	282/339	83	0.005	0.002	0.003	-	-	-	0.002
Unidentified sp. 3980_1296	Ascomycota	KP898051	248	247/249	99	0.002	0.005	0.003	-	-	-	0.002
Unidentified sp. 3980_1302	Ascomycota	GU211937	310	280/320	88	0.005	0.002	0.003	-	-	-	0.002
Cortinarius heterodepressus	Basidiomycota	MG136830	292	292/292	100	-	0.007	0.003	-	-	-	0.002
Coleosporium euphrasiae	Basidiomycota	KY810469	637	338/341	99	-	-	-	0.004	0.012	0.008	0.002
Unidentified sp. 3980_1307	Mucoromycota	KX193375	343	343/343	100	-	0.002	0.001	-	0.012	0.006	0.002
Helotiales sp. 3980_1308	Ascomycota	KJ827044	242	233/242	96	0.002	-	0.001	0.004	0.008	0.006	0.002
Unidentified sp. 3980_1313	Basidiomycota	MG827897	299	299/299	100	-	-	-	-	0.016	0.008	0.002
Leccinum versipelle	Basidiomycota	AY538831	412	412/412	100	0.002	0.005	0.003	-	-	-	0.002
Unidentified sp. 3980_1325	Ascomycota	KX194029	277	276/277	99	0.002	-	0.001	-	0.012	0.006	0.002
Agaricus sp. 3980_1328	Basidiomycota	KJ859122	306	305/306	99	0.007	-	0.003	-	-	-	0.002
Unidentified sp. 3980_1330	Basidiomycota	MG231611	221	62/68	91	0.002	0.003	0.003	0.004	-	0.002	0.002
Unidentified sp. 3980_1339	Ascomycota	KU063063	241	240/242	99	-	-	-	0.008	0.008	0.008	0.002
Archaeospora sp. 3980_1341	Mucoromycota	AM420405	281	274/284	96	-	-	-	0.008	0.008	0.008	0.002
Unidentified sp. 3980_1353	Ascomycota	KU063688	242	237/242	98	0.004	0.003	0.003	-	-	-	0.002
Unidentified sp. 3980_1359	Ascomycota	KM493655	238	238/238	100	0.007	-	0.003	-	-	-	0.002
Gymnopilus decipiens	Basidiomycota	MF039256	306	306/306	100	0.002	0.005	0.003	-	-	-	0.002
Unidentified sp. 3980_1379	Basidiomycota	KM493850	315	312/317	98	-	-	-	-	0.016	0.008	0.002
Unidentified sp. 3980_1384	Cryptomycota	KT219470	224	220/226	97	-	-	-	-	0.016	0.008	0.002
Heterocephalacria sp. 3980_1385	Basidiomycota	MK307714	323	322/323	99	-	-	-	0.016	-	0.008	0.002

Unidentified sp. 3980_1393	Basidiomycota	JX489811	273	233/291	80	-	0.002	0.001	-	0.012	0.006	0.002
Lophodermium seditiosum	Ascomycota	KY742560	833	286/287	99	0.004	0.003	0.003	-	-	-	0.002
Unidentified sp. 3980_1414	Ascomycota	KP887826	243	242/243	99	-	-	-	0.012	0.004	0.008	0.002
Unidentified sp. 3980_1417	Ascomycota	KU063079	238	236/241	98	-	-	-	0.016	-	0.008	0.002
Unidentified sp. 3980_1419	Ascomycota	KU061991	250	241/251	96	0.002	0.002	0.002	0.004	0.004	0.004	0.002
Ilyonectria panacis	Ascomycota	MH865176	257	257/257	100	-	0.003	0.002	0.008	-	0.004	0.002
Unidentified sp. 3980_1439	Ascomycota	MG827779	245	239/245	98	-	-	-	0.008	0.008	0.008	0.002
Unidentified sp. 3980_1441	Ascomycota	KX193508	281	267/287	93	0.004	0.003	0.003	-	-	-	0.002
Piloderma olivaceum	Basidiomycota	MH864982	281	281/281	100	0.002	-	0.001	-	0.012	0.006	0.002
Unidentified sp. 3980_1464	Ascomycota	KP891033	240	226/243	93	0.005	0.002	0.003	-	-	-	0.002
Unidentified sp. 3980_1465	Basidiomycota	JF461322	221	57/61	93	-	0.007	0.003	-	-	-	0.002
Unidentified sp. 3980_1469	Ascomycota	MF971476	255	255/255	100	0.005	0.002	0.003	-	-	-	0.002
Unidentified sp. 3980_1475	Basidiomycota	KP892390	309	292/309	94	-	-	-	0.016	-	0.008	0.002
Unidentified sp. 3980_1484	Basidiomycota	KP892240	298	254/300	85	-	-	-	-	0.016	0.008	0.002
Unidentified sp. 3980_1485	Basidiomycota	FJ820536	299	299/299	100	0.004	0.003	0.003	-	-	-	0.002
Mycena pelianthina	Basidiomycota	JF908379	310	306/311	98	0.005	0.002	0.003	-	-	-	0.002
Unidentified sp. 3980_1531	Ascomycota	KP897394	259	223/261	85	0.002	0.005	0.003	-	-	-	0.002
Unidentified sp. 3980_1536	Ascomycota	KP889341	256	253/257	98	0.005	0.002	0.003	-	-	-	0.002
Unidentified sp. 3980_1540	Ascomycota	JQ312717	236	236/236	100	-	-	-	-	0.016	0.008	0.002
Pholiota flammans	Basidiomycota	AF195601	290	285/290	98	0.007	-	0.003	-	-	-	0.002
Unidentified sp. 3980_1542	Ascomycota	MF569864	240	216/244	89	-	-	-	-	0.016	0.008	0.002
Unidentified sp. 3980_1545	Ascomycota	KP889330	250	243/251	97	-	-	-	0.012	0.004	0.008	0.002
Unidentified sp. 3980_1548	Ascomycota	KX777095	243	240/241	99	-	0.007	0.003	-	-	-	0.002
Unidentified sp. 3980_1550	Ascomycota	MF569674	346	346/346	100	0.005	0.002	0.003	-	-	-	0.002
Unidentified sp. 3980_1552	Ascomycota	KP889365	300	295/309	95	0.002	0.005	0.003	-	-	-	0.002
Unidentified sp. 3980_1554	Basidiomycota	KM624606	260	240/242	99	-	-	-	0.016	-	0.008	0.002
Unidentified sp. 3980_1558	Mucoromycota	KY992878	310	255/315	81	0.004	0.003	0.003	-	-	-	0.002
Polycauliona phlogina	Ascomycota	GU942736	258	258/258	100	-	0.005	0.003	0.004	-	0.002	0.002
Unidentified sp. 3980_1572	Basidiomycota	DQ294954	285	254/291	87	-	-	-	0.008	0.008	0.008	0.002
Unidentified sp. 3980_1576	Basidiomycota	KP891188	324	324/324	100	0.002	0.005	0.003	-	-	-	0.002
Geomyces sp. 3980_1578	Ascomycota	JX270454	240	240/240	100	0.004	0.003	0.003	-	-	-	0.002
Phlebia acerina	Basidiomycota	KJ714015	289	289/289	100	0.002	0.005	0.003	-	-	-	0.002
Unidentified sp. 3980_1606	Basidiomycota	LC373246	246	54/56	96	-	-	-	0.004	0.012	0.008	0.002
Unidentified sp. 3980_1614	Ascomycota	FJ454916	588	313/314	99	-	-	-	0.012	0.004	0.008	0.002
Merulicium fusisporum	Basidiomycota	EU118647	306	303/306	99	-	-	-	0.008	0.008	0.008	0.002
Unidentified sp. 3980_1636	Ascomycota	KM492986	241	241/241	100	-	-	-	0.016	-	0.008	0.002
Trichophaea sp. 3980_1637	Ascomycota	KC702651	259	259/259	100	-	-	-	0.016	-	0.008	0.002
Unidentified sp. 3980_1704	Ascomycota	HM030586	267	267/267	100	0.007	-	0.003	-	-	-	0.002
Monocillium sp. 3980_1731	Ascomycota	MG827018	263	255/266	96	-	-	-	0.016	-	0.008	0.002
Unidentified sp. 3980_1735	Basidiomycota	KU064147	208	197/210	94	-	-	-	-	0.016	0.008	0.002
Agaricomycetes sp. 3980_1783	Basidiomycota	MH450845	283	282/283	99	0.002	0.005	0.003	-	-	-	0.002
Leohumicola sp.	Ascomycota	AB847019	238	231/241	96	0.007	-	0.003	-	-	-	0.002
Orbilia sp. 3980_1792	Ascomycota	MG372374	280	266/280	95	-	-	-	0.016	-	0.008	0.002
Coniochaeta sp. 3980_1796	Ascomycota	MH866063	250	240/250	96	0.007	-	0.003	-	-	-	0.002
Unidentified sp. 3980_1854	Ascomycota	KM493990	264	255/264	97	0.004	0.003	0.003	-	-	-	0.002
Unidentified sp. 3980_1875	Ascomycota	KP898176	258	245/259	95	-	-	-	0.016	-	0.008	0.002

Periconia sp. 3980_1897	Ascomycota	KY228676	249	249/249	100	0.007	-	0.003	-	-	-	0.002
Leohumicola minima	Ascomycota	HQ691252	241	240/241	99	-	-	-	-	0.016	0.008	0.002
Unidentified sp. 3980_1928	Ascomycota	KX221677	263	262/263	99	0.007	-	0.003	-	-	-	0.002
Unidentified sp. 3980_1945	Ascomycota	EF635815	244	244/244	100	-	-	-	0.008	0.008	0.008	0.002
Hymenoscyphus sp. 3980_1950	Ascomycota	JX977147	241	231/242	95	0.004	-	0.002	0.004	0.004	0.004	0.002
Unidentified sp. 3980_1951	Ascomycota	KP897835	237	237/238	99	-	-	-	0.008	0.008	0.008	0.002
Lophiotrema sp. 3980_1993	Ascomycota	LC194494	261	254/262	97	-	0.002	0.001	0.004	0.008	0.006	0.002
Imleria sp. 3980_2015	Basidiomycota	MF352687	284	284/284	100	0.004	0.003	0.003	-	-	-	0.002
Phialocephala sp. 3980_2094	Ascomycota	MH862480	238	229/239	96	0.004	0.003	0.003	-	-	-	0.002
Dothideomycetes sp. 3980_2118	Ascomycota	KX908469	250	242/250	97	-	-	-	0.016	-	0.008	0.002
Dematiopleospora sp. 3980_2170	Ascomycota	NR_157463	250	241/250	96	-	-	-	0.004	0.012	0.008	0.002
Unidentified sp. 3980_2177	Ascomycota	KP897239	284	284/284	100	0.007	-	0.003	-	-	-	0.002
Phaeosphaeria sp. 3980_2211	Ascomycota	HM136634	244	243/245	99	-	-	-	-	0.016	0.008	0.002
Tomentella sp. 3980_2212	Basidiomycota	KY684674	316	312/316	99	-	-	-	0.004	0.012	0.008	0.002
Unidentified sp. 3980_2216	Basidiomycota	MG827911	304	304/306	99	-	-	-	0.012	0.004	0.008	0.002
Devriesia sp. 3980_2275	Ascomycota	HG937160	239	239/239	100	-	-	-	0.016	-	0.008	0.002
Unidentified sp. 3980_2285	Basidiomycota	KY558359	328	300/329	91	-	-	-	0.012	0.004	0.008	0.002
Pezizales sp. 3980_2356	Ascomycota	AJ534700	296	296/296	100	-	-	-	-	0.012	0.006	0.002
Unidentified sp. 3980_3151	Ascomycota	MG827886	265	263/265	99	-	-	-	0.004	0.012	0.008	0.002
Pleosporales sp. 3980_3365	Ascomycota	MH063649	251	251/251	100	-	-	-	-	0.016	0.008	0.002
Flagellospora sp. 3980_4290	Ascomycota	KC834049	241	234/241	97	-	0.007	0.003	-	-	-	0.002
Talaromyces sp. 3980_4603	Ascomycota	MH857869	248	240/250	96	0.005	0.002	0.003	-	-	-	0.002
Taphrina sp. 3980_4649	Ascomycota	KX147632	295	295/295	100	-	0.002	0.001	0.004	0.008	0.006	0.002
Unidentified sp. 3980_1315	Ascomycota	KY742560	512	265/267	99	0.002	0.003	0.003	-	-	-	0.002
Unidentified sp. 3980_1319	Basidiomycota	KU188658	264	262/263	99	0.005	-	0.003	-	-	-	0.002
Unidentified sp. 3980_1331	Ascomycota	KY006624	239	238/239	99	-	0.005	0.003	-	-	-	0.002
Pseudotomentella mucidula	Basidiomycota	KP753346	313	311/313	99	0.005	-	0.003	-	-	-	0.002
Thelephorales sp. 3980_1344	Basidiomycota	KC840617	341	341/341	100	-	-	-	-	0.012	0.006	0.002
Pholiota limonella	Basidiomycota	MH465085	297	295/297	99	-	0.005	0.003	-	-	-	0.002
Amphinema sp. 3980_1347	Basidiomycota	MF352678	284	282/284	99	-	-	-	0.012	-	0.006	0.002
Sordariomycetes sp. 3980_1349	Ascomycota	MF486041	255	255/255	100	-	-	-	0.012	-	0.006	0.002
Unidentified sp. 3980_1352	Ascomycota	DQ421208	233	228/235	97	0.002	0.003	0.003	-	-	-	0.002
Stagonospora trichophoricola	Ascomycota	MG978344	247	245/247	99	-	0.002	0.001	0.008	-	0.004	0.002
Unidentified sp. 3980_1362	Basidiomycota	KX193345	328	293/334	88	-	-	-	0.012	-	0.006	0.002
Capnodiales sp. 3980_1366	Ascomycota	KJ827239	255	252/255	99	0.004	-	0.002	0.004	-	0.002	0.002
Orbilia aristata	Ascomycota	KT596782	279	278/279	99	-	-	-	-	0.012	0.006	0.002
Unidentified sp. 3980_1371	Cryptomycota	KU063273	270	249/271	92	0.004	-	0.002	0.004	-	0.002	0.002
Lepiota cristata	Basidiomycota	EU081956	304	301/305	99	-	-	-	0.012	-	0.006	0.002
Unidentified sp. 3980_1374	Ascomycota	KP897728	240	238/240	99	0.005	-	0.003	-	-	-	0.002
Unidentified sp. 3980_1378	Ascomycota	MF569888	240	238/240	99	-	-	-	-	0.012	0.006	0.002
Unidentified sp. 3980_1382	Ascomycota	KU062208	243	242/245	99	-	0.002	0.001	-	0.008	0.004	0.002
Aphanobasidium pseudotsugae	Basidiomycota	GU187509	320	316/321	98	0.002	0.003	0.003	-	-	-	0.002
Thelephoraceae sp. 3980_1389	Basidiomycota	MF352786	315	315/315	100	-	-	-	0.012	-	0.006	0.002
Pyrenophora dematioidea	Ascomycota	MH864751	256	256/256	100	0.005	-	0.003	-	-	-	0.002
Rasamsonia sp.	Ascomycota	JF417489	258	257/265	97	-	-	-	0.012	-	0.006	0.002
Unidentified sp. 3980_1398	Basidiomycota	KY810473	638	340/341	99	0.004	0.002	0.003	-	-	-	0.002

Coniochaeta sp. 3980_1404	Ascomycota	KX100366	244	234/244	96	0.002	-	0.001	0.008	-	0.004	0.002
Eurotiales sp. 3980_1405	Ascomycota	MF942901	262	256/262	98	-	-	-	0.004	0.008	0.006	0.002
Postia tephroleuca	Basidiomycota	JX109850	294	294/294	100	-	-	-	-	0.012	0.006	0.002
Lophodermium seditiosum	Ascomycota	KY742560	556	264/266	99	-	-	-	0.012	-	0.006	0.002
Unidentified sp. 3980_1423	Basidiomycota	KU559735	358	63/63	100	-	-	-	0.012	-	0.006	0.002
Unidentified sp. 3980_1425	Ascomycota	JQ759992	237	218/239	91	-	-	-	-	0.012	0.006	0.002
Geoglossales sp. 3980_1427	Ascomycota	MH451301	257	254/258	98	0.004	0.002	0.003	-	-	-	0.002
Unidentified sp. 3980_1429	Ascomycota	KJ827805	260	238/262	91	-	0.005	0.003	-	-	-	0.002
Apiotrichum sp. 3980_1433	Basidiomycota	MK268132	300	291/300	97	0.004	-	0.002	0.004	-	0.002	0.002
Sampaiozyma ingeniosa	Basidiomycota	JQ936165	309	304/310	98	0.004	0.002	0.003	-	-	-	0.002
Melampsoridium betulinum	Basidiomycota	KF031563	313	312/313	99	0.004	0.002	0.003	-	-	-	0.002
Cortinarius caninus	Basidiomycota	EU313201	295	293/295	99	-	-	-	-	0.012	0.006	0.002
Penicillium sp. 3980_1450	Ascomycota	LN901129	553	271/271	100	-	0.005	0.003	-	-	-	0.002
Sistotremastrum niveocremeum	Basidiomycota	MH857381	292	291/292	99	-	-	-	-	0.012	0.006	0.002
Trichaptum biforme	Basidiomycota	JQ901968	299	298/299	99	0.004	-	0.002	0.004	-	0.002	0.002
Filobasidium magnum	Basidiomycota	MH042812	337	336/337	99	-	-	-	0.012	-	0.006	0.002
Unidentified sp. 3980_1461	Ascomycota	KP897606	250	248/249	99	0.004	-	0.002	0.004	-	0.002	0.002
Apiotrichum sp. 3980_1467	Basidiomycota	MK268132	299	291/300	97	0.002	0.003	0.003	-	-	-	0.002
Gomphidius glutinosus	Basidiomycota	LN714548	320	318/320	99	0.002	0.003	0.003	-	-	-	0.002
Coleosporium campanulae	Basidiomycota	KY810468	631	340/341	99	0.002	0.003	0.003	-	-	-	0.002
Unidentified sp. 3980_1479	Basidiomycota	KU685753	328	180/219	82	0.004	0.002	0.003	-	-	-	0.002
Drechmeria balanoides	Ascomycota	EF546660	252	247/253	98	-	-	-	0.012	-	0.006	0.002
Unidentified sp. 3980_1481	Ascomycota	KX194707	246	242/246	98	0.005	-	0.003	-	-	-	0.002
Unidentified sp. 3980_1483	Ascomycota	MG760854	274	270/274	99	0.004	0.002	0.003	-	-	-	0.002
Leotiomycetes sp. 3980_1486	Ascomycota	FJ553339	301	293/302	97	-	0.005	0.003	-	-	-	0.002
Unidentified sp. 3980_1489	Basidiomycota	FR682209	328	320/328	98	0.005	-	0.003	-	-	-	0.002
Unidentified sp. 3980_1491	Ascomycota	KU062040	521	262/266	98	0.002	0.003	0.003	-	-	-	0.002
Unidentified sp. 3980_1493	Ascomycota	KM494173	288	265/290	91	-	-	-	0.008	0.004	0.006	0.002
Unidentified sp. 3980_1494	Basidiomycota	KX147786	317	302/317	95	-	-	-	0.012	-	0.006	0.002
Unidentified sp. 3980_1497	Oomycetes	MF487738	297	107/123	87	-	-	-	-	0.008	0.004	0.001
Pseudoomphalina kalchbrenneri	Basidiomycota	HM191753	294	293/294	99	0.004	0.002	0.003	-	-	-	0.002
Mycoacia fuscoatra	Basidiomycota	KP135367	289	287/289	99	0.004	0.002	0.003	-	-	-	0.002
Pezizomycetes sp. 3980_1502	Ascomycota	JQ759341	261	258/261	99	-	-	-	0.012	-	0.006	0.002
Unidentified sp. 3980_1503	Ascomycota	KR267027	241	241/241	100	0.002	0.003	0.003	-	-	-	0.002
Unidentified sp. 3980_1505	Basidiomycota	KP897328	271	239/242	99	0.002	0.003	0.003	-	-	-	0.002
Stropharia hornemannii	Basidiomycota	AF195596	305	304/305	99	0.004	0.002	0.003	-	-	-	0.002
Cantharellales sp. 3980_1508	Basidiomycota	MF483240	317	313/323	97	0.004	-	0.002	-	0.004	0.002	0.002
Mortierellomycetes sp. 3980_1511	Mucoromycota	MH451940	329	329/330	99	-	0.005	0.003	-	-	-	0.002
Unidentified sp. 3980_1517	Ascomycota	HQ433052	261	250/267	94	0.002	0.003	0.003	-	-	-	0.002
Helotiales sp. 3980_1522	Ascomycota	KJ826654	242	237/242	98	-	0.005	0.003	-	-	-	0.002
Russulales sp. 3980_1523	Basidiomycota	MF482424	342	336/345	97	0.004	0.002	0.003	-	-	-	0.002
Piptocephalis cylindrospora	Zoopagomycota	MG764685	435	432/435	99	-	-	-	0.012	-	0.006	0.002
Unidentified sp. 3980_1533	Mucoromycota	KM494401	345	342/345	99	-	-	-	0.012	-	0.006	0.002
Galerina mycenopsis	Basidiomycota	MH856621	309	307/309	99	-	-	-	-	0.012	0.006	0.002
Unidentified sp. 3980_1537	Basidiomycota	MF511074	303	302/303	99	-	-	-	-	0.012	0.006	0.002
Cortinarius sp. 3980_1544	Basidiomycota	KM576355	301	298/303	98	-	-	-	-	0.012	0.006	0.002

Crocicreas sp. 3980_1546	Ascomycota	JX507682	239	238/239	99	0.005	-	0.003	-	-	-	0.002
Unidentified sp. 3980_1547	Ascomycota	JX136411	245	241/246	98	0.005	-	0.003	-	-	-	0.002
Spizellomycetales sp. 3980_1553	Chytridiomycota	MF484453	300	289/303	95	-	0.005	0.003	-	-	-	0.002
Unidentified sp. 3980_1555	Ascomycota	KX909227	565	276/276	100	-	-	-	0.012	-	0.006	0.002
Archaeorhizomycetes sp.	Ascomycota	MH451735	217	211/217	97	-	-	-	0.012	-	0.006	0.002
Unidentified sp. 3980_1564	Ascomycota	KX909227	573	276/276	100	0.005	-	0.003	-	-	-	0.002
Unidentified sp. 3980_1568	Ascomycota	KX196028	267	257/267	96	-	-	-	0.012	-	0.006	0.002
Unidentified sp. 3980_1571	Basidiomycota	JX507710	320	293/321	91	-	-	-	0.012	-	0.006	0.002
Unidentified sp. 3980_1575	Mucoromycota	MF615405	493	38/40	95	0.004	-	0.002	-	0.004	0.002	0.002
Archaeospora sp. 3980_1583	Mucoromycota	MG429149	281	275/283	97	0.002	0.002	0.002	-	0.004	0.002	0.002
Phaeolepiota aurea	Basidiomycota	MH864957	302	301/302	99	0.002	0.003	0.003	-	-	-	0.002
Unidentified sp. 3980_1589	Ascomycota	KP891409	256	251/256	98	-	0.005	0.003	-	-	-	0.002
Unidentified sp. 3980_1592	Ascomycota	MG207310	243	241/243	99	0.005	-	0.003	-	-	-	0.002
Unidentified sp. 3980_1599	Basidiomycota	HM487021	298	293/298	98	0.005	-	0.003	-	-	-	0.002
Mortierella horticola	Mucoromycota	JX976021	344	340/342	99	-	0.005	0.003	-	-	-	0.002
Pluteus elaphinus	Basidiomycota	NR_153236	279	278/279	99	-	-	-	-	0.012	0.006	0.002
Typhula sp. 3980_1617	Basidiomycota	AB889550	315	305/317	96	0.005	-	0.003	-	-	-	0.002
Unidentified sp. 3980_1627	Ascomycota	EF521253	233	226/233	97	0.004	0.002	0.003	-	-	-	0.002
Unidentified sp. 3980_1628	Chytridiomycota	MF481998	324	269/355	76	-	-	-	-	0.012	0.006	0.002
Unidentified sp. 3980_1629	Ascomycota	KP892161	256	255/256	99	0.002	0.003	0.003	-	-	-	0.002
Tremellales sp. 3980_1632	Basidiomycota	MF483814	311	310/311	99	0.004	0.002	0.003	-	-	-	0.002
Unidentified sp. 3980_1635	Basidiomycota	KX816144	302	290/304	95	0.005	-	0.003	-	-	-	0.002
Unidentified sp. 3980_1639	Ascomycota	KT195106	217	215/217	99	-	-	-	0.012	-	0.006	0.002
Unidentified sp. 3980_1647	Ascomycota	MG827779	245	245/245	100	-	-	-	0.008	0.004	0.006	0.002
Unidentified sp. 3980_1661	Basidiomycota	KU687386	627	302/307	98	0.004	-	0.002	-	0.004	0.002	0.002
Unidentified sp. 3980_1677	Ascomycota	KP891301	285	248/293	85	0.005	-	0.003	-	-	-	0.002
Clitocybe ditopa	Basidiomycota	JF907805	302	301/302	99	-	0.005	0.003	-	-	-	0.002
Cantharellales sp. 3980_1707	Basidiomycota	KY228714	320	303/309	98	0.005	-	0.003	-	-	-	0.002
Unidentified sp. 3980_1728	Ascomycota	MG828152	239	238/239	99	-	0.005	0.003	-	-	-	0.002
Unidentified sp. 3980_1736	Ascomycota	KP897589	243	242/244	99	-	-	-	-	0.012	0.006	0.002
Amphinema sp. 3980_1762	Basidiomycota	KP753294	288	288/288	100	-	0.005	0.003	-	-	-	0.002
Pezicula ericae	Ascomycota	NR_155653	240	240/240	100	-	0.002	0.001	0.004	0.004	0.004	0.002
Unidentified sp. 3980_1782	Basidiomycota	MG207477	293	243/301	81	0.004	0.002	0.003	-	-	-	0.002
Tubaria furfuracea	Basidiomycota	KY706157	299	298/299	99	0.004	0.002	0.003	-	-	-	0.002
Sordariomycetes sp. 3980_1801	Ascomycota	KY742564	248	246/248	99	-	-	-	0.012	-	0.006	0.002
Inocybe flocculosa	Basidiomycota	JF908124	292	292/292	100	-	0.005	0.003	-	-	-	0.002
Coleosporium euphrasiae	Basidiomycota	KY810469	673	341/341	100	-	-	-	-	0.012	0.006	0.002
Unidentified sp. 3980_1829	Basidiomycota	KU064115	345	327/329	99	-	-	-	-	0.012	0.006	0.002
Unidentified sp. 3980_1862	Ascomycota	MF976273	255	254/255	99	-	-	-	0.008	0.004	0.006	0.002
Unidentified sp. 3980_1867	Ascomycota	FJ438376	241	221/244	91	-	0.005	0.003	-	-	-	0.002
Unidentified sp. 3980_1876	Ascomycota	KU687401	243	217/247	88	0.004	0.002	0.003	-	-	-	0.002
Coniochaetales sp. 3980_1886	Ascomycota	KJ827291	242	236/242	98	0.004	0.002	0.003	-	-	-	0.002
Unidentified sp. 3980_1887	Basidiomycota	MG828266	268	254/269	94	-	0.005	0.003	-	-	-	0.002
Setomelanomma holmii	Ascomycota	MH863660	250	249/250	99	-	-	-	0.008	0.004	0.006	0.002
Unidentified sp. 3980_1927	Ascomycota	JN890389	266	246/268	92	-	-	-	0.004	0.008	0.006	0.002
Unidentified sp. 3980_1931	Ascomycota	JF449819	249	231/252	92	0.004	0.002	0.003	-	-	-	0.002

Inocybe stenospora	Basidiomycota	MH366598	298	233/233	100	-	-	-	-	0.012	0.006	0.002
Schizopora paradoxa	Basidiomycota	MH857218	283	283/283	100	0.002	-	0.001	-	0.008	0.004	0.002
Unidentified sp. 3980_1944	Ascomycota	KU063896	241	238/242	98	0.004	0.002	0.003	-	-	-	0.002
Cystodermella myriadocystis	Basidiomycota	LN878145	306	306/306	100	0.002	0.003	0.003	-	-	-	0.002
Lophodermium pinastri	Ascomycota	KY742598	238	237/238	99	0.002	0.003	0.003	-	-	-	0.002
Unidentified sp. 3980_1984	Ascomycota	HQ433050	254	228/262	87	0.004	0.002	0.003	-	-	-	0.002
Unidentified sp. 3980_1991	Ascomycota	KR267195	238	231/239	97	-	-	-	0.012	-	0.006	0.002
Chalciporus piperatus	Basidiomycota	AF335457	310	310/310	100	-	-	-	0.012	-	0.006	0.002
Unidentified sp. 3980_2007	Ascomycota	MG827877	255	254/255	99	-	-	-	0.008	0.004	0.006	0.002
Unidentified sp. 3980_2009	Ascomycota	EF635749	281	281/281	100	-	-	-	0.012	-	0.006	0.002
Helotiales sp. 3980_2044	Ascomycota	KJ827376	243	242/243	99	-	0.002	0.001	0.008	-	0.004	0.002
Umbelopsis ramanniana	Mucoromycota	KU516642	315	315/315	100	-	-	-	-	0.012	0.006	0.002
Hyalorbilia sp. 3980_2058	Ascomycota	KT222380	339	96/102	94	0.004	0.002	0.003	-	-	-	0.002
Leotiomycetes sp. 3980_2061	Ascomycota	KR266300	238	237/238	99	0.002	0.003	0.003	-	-	-	0.002
Hebeloma helodes	Basidiomycota	KM390772	307	306/307	99	0.005	-	0.003	-	-	-	0.002
Unidentified sp. 3980_2073	Ascomycota	KY947864	250	226/252	90	-	-	-	0.004	0.008	0.006	0.002
Unidentified sp. 3980_2077	Cryptomycota	KX220281	353	300/355	85	-	0.002	0.001	-	0.008	0.004	0.002
Unidentified sp. 3980_2078	Ascomycota	MG206717	246	244/246	99	-	0.002	0.001	0.004	-	0.002	0.002
Unidentified sp. 3980_2079	Ascomycota	MH858317	266	244/265	92	-	0.005	0.003	-	-	-	0.002
Unidentified sp. 3980_2089	Ascomycota	KP897182	256	256/256	100	-	-	-	0.012	-	0.006	0.002
Flammula alnicola	Basidiomycota	KR153192	307	307/307	100	-	0.005	0.003	-	-	-	0.002
Unidentified sp. 3980_2104	Basidiomycota	MG827579	252	250/252	99	-	0.005	0.003	-	-	-	0.002
Antrodia pulvinascens	Basidiomycota	EU340899	281	281/281	100	-	-	-	-	0.012	0.006	0.002
Unidentified sp. 3980_2120	Ascomycota	KT196299	263	237/251	94	0.004	-	0.002	0.004	-	0.002	0.002
Unidentified sp. 3980_2126	Basidiomycota	FR682177	325	325/325	100	-	-	-	0.012	-	0.006	0.002
Gymnopus sp. 3980_2138	Basidiomycota	KY026760	366	366/366	100	0.004	0.002	0.003	-	-	-	0.002
Leptobacillium leptobactrum	Ascomycota	NR_154111	282	280/282	99	0.004	-	0.002	-	-	-	0.002
Unidentified sp. 3980_2147	Ascomycota	KF675719	256	213/227	94	-	-	-	0.012	-	0.006	0.002
Cortinarius anomalus	Basidiomycota	KY595995	294	294/294	100	0.005	-	0.003	-	-	-	0.002
Unidentified sp. 3980_2174	Ascomycota	MG827785	246	239/250	96	-	0.005	0.003	-	-	-	0.002
Gibellulopsis nigrescens	Ascomycota	MH856763	265	265/265	100	-	0.005	0.003	-	-	-	0.002
Unidentified sp. 3980_2190	Ascomycota	KU062011	243	237/244	97	0.005	-	0.003	-	-	-	0.002
Leptosphaeria etheridgei	Ascomycota	MH863859	249	249/249	100	0.005	-	0.003	-	-	-	0.002
Aspergillus sydowii	Ascomycota	MK267403	260	260/260	100	-	-	-	-	0.012	0.006	0.002
Peniophorella pubera	Basidiomycota	MH857358	302	302/302	100	0.005	-	0.003	-	-	-	0.002
Unidentified sp. 3980_2217	Ascomycota	JX976998	236	219/242	90	0.004	-	0.002	0.004	-	0.002	0.002
Unidentified sp. 3980_2222	Ascomycota	KP897373	249	249/249	100	0.002	0.003	0.003	-	-	-	0.002
Crocicreas coronatum	Ascomycota	MH858141	239	239/239	100	0.004	0.002	0.003	-	-	-	0.002
Resinicium bicolor	Basidiomycota	MF511087	291	291/291	100	0.005	-	0.003	-	-	-	0.002
Unidentified sp. 3980_2236	Ascomycota	KT219458	254	253/255	99	-	-	-	0.012	-	0.006	0.002
Pezizomycetes sp. 3980_2238	Ascomycota	MH450851	251	250/251	99	-	0.002	0.001	-	0.008	0.004	0.002
Xerocomus ferrugineus	Ascomycota	HQ207698	361	361/361	100	0.005	-	0.003	-	-	-	0.002
Unidentified sp. 3980_2251	Basidiomycota	KX403704	251	200/200	100	-	-	-	0.012	-	0.006	0.002
Unidentified sp. 3980_2258	Ascomycota	KP892306	246	239/247	97	-	0.005	0.003	-	-	-	0.002
Cortinarius scandens	Basidiomycota	GQ159849	282	282/282	100	-	-	-	0.012	-	0.006	0.002
Corticium confine	Basidiomycota	KP814404	299	299/299	100	-	-	-	0.008	0.004	0.006	0.002

Unidentified sp. 3980_2279	Ascomycota	KU687401	244	222/244	91	-	-	-	-	0.012	0.006	0.002
Unidentified sp. 3980_2292	Ascomycota	KP891402	205	200/205	98	-	-	-	-	0.012	0.006	0.002
Unidentified sp. 3980_2293	Ascomycota	LT608101	242	242/242	100	-	-	-	-	0.012	0.006	0.002
Sarcodon squamosus	Basidiomycota	MG597419	368	368/368	100	0.002	0.003	0.003	-	-	-	0.002
Agaricomycetes sp. 3980_2324	Basidiomycota	FJ554449	296	286/297	96	-	-	-	0.004	0.008	0.006	0.002
Unidentified sp. 3980_2331	Basidiomycota	KX194805	298	298/298	100	-	-	-	-	0.012	0.006	0.002
Unidentified sp. 3980_2341	Basidiomycota	KU948755	293	270/292	92	0.004	0.002	0.003	-	-	-	0.002
Unidentified sp. 3980_2755	Ascomycota	KP889799	240	229/242	95	0.005	-	0.003	-	-	-	0.002
Unidentified sp. 3980_2831	Basidiomycota	KU064132	283	278/290	96	0.002	-	0.001	-	0.008	0.004	0.002
Trichoderma semiorbis	Ascomycota	EF596948	260	260/260	100	-	-	-	0.008	0.004	0.006	0.002
Chaetothyriales sp. 3980_3112	Ascomycota	KJ826662	254	247/255	97	-	0.005	0.003	-	-	-	0.002
Malasseziales sp. 3980_3115	Basidiomycota	MF486499	248	248/248	100	0.002	0.003	0.003	-	-	-	0.002
Unidentified sp. 3980_3229	Ascomycota	MG206459	252	243/257	95	-	-	-	0.012	-	0.006	0.002
Unidentified sp. 3980_3462	Entorrhizomycetes	KX223055	239	236/243	97	0.004	-	0.002	0.004	-	0.002	0.002
Unidentified sp. 3980_3503	Ascomycota	KP888002	266	262/266	98	0.004	0.002	0.003	-	-	-	0.002
Unidentified sp. 3980_3792	Ascomycota	KP897667	248	248/248	100	-	-	-	-	0.012	0.006	0.002
Unidentified sp. 3980_4172	Ascomycota	KP891719	216	215/216	99	0.005	-	0.003	-	-	-	0.002
Sordariomycetes sp. 3980_4286	Ascomycota	MH451782	262	261/262	99	0.005	-	0.003	-	-	-	0.002
Unidentified sp. 3980_4425	Ascomycota	MG206433	237	228/237	96	0.005	-	0.003	-	-	-	0.002
Neoascochyta cylindrispora	Ascomycota	NR_158282	249	249/249	100	-	-	-	0.008	0.004	0.006	0.002
Datronia mollis	Basidiomycota	KJ668554	286	286/286	100	-	-	-	-	0.012	0.006	0.002
Unidentified sp. 3980_4654	Ascomycota	MH451700	269	158/194	81	-	-	-	0.012	-	0.006	0.002
Unidentified sp. 3980_4786	Basidiomycota	KX195744	309	292/310	94	0.002	0.003	0.003	-	-	-	0.002
Violella fucata	Ascomycota	KX132968	263	263/263	100	0.004	0.004	0.004	-	-	-	0.002
Unidentified sp. 3980_5134	Basidiomycota	MG827945	245	220/249	88	-	-	-	-	0.012	0.006	0.002
Phaeohelotium sp. 3980_5155	Ascomycota	KT876976	239	232/239	97	-	-	-	0.012	-	0.006	0.002
Cyclaneusma minus	Ascomycota	MH860757	245	245/245	100	0.005	-	0.003	-	-	-	0.002
Unidentified sp. 3980_5217	Ascomycota	MG828304	243	217/245	89	-	-	-	-	0.012	0.006	0.002
Venturiaceae sp. 3980_5310	Ascomycota	KY996555	244	244/244	100	0.004	0.002	0.003	-	-	-	0.002
Mucor luteus	Mucoromycota	EU484251	272	272/272	100	-	0.005	0.003	-	-	-	0.002
Unidentified sp. 3980_5461	Ascomycota	EU490097	260	229/266	86	0.002	0.002	0.002	-	-	-	0.002
Bulleribasidium oberjochense	Basidiomycota	KY101803	225	225/225	100	-	-	-	0.004	0.008	0.006	0.002
Psathyrella sublatispora	Basidiomycota	KC992854	297	295/297	99	-	0.003	0.002	-	-	-	0.001
Polyporales sp. 3980_1643	Basidiomycota	FJ475670	280	280/280	100	0.004	-	0.002	-	-	-	0.001
Agaricomycetes sp. 3980_1645	Basidiomycota	MH451807	335	333/335	99	-	-	-	0.004	0.004	0.004	0.001
Venturiales sp. 3980_1646	Ascomycota	KU208047	250	244/251	97	-	-	-	0.008	-	0.004	0.001
Vishniacozyma foliicola	Basidiomycota	KY105821	235	234/235	99	-	0.003	0.002	-	-	-	0.001
Cortinarius anisatus	Basidiomycota	DQ117930	279	278/279	99	-	0.003	0.002	-	-	-	0.001
Helotiales sp. 3980_1654	Ascomycota	FR846484	242	242/242	100	0.002	-	0.001	0.004	-	0.002	0.001
Postia placenta	Basidiomycota	KC543146	319	318/319	99	0.002	0.002	0.002	-	-	-	0.001
Naematelia encephala	Basidiomycota	FN563135	254	251/254	99	0.002	0.002	0.002	-	-	-	0.001
Agaricales sp. 3980_1667	Basidiomycota	MF484691	294	287/294	98	0.002	-	0.001	-	0.004	0.002	0.001
Unidentified sp. 3980_1670	Basidiomycota	KY810473	643	342/343	99	0.002	0.002	0.002	-	-	-	0.001
Unidentified sp. 3980_1673	Ascomycota	KP897985	238	237/238	99	-	-	-	-	0.008	0.004	0.001
Unidentified sp. 3980_1675	Basidiomycota	KP892295	277	277/277	100	-	0.002	0.001	-	-	-	0.001
Polyporus varius	Basidiomycota	KX533920	292	289/293	99	0.002	0.002	0.002	-	-	-	0.001

Unidentified sp. 3980_1679	Zoopagomycota	MF485027	285	227/301	75	-	-	-	-	0.008	0.004	0.001
Galzinia sp. 3980_1682	Basidiomycota	JN649343	297	286/298	96	-	-	-	0.004	0.004	0.004	0.001
Clavulinaceae sp. 3980_1684	Basidiomycota	JX507640	330	329/330	99	-	-	-	0.004	0.004	0.004	0.001
Unidentified sp. 3980_1685	Ascomycota	KP991015	562	270/270	100	0.004	-	0.002	-	-	-	0.001
Unidentified sp. 3980_1687	Mucoromycota	HQ022205	594	316/319	99	0.004	-	0.002	-	-	-	0.001
Unidentified sp. 3980_1688	Ascomycota	KT323164	554	272/275	99	0.004	-	0.002	-	-	-	0.001
Unidentified sp. 3980_1693	Ascomycota	KF673749	253	243/253	96	0.004	-	0.002	-	-	-	0.001
Unidentified sp. 3980_1696	Ascomycota	JX998700	272	50/53	94	-	0.003	0.002	-	-	-	0.001
Helotiales sp. 3980_1697	Ascomycota	KJ826684	239	231/241	96	-	-	-	0.008	-	0.004	0.001
Unidentified sp. 3980_1698	Basidiomycota	AF481376	289	286/289	96	-	-	-	-	0.008	0.004	0.001
Unidentified sp. 3980_1702	Ascomycota	MG888613	566	274/275	99	0.002	0.002	0.002	-	-	-	0.001
Unidentified sp. 3980_1703	Ascomycota	MH450351	248	248/248	100	-	-	-	0.008	-	0.004	0.001
Unidentified sp. 3980_1705	Basidiomycota	AF042453	255	214/259	83	0.004	-	0.002	-	-	-	0.001
Cortinarius lobatus	Basidiomycota	MH784810	279	275/279	99	0.004	-	0.002	-	-	-	0.001
Trechispora incisa	Basidiomycota	AF347085	294	294/295	99	-	-	-	0.008	-	0.004	0.001
Drepanopeziza sp. 3980_1712	Ascomycota	MH855774	240	233/240	97	0.002	0.002	0.002	-	-	-	0.001
Unidentified sp. 3980_1713	Ascomycota	KT243303	248	247/253	98	0.002	-	0.001	-	0.004	0.002	0.001
Unidentified sp. 3980_1715	Ascomycota	KX194987	265	252/267	94	-	-	-	0.004	0.004	0.004	0.001
Unidentified sp. 3980_1716	Ascomycota	KY687765	285	273/285	96	-	-	-	-	0.008	0.004	0.001
Vanrija longa	Basidiomycota	NR_155992	257	257/257	100	-	0.002	0.001	-	0.004	0.002	0.001
Cortinarius alboviolaceus	Basidiomycota	KJ705149	279	277/279	99	-	0.003	0.002	-	-	-	0.001
Unidentified sp. 3980_1720	Basidiomycota	KT241667	233	217/241	90	-	-	-	0.004	0.004	0.004	0.001
Prosthemium betulinum	Ascomycota	MH864356	245	244/245	99	0.004	-	0.002	-	-	-	0.001
Russula paludosa	Basidiomycota	LC192779	358	358/358	100	-	-	-	-	0.008	0.004	0.001
Unidentified sp. 3980_1724	Ascomycota	KX908472	553	256/258	99	-	0.008	0.004	-	-	-	0.001
Rhizophydiales sp. 3980_1725	Chytridiomycota	MF487072	236	230/236	97	0.004	-	0.002	-	-	-	0.001
Unidentified sp. 3980_1726	Basidiomycota	KP897644	263	256/264	97	-	-	-	0.008	-	0.004	0.001
Unidentified sp. 3980_1729	Ascomycota	KU062306	237	229/240	95	-	-	-	-	0.008	0.004	0.001
Tricholoma terreum	Basidiomycota	LT000116	306	306/306	100	0.002	0.002	0.002	-	-	-	0.001
Corticiales sp. 3980_1733	Basidiomycota	FJ475677	313	311/313	99	0.002	-	0.001	-	0.004	0.002	0.001
Mortierellales sp. 3980_1740	Mucoromycota	MF482523	336	336/337	99	-	-	-	-	0.008	0.004	0.001
Unidentified sp. 3980_1742	Basidiomycota	KY810473	623	339/341	99	0.004	-	0.002	-	-	-	0.001
Unidentified sp. 3980_1743	Ascomycota	KY742560	542	264/266	99	-	-	-	0.008	-	0.004	0.001
Unidentified sp. 3980_1744	Basidiomycota	KX194736	332	259/340	76	-	-	-	0.008	-	0.004	0.001
Unidentified sp. 3980_1745	Ascomycota	KT219399	244	240/244	98	0.002	0.002	0.002	-	-	-	0.001
Sydowia sp. 3980_1746	Ascomycota	KY246323	255	244/253	96	0.002	0.002	0.002	-	-	-	0.001
Hymenochaete sp. 3980_1748	Basidiomycota	JN230419	298	290/300	97	-	0.004	0.002	-	-	-	0.001
Unidentified sp. 3980_1750	Ascomycota	KX815480	562	274/278	90	-	-	-	-	0.008	0.004	0.001
Spizellomycetales sp. 3980_1754	Chytridiomycota	MF483268	322	320/323	99	-	0.003	0.002	-	-	-	0.001
Unidentified sp. 3980_1755	Ascomycota	AM260816	314	48/55	87	-	-	-	0.008	-	0.004	0.001
Unidentified sp. 3980_1756	Basidiomycota	KP897325	249	248/249	99	-	0.003	0.002	-	-	-	0.001
Unidentified sp. 3980_1757	Ascomycota	KP897304	557	265/266	99	0.004	-	0.002	-	-	-	0.001
Unidentified sp. 3980_1760	Ascomycota	KX194987	266	252/270	93	0.002	0.002	0.002	-	-	-	0.001
Unidentified sp. 3980_1765	Basidiomycota	KU687386	613	302/307	98	-	-	-	-	0.008	0.004	0.001
Unidentified sp. 3980_1768	Ascomycota	FN435802	287	241/257	94	0.002	0.002	0.002	-	-	-	0.001
Galerina vittiformis	Basidiomycota	MK583317	311	311/311	100	-	-	-	0.008	-	0.004	0.001

Unidentified sp. 3980_1770	Basidiomycota	KP898116	303	258/324	80	0.004	-	0.002	-	-	-	0.001
Ceratobasidium sp. 3980_1772	Basidiomycota	KC590526	320	313/321	98	-	-	-	0.004	0.004	0.004	0.001
Unidentified sp. 3980_1773	Basidiomycota	KX194839	297	297/297	100	0.002	0.002	0.002	-	-	-	0.001
Unidentified sp. 3980_1775	Ascomycota	KU062862	239	235/241	98	-	0.008	0.004	-	-	-	0.001
Cortinarius sp. 3980_1776	Basidiomycota	MH784791	276	276/276	100	-	0.003	0.002	-	-	-	0.001
Unidentified sp. 3980_1779	Basidiomycota	KX220253	316	298/319	93	0.002	0.002	0.002	-	-	-	0.001
Thelephorales sp. 3980_1784	Basidiomycota	MF484047	384	379/385	98	-	-	-	0.004	0.004	0.004	0.001
Helotiales sp. 3980_1786	Ascomycota	HG937142	237	237/238	99	-	-	-	-	0.008	0.004	0.001
Unidentified sp. 3980_1787	Ascomycota	KU062040	540	262/266	98	0.004	-	0.002	-	-	-	0.001
Unidentified sp. 3980_1790	Ascomycota	KU188782	558	273/274	99	-	0.003	0.002	-	-	-	0.001
Russulales sp. 3980_1791	Basidiomycota	MF486819	242	239/242	99	-	0.003	0.002	-	-	-	0.001
Unidentified sp. 3980_1794	Basidiomycota	KU062017	310	304/311	98	-	-	-	-	0.008	0.004	0.001
Unidentified sp. 3980_1800	Ascomycota	MF485520	312	294/316	93	0.004	-	0.002	-	-	-	0.001
Unidentified sp. 3980_1804	Mucoromycota	MF942933	607	311/313	99	-	-	-	-	0.008	0.004	0.001
Unidentified sp. 3980_1805	Ascomycota	KU559709	256	48/50	96	0.002	0.002	0.002	-	-	-	0.001
Agaricales sp. 3980_1806	Basidiomycota	KX402030	236	193/200	97	-	-	-	-	0.008	0.004	0.001
Mycena zephirus	Basidiomycota	MH856339	304	304/304	100	-	-	-	0.004	0.004	0.004	0.001
Unidentified sp. 3980_1809	Ascomycota	AJ972820	249	235/249	94	0.004	-	0.002	-	-	-	0.001
Spizellomycetales sp. 3980_1812	Chytridiomycota	MF482782	330	326/331	98	-	-	-	-	0.008	0.004	0.001
Unidentified sp. 3980_1813	Ascomycota	KT965054	242	235/243	97	-	0.002	0.001	-	-	-	0.001
Unidentified sp. 3980_1815	Mucoromycota	KT736107	274	244/263	93	-	-	-	0.008	-	0.004	0.001
Unidentified sp. 3980_1816	Basidiomycota	MG207477	311	238/317	75	-	-	-	0.008	-	0.004	0.001
Unidentified sp. 3980_1820	Ascomycota	KX194188	236	233/236	99	-	-	-	0.008	-	0.004	0.001
Rhizophydiales sp. 3980_1821	Chytridiomycota	MF482921	328	327/329	99	-	-	-	-	0.008	0.004	0.001
Lycoperdaceae sp. 3980_1822	Basidiomycota	EF635725	305	304/305	99	-	0.003	0.002	-	-	-	0.001
Scoliciosporum chlorococcum	Ascomycota	FR799323	243	226/229	99	-	-	-	0.008	-	0.004	0.001
Glomerales sp. 3980_1825	Mucoromycota	MF483863	310	309/310	99	-	-	-	0.004	0.004	0.004	0.001
Exidia glandulosa	Basidiomycota	MF161201	284	282/284	99	-	-	-	0.004	0.004	0.004	0.001
Pseudeurotiaceae sp. 3980_1828	Ascomycota	KJ826807	241	238/241	99	0.004	-	0.002	-	-	-	0.001
Unidentified sp. 3980_1830	Basidiomycota	AM999659	328	287/293	98	-	-	-	0.008	-	0.004	0.001
Unidentified sp. 3980_1831	Ascomycota	KP897583	273	272/273	99	-	-	-	0.008	-	0.004	0.001
Naganishia adeliensis	Basidiomycota	MK226217	314	313/314	99	0.002	0.002	0.002	-	-	-	0.001
Unidentified sp. 3980_1834	Ascomycota	KX195491	330	319/330	97	-	-	-	0.008	-	0.004	0.001
Unidentified sp. 3980_1837	Basidiomycota	MG828239	296	65/71	92	-	0.003	0.002	-	-	-	0.001
Unidentified sp. 3980_1838	Ascomycota	EU940171	257	63/63	100	-	0.003	0.002	-	-	-	0.001
Pezizales sp. 3980_1839	Ascomycota	KJ828024	242	232/242	96	-	-	-	0.008	-	0.004	0.001
Orbiliales sp. 3980_1840	Ascomycota	MF487145	234	232/234	99	-	-	-	0.004	0.004	0.004	0.001
Unidentified sp. 3980_1841	Basidiomycota	GU328519	318	279/323	86	-	-	-	-	0.008	0.004	0.001
Hypochnicium lundellii	Basidiomycota	AY781277	296	272/274	99	-	-	-	0.008	-	0.004	0.001
Spirosphaera sp. 3980_1846	Ascomycota	AY616233	245	231/240	96	-	-	-	0.008	-	0.004	0.001
Unidentified sp. 3980_1847	Ascomycota	KP897304	551	262/263	99	0.002	0.002	0.002	-	-	-	0.001
Unidentified sp. 3980_1848	Basidiomycota	JN168721	453	46/50	92	-	-	-	-	0.008	0.004	0.001
Rhizophydiales sp. 3980_1850	Chytridiomycota	MF482864	328	324/330	98	-	-	-	-	0.008	0.004	0.001
Unidentified sp. 3980_1851	Basidiomycota	KY104261	321	61/64	95	0.002	0.002	0.002	-	-	-	0.001
Unidentified sp. 3980_1852	Basidiomycota	MF482055	377	304/378	80	-	-	-	0.008	-	0.004	0.001
Mucor abundans	Mucoromycota	MH971277	266	266/267	99	-	0.003	0.002	-	-	-	0.001

Rigidoporus corticola	Basidiomycota	KC176667	324	321/324	99	0.004	-	0.002	-	-	-	0.001
Unidentified sp. 3980_1858	Ascomycota	MG827839	264	263/264	99	0.004	-	0.002	-	-	-	0.001
Elaphomyces granulatus	Ascomycota	MG597454	290	288/290	99	0.002	0.002	0.002	-	-	-	0.001
Unidentified sp. 3980_1860	Ascomycota	KT695389	259	212/269	79	0.002	0.002	0.002	-	-	-	0.001
Scytinostroma hemidichophyticum	Basidiomycota	MH861818	313	313/314	99	0.002	0.002	0.002	-	-	-	0.001
Tephrocybe confusa	Basidiomycota	KP192548	297	294/297	99	0.004	-	0.002	-	-	-	0.001
Diaporthales sp. 3980_1868	Ascomycota	MF486259	248	246/251	98	0.002	0.002	0.002	-	-	-	0.001
Chytridiomycota sp. 3980_1870	Chytridiomycota	MH451356	329	328/329	99	-	0.002	0.001	0.004	-	0.002	0.001
Unidentified sp. 3980_1872	Ascomycota	JN638280	260	212/262	81	-	-	-	-	0.008	0.004	0.001
Unidentified sp. 3980_1874	Ascomycota	MF570414	302	300/302	99	-	0.003	0.002	-	-	-	0.001
Coleosporium senecionis	Basidiomycota	KY810473	617	339/341	99	0.004	-	0.002	-	-	-	0.001
Unidentified sp. 3980_1881	Ascomycota	KJ827857	244	232/245	95	-	0.003	0.002	-	-	-	0.001
Exobasidium sp. 3980_1882	Basidiomycota	FJ896135	294	284/295	96	0.002	0.002	0.002	-	-	-	0.001
Trechisporales sp. 3980_1884	Basidiomycota	MK131684	321	321/321	100	-	0.003	0.002	-	-	-	0.001
Botryobasidium subcoronatum	Basidiomycota	EU118607	315	314/315	99	-	-	-	0.008	-	0.004	0.001
Pezizomycetes sp. 3980_1889	Ascomycota	MF486079	248	242/254	95	0.004	-	0.002	-	-	-	0.001
Unidentified sp. 3980_1890	Ascomycota	FR682343	267	264/268	98	-	-	-	-	0.008	0.004	0.001
Clavaria amoenoides	Basidiomycota	MF972891	301	256/256	100	-	0.003	0.002	-	-	-	0.001
Unidentified sp. 3980_1892	Ascomycota	MF666086	253	198/200	99	-	-	-	0.008	-	0.004	0.001
Unidentified sp. 3980_1894	Basidiomycota	AM902059	387	385/387	99	0.002	0.002	0.002	-	-	-	0.001
Therrya fuckelii	Ascomycota	JF793672	241	239/241	99	-	0.002	0.001	-	-	-	0.001
Unidentified sp. 3980_1899	Mucoromycota	MF976340	254	245/255	96	-	0.002	0.001	-	0.004	0.002	0.001
Acaulium caviariforme	Ascomycota	NR_146259	274	270/276	98	0.004	-	0.002	-	-	-	0.001
Leotiomycetes sp. 3980_1901	Ascomycota	KJ826782	240	238/240	99	-	-	-	-	0.008	0.004	0.001
Chaetomium globosum	Ascomycota	MH861660	247	247/247	100	0.004	-	0.002	-	-	-	0.001
Unidentified sp. 3980_1905	Mucoromycota	MF485219	275	262/282	93	0.002	0.002	0.002	-	-	-	0.001
Helotiales sp. 3980_1906	Ascomycota	AB848489	239	238/239	99	-	0.003	0.002	-	-	-	0.001
Cryptococcus sp. 3980_1907	Basidiomycota	HG324303	320	311/324	96	-	-	-	-	0.004	0.004	0.001
Unidentified sp. 3980_1908	Ascomycota	KF297132	256	236/261	90	-	0.003	0.002	-	-	-	0.001
Coleosporium senecionis	Basidiomycota	KY810473	625	336/341	99	-	-	-	-	0.008	0.004	0.001
Stereum hirsutum	Basidiomycota	EU673087	296	294/296	99	-	-	-	-	0.008	0.004	0.001
Unidentified sp. 3980_1915	Ascomycota	MK211238	566	269/269	100	-	-	-	0.008	-	0.004	0.001
Unidentified sp. 3980_1920	Ascomycota	KX908472	503	255/258	99	-	-	-	-	0.008	0.004	0.001
Unidentified sp. 3980_1929	Mucoromycota	MF481786	362	352/363	97	-	0.002	0.001	0.004	-	0.002	0.001
Unidentified sp. 3980_1930	Ascomycota	KT215241	284	256/308	83	0.004	-	0.002	-	-	-	0.001
Unidentified sp. 3980_1933	Ascomycota	KX908472	516	254/258	98	-	-	-	0.004	0.004	0.004	0.001
Glomeromycotina sp. 3980_1934	Mucoromycota	EF619906	224	220/225	98	-	-	-	-	0.008	0.004	0.001
Capnodiales sp. 3980_1936	Ascomycota	KJ827253	244	243/244	99	-	0.008	0.004	-	-	-	0.001
Mucoromycotina sp. 3980_1939	Mucoromycota	KU208027	297	293/299	98	-	-	-	-	0.008	0.004	0.001
Lophiostoma sp. 3980_1940	Ascomycota	KP843505	257	251/259	97	-	-	-	0.008	-	0.004	0.001
Unidentified sp. 3980_1941	Ascomycota	KU061434	228	188/228	82	0.002	0.002	0.002	-	-	-	0.001
Unidentified sp. 3980_1942	Ascomycota	MF570553	261	258/261	99	-	-	-	-	0.008	0.004	0.001
Unidentified sp. 3980_1943	Basidiomycota	KR266141	298	266/304	88	0.002	0.002	0.002	-	-	-	0.001
Unidentified sp. 3980_1947	Ascomycota	KM494170	329	318/329	97	-	-	-	-	0.004	0.002	0.001
Unidentified sp. 3980_1948	Basidiomycota	KP892390	307	307/307	100	0.002	0.002	0.002	-	-	-	0.001
Pleosporales sp. 3980_1952	Ascomycota	MH451167	247	246/247	99	0.004	-	0.002	-	-	-	0.001

Deconica coprophila	Basidiomycota	HM035073	299	298/299	99	-	0.003	0.002	-	-	-	0.001
Agaricomycetes sp. 3980_1955	Basidiomycota	MH450469	318	317/318	99	-	0.003	0.002	-	-	-	0.001
Unidentified sp. 3980_1957	Ascomycota	KR267215	210	206/210	98	-	-	-	0.004	0.004	0.004	0.001
Pluteus pseudorobertii	Basidiomycota	KJ009769	284	283/284	99	-	-	-	0.008	-	0.004	0.001
Inocybe myriadophylla	Basidiomycota	MF807958	326	324/326	99	-	0.003	0.002	-	-	-	0.001
Unidentified sp. 3980_1960	Ascomycota	MG207422	545	249/251	99	-	-	-	-	0.008	0.004	0.001
Chaetothyriales sp. 3980_1964	Ascomycota	KJ827417	249	246/249	99	-	0.003	0.002	-	-	-	0.001
Cercospora gomphrenigena	Ascomycota	MK442573	238	237/238	99	-	-	-	0.004	0.004	0.004	0.001
Cortinarius suberi	Basidiomycota	MG597360	282	280/281	99	-	0.003	0.002	-	-	-	0.001
Unidentified sp. 3980_1969	Basidiomycota	MK163753	296	121/138	88	0.002	0.002	0.002	-	-	-	0.001
Unidentified sp. 3980_1970	Ascomycota	JX535167	572	274/275	99	0.004	-	0.002	-	-	-	0.001
Unidentified sp. 3980_1975	Basidiomycota	KY810473	636	339/341	99	0.004	0.004	0.002	-	-	-	0.001
Leotiomycetes sp. 3980_1978	Ascomycota	MF485586	269	265/269	99	-	0.003	0.002	-	-	-	0.001
Unidentified sp. 3980_1982	Basidiomycota	KP898052	278	276/278	99	-	-	-	0.008	-	0.004	0.001
Unidentified sp. 3980_1986	Ascomycota	KT196699	245	239/245	98	-	-	-	0.008	-	0.004	0.001
Chytridiomycota sp. 3980_1989	Chytridiomycota	MH451284	334	333/334	99	-	-	-	0.004	0.004	0.004	0.001
Cortinarius torvus	Basidiomycota	AY669668	281	280/281	99	-	-	-	0.008	-	0.004	0.001
Unidentified sp. 3980_1994	Basidiomycota	KX220737	283	258/300	86	-	-	-	0.008	-	0.004	0.001
Unidentified sp. 3980_1996	Basidiomycota	MH451807	324	42/43	98	-	0.003	0.002	-	-	-	0.001
Unidentified sp. 3980_1999	Ascomycota	KF297165	243	220/247	89	-	-	-	0.008	-	0.004	0.001
Unidentified sp. 3980_2000	Ascomycota	KU063809	261	259/261	99	0.004	-	0.002	-	-	-	0.001
Unidentified sp. 3980_2001	Ascomycota	KY462667	254	180/199	90	0.004	-	0.002	-	-	-	0.001
Unidentified sp. 3980_2003	Ascomycota	MH451801	299	257/301	85	-	-	-	0.008	-	0.004	0.001
Unidentified sp. 3980_2004	Ascomycota	JN032535	242	240/241	99	-	0.003	0.002	-	-	-	0.001
Cladophialophora sp. 3980_2005	Ascomycota	AB986415	273	267/275	97	-	-	-	-	0.008	0.004	0.001
Unidentified sp. 3980_2006	Ascomycota	LC034180	247	79/88	90	-	-	-	0.008	-	0.004	0.001
Chaetosphaeria sp. 3980_2008	Ascomycota	GQ219819	241	229/233	98	-	0.003	0.002	-	-	-	0.001
Podospora sp. 3980_2010	Ascomycota	HG935997	247	246/247	99	0.002	0.002	0.002	-	-	-	0.001
Unidentified sp. 3980_2012	Ascomycota	MG207393	254	254/254	100	-	0.003	0.002	-	-	-	0.001
Unidentified sp. 3980_2014	Ascomycota	KY742560	565	267/268	99	0.004	-	0.002	-	-	-	0.001
Unidentified sp. 3980_2023	Ascomycota	KY742593	544	262/262	100	-	0.002	0.001	-	0.004	0.002	0.001
Unidentified sp. 3980_2024	Ascomycota	MF182038	244	230/230	100	-	0.003	0.002	-	-	-	0.001
Polyporales sp. 3980_2025	Basidiomycota	MF485733	265	262/265	99	-	-	-	-	0.008	0.004	0.001
Dothideomycetes sp. 3980_2027	Ascomycota	KX908796	248	247/249	99	-	-	-	-	0.008	0.004	0.001
Typhula phacorrhiza	Basidiomycota	AB267392	342	340/342	99	-	-	-	-	0.008	0.004	0.001
Unidentified sp. 3980_2030	Basidiomycota	KF267047	619	333/337	99	-	-	-	-	0.008	0.004	0.001
Unidentified sp. 3980_2032	Basidiomycota	KY810468	611	340/341	99	0.004	-	0.002	-	-	-	0.001
Unidentified sp. 3980_2033	Mucoromycota	KC965558	212	200/215	93	-	-	-	-	0.004	0.004	0.001
Inocybe sp. 3980_2034	Basidiomycota	MK607039	296	288/299	96	-	0.003	0.002	-	-	-	0.001
Unidentified sp. 3980_2035	Basidiomycota	FR682265	675	385/388	99	0.004	-	0.002	-	-	-	0.001
Boletopsis leucomelaena	Basidiomycota	DQ408771	298	297/298	99	-	-	-	0.008	-	0.004	0.001
Unidentified sp. 3980_2037	Basidiomycota	MG827649	284	280/284	99	0.004	-	0.002	-	-	-	0.001
Chaetothyriales sp. 3980_2043	Ascomycota	KJ827910	252	244/249	98	0.002	0.002	0.002	-	-	-	0.001
Xylodon spathulatus	Basidiomycota	KY081803	279	278/279	99	-	-	-	0.008	-	0.004	0.001
Unidentified sp. 3980_2047	Basidiomycota	KY810468	688	340/341	99	-	-	-	0.004	0.004	0.004	0.001
Unidentified sp. 3980_2048	Chytridiomycota	MF570928	306	305/306	99	-	0.003	0.002	-	-	-	0.001

Unidentified sp. 3980_2049	Basidiomycota	KY810468	599	339/341	99	-	0.003	0.002	-	-	-	0.001
Unidentified sp. 3980_2050	Ascomycota	MG597461	555	256/258	99	-	0.003	0.002	-	-	-	0.001
Unidentified sp. 3980_2051	Ascomycota	KP897542	247	247/247	100	0.004	-	0.002	-	-	-	0.001
Unidentified sp. 3980_2052	Ascomycota	NR_138377	265	248/266	93	-	-	-	0.008	-	0.004	0.001
Boletales sp. 3980_2053	Basidiomycota	KY826021	305	298/305	98	-	0.003	0.002	-	-	-	0.001
Phanerochaete chrysosporium	Basidiomycota	MH047187	295	295/295	100	-	-	-	0.008	-	0.004	0.001
Unidentified sp. 3980_2060	Ascomycota	KP897304	539	261/263	99	-	-	-	-	0.008	0.004	0.001
Discosia macrozamiae	Ascomycota	MH327820	247	245/247	99	-	-	-	0.008	-	0.004	0.001
Talaromyces rugulosus	Ascomycota	MG714840	266	266/266	100	0.004	-	0.002	-	-	-	0.001
Entoloma fernandae	Basidiomycota	LN850565	297	258/259	99	0.004	-	0.002	-	-	-	0.001
Wilcoxina sp. 3980_2068	Ascomycota	DQ320129	255	251/256	98	0.002	0.002	0.002	-	-	-	0.001
Siepmannia sp. 3980_2069	Mucoromycota	AJ748857	264	256/265	97	-	-	-	0.008	-	0.004	0.001
Unidentified sp. 3980_2070	Ascomycota	KX192890	240	238/240	99	-	0.003	0.002	-	-	-	0.001
Unidentified sp. 3980_2072	Basidiomycota	KP889357	324	310/327	95	0.004	-	0.002	-	-	-	0.001
Hymenoscyphus albidus	Ascomycota	JX976998	241	239/241	99	-	-	-	-	0.008	0.004	0.001
Unidentified sp. 3980_2076	Ascomycota	NR_160218	559	280/281	99	0.002	0.002	0.002	-	-	-	0.001
Unidentified sp. 3980_2080	Mucoromycota	MF942933	598	313/313	100	-	-	-	0.008	-	0.004	0.001
Unidentified sp. 3980_2081	Ascomycota	JQ313099	333	315/364	87	-	-	-	-	0.008	0.004	0.001
Unidentified sp. 3980_2082	Basidiomycota	MF992150	322	290/323	90	-	-	-	-	0.008	0.004	0.001
Unidentified sp. 3980_2083	Ascomycota	MG827663	563	279/281	99	0.002	0.002	0.002	-	-	-	0.001
Unidentified sp. 3980_2088	Ascomycota	HG937011	251	251/251	100	-	-	-	0.008	-	0.004	0.001
Unidentified sp. 3980_2092	Ascomycota	KU062235	594	314/317	99	-	-	-	0.008	-	0.004	0.001
Unidentified sp. 3980_2099	Basidiomycota	FR682244	308	308/308	100	-	-	-	-	0.008	0.004	0.001
Unidentified sp. 3980_2100	Basidiomycota	KT196543	264	240/247	97	-	-	-	0.008	-	0.004	0.001
Unidentified sp. 3980_2101	Ascomycota	KP897261	274	273/274	99	0.004	-	0.002	-	-	-	0.001
Unidentified sp. 3980_2103	Mucoromycota	KX221567	366	354/366	97	0.004	-	0.002	-	-	-	0.001
Unidentified sp. 3980_2108	Chytridiomycota	MF569826	334	327/334	98	-	-	-	-	0.008	0.004	0.001
Cladonia sp. 3980_2109	Ascomycota	KR266621	256	249/256	97	-	-	-	-	0.008	0.004	0.001
Pholiota aurivella	Basidiomycota	AF195603	296	292/297	98	-	-	-	0.008	-	0.004	0.001
Unidentified sp. 3980_2113	Basidiomycota	KY810469	627	342/342	100	-	-	-	0.008	-	0.004	0.001
Unidentified sp. 3980_2114	Basidiomycota	MG231611	221	62/68	91	0.004	-	0.002	-	-	-	0.001
Unidentified sp. 3980_2116	Ascomycota	EU516991	246	244/246	99	0.002	0.002	0.002	-	-	-	0.001
Unidentified sp. 3980_2117	Mucoromycota	MF569741	334	318/342	93	0.002	0.002	0.002	-	-	-	0.001
Unidentified sp. 3980_2119	Ascomycota	KX223133	281	269/285	94	0.004	-	0.002	-	-	-	0.001
Unidentified sp. 3980_2122	Basidiomycota	KY810469	637	340/341	99	-	-	-	-	0.008	0.004	0.001
Unidentified sp. 3980_2123	Ascomycota	MG827896	238	231/239	97	-	-	-	0.008	-	0.004	0.001
Unidentified sp. 3980_2124	Ascomycota	MG161859	257	191/200	96	-	0.002	0.001	-	-	-	0.001
Unidentified sp. 3980_2127	Basidiomycota	KX221795	344	161/196	82	-	-	-	0.008	-	0.004	0.001
Zymoseptoria tritici	Ascomycota	MH862992	254	253/254	99	0.004	-	0.002	-	-	-	0.001
Unidentified sp. 3980_2135	Ascomycota	KP897304	505	262/263	99	-	0.003	0.002	-	-	-	0.001
Unidentified sp. 3980_2136	Ascomycota	MH859602	266	248/267	93	-	-	-	0.008	-	0.004	0.001
Unidentified sp. 3980_2137	Ascomycota	KF617271	236	235/238	99	0.004	-	0.002	-	-	-	0.001
Saccharomycetes sp. 3980_2140	Ascomycota	MH452366	282	280/282	99	0.002	0.002	0.002	-	-	-	0.001
Unidentified sp. 3980_2141	Basidiomycota	MG827414	268	260/269	97	0.002	0.002	0.002	-	-	-	0.001
Unidentified sp. 3980_2142	Ascomycota	FN610869	241	239/241	99	-	-	-	0.008	-	0.004	0.001
Unidentified sp. 3980_2150	Basidiomycota	KP891091	315	309/315	98	0.002	-	0.001	-	0.004	0.002	0.001

Unidentified sp. 3980_2151	Ascomycota	KP897342	261	261/261	100	-	-	-	-	0.008	0.004	0.001
Physisporinus sanguinolentus	Basidiomycota	JX109843	286	283/286	99	-	-	-	-	0.008	0.004	0.001
Unidentified sp. 3980_2155	Basidiomycota	KP891060	634	339/341	99	-	-	-	-	0.008	0.004	0.001
Unidentified sp. 3980_2157	Ascomycota	KP897436	241	241/241	100	-	-	-	0.008	-	0.004	0.001
Unidentified sp. 3980_2158	Ascomycota	MF976202	260	217/266	82	0.004	-	0.002	-	-	-	0.001
Unidentified sp. 3980_2159	Basidiomycota	KM493841	369	368/369	99	-	0.003	0.002	-	-	-	0.001
Unidentified sp. 3980_2162	Ascomycota	JQ313102	242	241/243	99	-	-	-	-	0.008	0.004	0.001
Unidentified sp. 3980_2163	Basidiomycota	KP814341	289	272/292	93	-	0.003	0.002	-	-	-	0.001
Unidentified sp. 3980_2166	Basidiomycota	MG207407	312	310/312	99	0.002	0.002	0.002	-	-	-	0.001
Unidentified sp. 3980_2171	Ascomycota	KY742560	546	266/266	100	-	-	-	-	0.008	0.004	0.001
Unidentified sp. 3980_2172	Ascomycota	JN638280	259	214/262	82	0.002	0.002	0.002	-	-	-	0.001
Sistotrema confluens	Basidiomycota	KX610712	280	278/280	99	-	-	-	-	0.004	0.004	0.001
Lactarius sp. 3980_2184	Basidiomycota	KU924443	356	356/356	100	0.004	-	0.002	-	-	-	0.001
Unidentified sp. 3980_2185	Basidiomycota	KY810469	638	340/342	99	-	-	-	-	0.008	0.004	0.001
Unidentified sp. 3980_2186	Basidiomycota	KP889756	365	207/259	80	0.002	0.002	0.002	-	-	-	0.001
Unidentified sp. 3980_2187	Ascomycota	MG827484	252	248/252	98	0.004	-	0.002	-	-	-	0.001
Unidentified sp. 3980_2189	Basidiomycota	KP897995	304	301/304	99	-	-	-	-	0.008	0.004	0.001
Unidentified sp. 3980_2191	Ascomycota	MG828369	276	258/293	88	-	0.003	0.002	-	-	-	0.001
Unidentified sp. 3980_2192	Basidiomycota	MF942572	223	49/50	98	0.002	0.002	0.002	-	-	-	0.001
Unidentified sp. 3980_2193	Ascomycota	KP891113	561	268/268	100	-	0.003	0.002	-	-	-	0.001
Unidentified sp. 3980_2194	Basidiomycota	MG827813	297	297/297	100	-	-	-	0.008	-	0.004	0.001
Unidentified sp. 3980_2195	Basidiomycota	MF942572	226	49/50	98	-	0.003	0.002	-	-	-	0.001
Unidentified sp. 3980_2199	Ascomycota	MG827599	240	239/240	99	-	0.003	0.002	-	-	-	0.001
Unidentified sp. 3980_2200	Basidiomycota	KY810469	624	337/341	99	0.002	0.002	0.002	-	-	-	0.001
Unidentified sp. 3980_2202	Ascomycota	KM493788	260	252/260	97	-	0.003	0.002	-	-	-	0.001
Pluteus cervinus	Basidiomycota	KJ009640	279	278/279	99	0.002	0.002	0.002	-	-	-	0.001
Unidentified sp. 3980_2204	Basidiomycota	EU690387	327	314/319	98	-	-	-	-	0.008	0.004	0.001
Unidentified sp. 3980_2206	Ascomycota	KT197148	257	214/216	99	-	-	-	0.004	0.004	0.004	0.001
Unidentified sp. 3980_2209	Cryptomycota	KM493320	267	252/268	94	-	-	-	0.008	-	0.004	0.001
Tubaria sp. 3980_2213	Basidiomycota	DQ989327	294	242/256	95	0.004	-	0.002	-	-	-	0.001
Unidentified sp. 3980_2215	Ascomycota	KT957775	252	229/255	90	0.004	-	0.002	-	-	-	0.001
Unidentified sp. 3980_2218	Basidiomycota	KX498040	320	317/320	99	-	-	-	-	0.008	0.004	0.001
Unidentified sp. 3980_2219	Chytridiomycota	MF570979	557	35/35	100	-	-	-	-	0.008	0.004	0.001
Unidentified sp. 3980_2221	Ascomycota	MH863586	249	219/254	86	-	-	-	-	0.008	0.004	0.001
Unidentified sp. 3980_2224	Ascomycota	KM493903	238	230/238	97	0.004	-	0.002	-	-	-	0.001
Unidentified sp. 3980_2225	Basidiomycota	KU062040	536	262/266	98	-	-	-	-	0.004	0.004	0.001
Unidentified sp. 3980_2226	Ascomycota	MF347886	239	236/240	98	-	-	-	0.008	-	0.004	0.001
Unidentified sp. 3980_2228	Ascomycota	KX195668	314	296/314	94	0.004	-	0.002	-	-	-	0.001
Pseudeurotium zonatum	Ascomycota	MH865205	242	242/242	100	0.004	-	0.002	-	-	-	0.001
Unidentified sp. 3980_2233	Ascomycota	KM493551	272	272/272	100	-	-	-	-	0.008	0.004	0.001
Unidentified sp. 3980_2235	Basidiomycota	HE605232	674	384/388	99	-	-	-	-	0.008	0.004	0.001
Sebacina sp. 3980_2239	Basidiomycota	HE687125	285	284/285	99	0.004	-	0.002	-	-	-	0.001
Unidentified sp. 3980_2240	Basidiomycota	KP892185	345	342/346	99	-	-	-	-	0.008	0.004	0.001
Lecania sp. 3980_2242	Ascomycota	AM504067	246	206/207	99	-	-	-	-	0.008	0.004	0.001
Unidentified sp. 3980_2244	Ascomycota	KU559742	247	219/251	87	-	0.003	0.002	-	-	-	0.001
Unidentified sp. 3980_2245	Ascomycota	MG827719	257	255/258	99	-	-	-	-	0.008	0.004	0.001

Unidentified sp. 3980_2246	Ascomycota	KP898205	257	252/257	98	-	0.003	0.002	-	-	-	0.001
Unidentified sp. 3980_2247	Mucoromycota	KX196137	357	311/362	86	-	0.003	0.002	-	-	-	0.001
Unidentified sp. 3980_2248	Ascomycota	GU564975	257	238/239	99	-	0.003	0.002	-	-	-	0.001
Unidentified sp. 3980_2249	Ascomycota	JF300589	242	237/243	98	0.002	0.002	0.002	-	-	-	0.001
Flammula ochroleuca	Basidiomycota	MH856604	298	294/298	99	0.002	0.002	0.002	-	-	-	0.001
Unidentified sp. 3980_2253	Ascomycota	KT195274	267	241/243	99	0.002	0.002	0.001	-	-	-	0.001
Tomentella lilacinogrisea	Basidiomycota	JX630832	314	312/314	99	0.002	0.002	0.002	-	-	-	0.001
Unidentified sp. 3980_2257	Ascomycota	KP897526	253	252/253	99	0.004	-	0.002	-	-	-	0.001
Unidentified sp. 3980_2260	Ascomycota	KX194950	242	242/242	100	-	0.003	0.002	-	-	-	0.001
Unidentified sp. 3980_2261	Ascomycota	KX223186	250	249/250	99	-	0.003	0.002	-	-	-	0.001
Unidentified sp. 3980_2263	Basidiomycota	MF942572	224	49/50	98	0.004	-	0.002	-	-	-	0.001
Unidentified sp. 3980_2264	Basidiomycota	KP897671	320	317/320	99	0.002	0.002	0.002	-	-	-	0.001
Unidentified sp. 3980_2265	Basidiomycota	KP897295	289	246/306	80	-	-	-	0.008	-	0.004	0.001
Unidentified sp. 3980_2266	Ascomycota	JX043012	270	234/283	83	-	-	-	-	0.008	0.004	0.001
Unidentified sp. 3980_2267	Basidiomycota	KC588717	319	314/319	98	-	-	-	0.008	-	0.004	0.001
Tephrocybe sp. 3980_2269	Basidiomycota	HF675301	312	309/312	99	-	-	-	-	0.008	0.004	0.001
Penicillium roseopurpureum	Ascomycota	MH865745	263	262/263	99	0.002	0.002	0.002	-	-	-	0.001
Unidentified sp. 3980_2280	Ascomycota	KP897433	278	278/278	100	-	-	-	0.008	-	0.004	0.001
Unidentified sp. 3980_2281	Cryptomycota	JF300384	225	224/225	99	-	-	-	-	0.008	0.004	0.001
Unidentified sp. 3980_2283	Ascomycota	KU061374	242	234/244	96	0.002	0.002	0.002	-	-	-	0.001
Unidentified sp. 3980_2284	Ascomycota	FN298757	242	207/246	84	-	-	-	0.008	-	0.004	0.001
Unidentified sp. 3980_2286	Basidiomycota	KX194562	298	297/298	99	-	-	-	0.008	-	0.004	0.001
Unidentified sp. 3980_2287	Ascomycota	KX221061	401	375/406	92	0.002	0.002	0.002	-	-	-	0.001
Unidentified sp. 3980_2290	Basidiomycota	KX194806	309	287/311	92	-	-	-	0.004	0.004	0.004	0.001
Thelephoraceae sp. 3980_2294	Basidiomycota	KP403037	312	310/313	99	0.002	0.002	0.002	-	-	-	0.001
Unidentified sp. 3980_2295	Basidiomycota	KX222879	359	358/359	99	0.002	0.002	0.002	-	-	-	0.001
Unidentified sp. 3980_2296	Ascomycota	KP891561	246	245/246	99	0.002	0.002	0.002	-	-	-	0.001
Unidentified sp. 3980_2298	Ascomycota	HM240000	242	241/242	99	-	-	-	0.008	-	0.004	0.001
Unidentified sp. 3980_2302	Ascomycota	KX776744	239	234/235	99	0.002	0.002	0.002	-	-	-	0.001
Cotylidia carpatica	Basidiomycota	MF319060	297	296/297	99	0.002	0.002	0.002	-	-	-	0.001
Unidentified sp. 3980_2305	Ascomycota	KP891415	265	265/265	100	0.002	0.002	0.002	-	-	-	0.001
Unidentified sp. 3980_2306	Ascomycota	DQ421163	307	307/307	100	0.002	0.002	0.002	-	-	-	0.001
Unidentified sp. 3980_2310	Basidiomycota	KM493568	315	314/315	99	-	-	-	0.008	-	0.004	0.001
Unidentified sp. 3980_2311	Ascomycota	KX147893	533	274/278	99	-	0.002	0.001	-	0.004	0.002	0.001
Unidentified sp. 3980_2314	Ascomycota	KY006643	241	240/241	99	-	-	-	0.004	0.004	0.004	0.001
Unidentified sp. 3980_2318	Ascomycota	KT219383	265	231/258	90	-	-	-	-	0.008	0.004	0.001
Therrya pini	Ascomycota	MH857568	241	239/241	99	-	-	-	-	0.008	0.004	0.001
Unidentified sp. 3980_2321	Ascomycota	MG827446	244	228/245	93	-	-	-	-	0.008	0.004	0.001
Unidentified sp. 3980_2325	Chytridiomycota	KX222997	387	384/387	99	0.002	0.002	0.002	-	-	-	0.001
Unidentified sp. 3980_2326	Basidiomycota	MF976114	254	43/44	98	-	-	-	0.008	-	0.004	0.001
Unidentified sp. 3980_2328	Ascomycota	KU063679	502	255/259	98	-	-	-	-	0.008	0.004	0.001
Unidentified sp. 3980_2329	Ascomycota	MF181946	236	218/239	91	0.002	0.002	0.002	-	-	-	0.001
Xylodon raduloides	Basidiomycota	MH880224	279	277/279	99	0.002	0.002	0.002	-	-	-	0.001
Unidentified sp. 3980_2333	Chytridiomycota	KX194417	344	276/346	80	0.002	0.002	0.002	-	-	-	0.001
Chroogomphus rutilus	Basidiomycota	MG457849	333	332/333	99	-	-	-	-	0.008	0.004	0.001
Unidentified sp. 3980_2336	Basidiomycota	HQ850127	358	323/365	88	0.002	0.002	0.002	-	-	-	0.001

Unidentified sp. 3980_2337	Basidiomycota	KY810473	626	341/342	99	-	-	-	-	0.008	0.004	0.001
Unidentified sp. 3980_2338	Basidiomycota	MF570059	218	215/218	99	0.004	-	0.002	-	-	-	0.001
Unidentified sp. 3980_2339	Basidiomycota	MF569219	337	226/264	86	0.002	0.002	0.002	-	-	-	0.001
Unidentified sp. 3980_2340	Ascomycota	KU063112	244	244/246	99	-	-	-	0.008	-	0.004	0.001
Unidentified sp. 3980_2342	Basidiomycota	KY810469	617	340/341	99	-	-	-	-	0.004	0.004	0.001
Unidentified sp. 3980_2344	Basidiomycota	GU328582	251	228/251	91	-	-	-	0.004	0.004	0.004	0.001
Unidentified sp. 3980_2346	Basidiomycota	MF570933	469	77/80	96	-	-	-	-	0.008	0.004	0.001
Unidentified sp. 3980_2347	Basidiomycota	KY103858	260	228/271	84	0.001	0.003	0.002	-	-	-	0.001
Unidentified sp. 3980_2348	Ascomycota	KP897304	549	262/263	99	0.002	0.002	0.002	-	-	-	0.001
Unidentified sp. 3980_2350	Basidiomycota	KX196116	345	319/347	92	-	-	-	0.008	-	0.004	0.001
Unidentified sp. 3980_2351	Ascomycota	MF484666	395	318/400	80	0.002	0.002	0.002	-	-	-	0.001
Unidentified sp. 3980_2352	Basidiomycota	KY810469	638	339/341	99	-	0.003	0.002	-	-	-	0.001
Unidentified sp. 3980_2353	Ascomycota	KY742560	548	264/266	99	-	-	-	0.008	-	0.004	0.001
Unidentified sp. 3980_2354	Ascomycota	KU062897	253	253/253	100	0.002	0.002	0.002	-	-	-	0.001
Unidentified sp. 3980_2357	Chytridiomycota	KX220234	313	311/324	96	0.002	0.002	0.002	-	-	-	0.001
Unidentified sp. 3980_2358	Mucoromycota	MF570610	302	67/72	93	-	-	-	0.008	-	0.004	0.001
Unidentified sp. 3980_2359	Basidiomycota	AM902088	561	560/561	99	0.002	0.002	0.002	-	-	-	0.001
Unidentified sp. 3980_2360	Ascomycota	KP897304	247	237/249	95	-	-	-	-	0.008	0.004	0.001
Unidentified sp. 3980_2381	Ascomycota	MF570101	277	256/279	92	-	-	-	-	0.008	0.004	0.001
Unidentified sp. 3980_2442	Ascomycota	MF569908	230	203/232	88	-	0.002	0.001	-	0.004	0.002	0.001
Lecanora compallens	Ascomycota	MG076965	241	236/241	98	-	0.003	0.002	-	-	-	0.001
Psathyrella sp. 3980_2532	Basidiomycota	MK607575	297	297/297	100	-	-	-	0.004	0.004	0.004	0.001
Unidentified sp. 3980_2574	Basidiomycota	JX136513	293	288/293	98	-	-	-	0.008	-	0.004	0.001
Amanita sp. 3980_2627	Basidiomycota	DQ990869	296	296/297	99	0.002	0.002	0.002	-	-	-	0.001
Capronia sp. 3980_2656	Ascomycota	AF284128	263	252/263	96	-	0.003	0.002	-	-	-	0.001
Unidentified sp. 3980_2663	Basidiomycota	MF942572	221	49/50	98	-	0.003	0.002	-	-	-	0.001
Unidentified sp. 3980_2664	Ascomycota	MF570152	237	237/237	100	-	-	-	-	0.008	0.004	0.001
Unidentified sp. 3980_2674	Ascomycota	MG828161	250	250/251	99	0.002	0.002	0.002	-	-	-	0.001
Unidentified sp. 3980_2693	Ascomycota	MG827421	293	293/293	100	-	0.003	0.002	-	-	-	0.001
Wardomyces pulvinatus	Ascomycota	MH858508	282	259/263	98	-	-	-	-	0.004	0.004	0.001
Cordyceps farinosa	Ascomycota	MH864784	255	255/255	100	-	-	-	0.004	0.004	0.004	0.001
Inocybe maritimoides	Basidiomycota	MH024862	280	280/281	99	0.002	0.002	0.002	-	-	-	0.001
Unidentified sp. 3980_2832	Basidiomycota	KM494225	350	64/68	94	0.002	0.002	0.002	-	-	-	0.001
Unidentified sp. 3980_2856	Ascomycota	KP897579	250	250/250	100	-	-	-	-	0.004	0.004	0.001
Unidentified sp. 3980_2863	Ascomycota	KM494356	242	242/242	100	-	0.003	0.002	-	-	-	0.001
Trichaptum abietinum	Basidiomycota	KC581332	297	297/297	100	-	0.003	0.002	-	-	-	0.001
Unidentified sp. 3980_2898	Ascomycota	KP891553	257	238/261	91	0.002	0.002	0.002	-	-	-	0.001
Unidentified sp. 3980_2904	Ascomycota	KM493313	240	240/240	100	-	-	-	-	0.008	0.004	0.001
Unidentified sp. 3980_2906	Ascomycota	KX195711	238	211/239	88	0.002	-	0.001	0.004	-	0.002	0.001
Lodderomyces elongisporus	Ascomycota	MF084289	228	228/228	100	-	-	-	-	0.008	0.004	0.001
Unidentified sp. 3980_2962	Ascomycota	KR266850	236	232/236	98	0.002	0.002	0.002	-	-	-	0.001
Leiosphaerella praeclara	Ascomycota	JF440976	265	265/265	100	-	-	-	0.008	-	0.004	0.001
Unidentified sp. 3980_3071	Ascomycota	KU559718	257	257/257	100	-	-	-	0.008	-	0.004	0.001
Unidentified sp. 3980_3102	Ascomycota	DQ420728	253	246/255	96	0.002	0.002	0.002	-	-	-	0.001
Unidentified sp. 3980_3108	Zoopagomycota	KM494176	238	238/238	100	0.002	0.002	0.002	-	-	-	0.001
Fomes fomentarius	Basidiomycota	MG719676	293	293/293	100	-	-	-	0.008	-	0.004	0.001

Unidentified sp. 3980_3156	Ascomycota	KY006620	238	237/238	99	0.002	-	0.001	-	0.004	0.002	0.001
Unidentified sp. 3980_3222	Mucoromycota	KP889402	373	250/297	84	-	-	-	0.008	-	0.004	0.001
Unidentified sp. 3980_3239	Ascomycota	AM260819	253	245/253	97	0.002	0.002	0.002	-	-	-	0.001
Unidentified sp. 3980_3256	Mucoromycota	MF570700	300	260/303	86	0.002	0.002	0.002	-	-	-	0.001
Cortinarius pallidirimosus	Basidiomycota	KF732581	280	280/280	100	-	-	-	0.008	-	0.004	0.001
Unidentified sp. 3980_3283	Ascomycota	MF976323	259	247/259	95	-	-	-	0.008	-	0.004	0.001
Unidentified sp. 3980_3374	Ascomycota	KX192742	237	201/246	82	0.002	0.002	0.002	-	-	-	0.001
Melanelixia subaurifera	Ascomycota	JX126305	246	208/208	100	0.002	0.002	0.002	-	-	-	0.001
Cladorrhinum sp. 3980_3480	Ascomycota	KU556533	247	247/247	100	0.002	0.002	0.002	-	-	-	0.001
Unidentified sp. 3980_3583	Basidiomycota	KT194535	256	229/232	99	0.002	0.002	0.002	-	-	-	0.001
Unidentified sp. 3980_3590	Basidiomycota	KU063184	236	221/238	93	0.002	0.002	0.002	-	-	-	0.001
Unidentified sp. 3980_3594	Ascomycota	JQ312947	226	226/229	99	-	0.002	0.001	-	0.004	0.002	0.001
Unidentified sp. 3980_3601	Ascomycota	KC222827	253	243/254	96	0.004	-	0.002	-	-	-	0.001
Unidentified sp. 3980_3605	Ascomycota	KT243366	248	56/59	95	-	-	-	0.008	-	0.004	0.001
Unidentified sp. 3980_3676	Basidiomycota	MG827690	262	230/269	86	-	-	-	-	0.008	0.004	0.001
Unidentified sp. 3980_3697	Ascomycota	MG827651	242	241/242	99	0.002	0.002	0.002	-	-	-	0.001
Amphinema sp. 3980_3713	Basidiomycota	KP125659	272	272/272	100	0.004	-	0.002	-	-	-	0.001
Coryneum umbonatum	Ascomycota	MH859114	270	246/252	98	-	0.003	0.002	-	-	-	0.001
Unidentified sp. 3980_3865	Ascomycota	MF569154	266	266/266	100	-	-	-	0.008	-	0.004	0.001
Unidentified sp. 3980_3943	Basidiomycota	KP892081	206	202/206	98	0.002	0.002	0.002	-	-	-	0.001
Nigrograna sp. 3980_3989	Ascomycota	MK627283	245	237/245	97	-	-	-	-	0.004	0.004	0.001
Lyophyllum fumosum	Basidiomycota	HM572541	308	305/308	99	-	0.003	0.002	-	-	-	0.001
Unidentified sp. 3980_4154	Basidiomycota	KP892418	337	336/337	99	-	0.003	0.002	-	-	-	0.001
Unidentified sp. 3980_4270	Ascomycota	KX195497	256	245/260	94	-	-	-	-	0.008	0.004	0.001
Unidentified sp. 3980_4296	Ascomycota	KF297266	270	264/270	98	-	-	-	0.008	-	0.004	0.001
Pterula gracilis	Basidiomycota	MH861903	297	297/297	100	-	0.003	0.002	-	-	-	0.001
Unidentified sp. 3980_4348	Ascomycota	MG827855	249	240/249	96	-	-	-	0.004	0.004	0.004	0.001
Cutaneotrichosporon curvatus	Basidiomycota	MK267710	263	263/263	100	0.002	0.002	0.002	-	-	-	0.001
Delphinella sp. 3980_4408	Ascomycota	MH860318	255	247/256	96	0.002	0.002	0.002	-	-	-	0.001
Piskurozyma silvicola	Basidiomycota	KY104663	318	318/318	100	-	-	-	0.008	-	0.004	0.001
Pleosporales sp. 3980_4432	Ascomycota	MF486763	243	241/243	99	-	-	-	-	0.004	0.004	0.001
Unidentified sp. 3980_4463	Ascomycota	KX194731	270	256/270	95	0.002	0.002	0.002	-	-	-	0.001
Unidentified sp. 3980_4503	Basidiomycota	KM493249	275	275/275	100	0.002	0.002	0.002	-	-	-	0.001
Unidentified sp. 3980_4512	Basidiomycota	KM493373	205	190/207	92	-	-	-	0.008	-	0.004	0.001
Unidentified sp. 3980_4527	Basidiomycota	KU559652	307	299/307	97	0.004	-	0.002	-	-	-	0.001
Unidentified sp. 3980_4559	Ascomycota	KX776621	239	236/237	99	-	-	-	-	0.008	0.004	0.001
Unidentified sp. 3980_4566	Ascomycota	KP891796	247	233/249	94	-	-	-	-	0.008	0.004	0.001
Hymenoscyphus scutula	Ascomycota	AY789432	241	236/241	98	0.004	-	0.002	-	-	-	0.001
Unidentified sp. 3980_4648	Cryptomycota	KX221271	291	34/34	100	0.002	0.002	0.002	-	-	-	0.001
Unidentified sp. 3980_4775	Mucoromycota	KU188697	328	321/330	97	-	-	-	-	0.008	0.004	0.001
Unidentified sp. 3980_4776	Chytridiomycota	KP888041	355	60/62	97	-	-	-	0.004	0.004	0.004	0.001
Unidentified sp. 3980_4807	Ascomycota	KX193408	245	225/246	91	0.004	-	0.001	-	-	-	0.001
Unidentified sp. 3980_4826	Chytridiomycota	KX222048	333	321/334	96	0.004	-	0.002	-	-	-	0.001
Unidentified sp. 3980_4904	Ascomycota	KX221505	245	243/245	99	0.002	0.002	0.002	-	-	-	0.001
Conocybe sp. 3980_4916	Basidiomycota	JX968189	295	286/295	97	-	-	-	0.008	-	0.004	0.001
Unidentified sp. 3980_4917	Ascomycota	MF976756	237	222/237	94	0.004	-	0.002	-	-	-	0.001

Unidentified sp. 3980_4944	Basidiomycota	KU062247	302	289/292	99	0.002	0.002	0.002	-	-	-	0.001
Unidentified sp. 3980_4957	Basidiomycota	MF570613	284	279/284	98	-	-	-	0.008	-	0.004	0.001
Unidentified sp. 3980_4995	Ascomycota	JX042991	210	201/209	96	0.002	0.002	0.002	-	-	-	0.001
Unidentified sp. 3980_5002	Basidiomycota	KP897313	317	317/317	100	-	-	-	0.008	-	0.004	0.001
Unidentified sp. 3980_5026	Ascomycota	KM493494	242	242/242	100	-	-	-	0.008	-	0.004	0.001
Unidentified sp. 3980_5107	Chytridiomycota	KX220876	295	291/295	99	0.002	0.002	0.002	-	-	-	0.001
Unidentified sp. 3980_5197	Ascomycota	KX194252	240	240/240	100	-	-	-	0.008	-	0.004	0.001
Unidentified sp. 3980_5234	Ascomycota	KC456742	255	250/255	98	-	-	-	0.008	-	0.004	0.001
Unidentified sp. 3980_5253	Ascomycota	KU062159	249	248/250	99	-	-	-	-	0.004	0.002	0.001
Cortinarius talus	Basidiomycota	KJ421141	293	293/293	100	-	-	-	0.008	-	0.004	0.001
Unidentified sp. 3980_5318	Ascomycota	KP897540	247	233/248	94	-	-	-	-	0.004	0.004	0.001
Unidentified sp. 3980_5353	Ascomycota	KC965650	241	233/241	97	-	-	-	-	0.008	0.004	0.001
Unidentified sp. 3980_5384	Ascomycota	KM494175	417	385/435	89	-	-	-	0.008	-	0.004	0.001
Inocybe sp. 3980_5423	Basidiomycota	MF352732	274	273/274	99	-	-	-	0.008	-	0.004	0.001
Unidentified sp. 3980_5427	Ascomycota	JQ313103	232	231/232	99	0.002	-	0.002	-	-	-	0.001
Unidentified sp. 3980_5431	Ascomycota	EU489947	233	200/252	79	-	0.003	0.002	-	-	-	0.001
Unidentified sp. 3980_5440	Ascomycota	KT220100	258	256/256	100	0.002	-	0.001	-	0.004	0.002	0.001
Unidentified sp. 3980_5498	Basidiomycota	KP897500	268	259/268	97	-	0.003	0.002	-	-	-	0.001
Unidentified sp. 3980_5558	Mucoromycota	KX193265	341	340/341	99	0.002	-	0.001	0.002	0.002	0.002	0.001
Urocystis agropyri	Basidiomycota	KX057795	357	357/357	100	0.004	-	0.002	-	-	-	0.001
Unidentified sp. 3980_5610	Ascomycota	MH411229	246	245/246	99	-	-	-	0.008	-	0.004	0.001
Lophotrichus sp. 3980_5660	Ascomycota	MF782703	263	250/265	94	0.002	0.002	0.002	-	-	-	0.001
Unidentified sp. 3980_5722	Ascomycota	MF570700	292	255/306	83	-	-	-	0.008	-	0.004	0.001
Unidentified sp. 3980_5734	Ascomycota	KT881270	239	237/239	99	-	-	-	-	0.008	0.004	0.001
Unidentified sp. 3980_5746	Ascomycota	KX195534	250	250/250	100	-	-	-	0.008	-	0.004	0.001
Aspergillus sp. 3980_5778	Ascomycota	MH102106	261	261/261	100	0.002	0.002	0.001	-	-	-	0.001
Cortinarius sp. 3980_5782	Basidiomycota	DQ481853	298	296/298	99	0.004	-	0.002	-	-	-	0.001
